# Local dimensionality determines imaging speed in localization microscopy

**DOI:** 10.1038/ncomms13558

**Published:** 2017-01-12

**Authors:** Patrick Fox-Roberts, Richard Marsh, Karin Pfisterer, Asier Jayo, Maddy Parsons, Susan Cox

**Affiliations:** 1 Randall Division, King's College London, New Hunts House, Guy's Campus, London SE1 1UL, UK

## Abstract

Localization microscopy allows biological samples to be imaged at a length scale of tens of nanometres. Live-cell super-resolution imaging is rare, as it is generally assumed to be too slow for dynamic samples. The speed of data acquisition can be optimized by tuning the density of activated fluorophores in each time frame. Here, we show that the maximum achievable imaging speed for a particular structure varies by orders of magnitude, depending on the sample dimensionality (that is, whether the sample is more like a point, a strand or an extended structure such as a focal adhesion). If too high an excitation density is used, we demonstrate that the analysis undergoes silent failure, resulting in reconstruction artefacts. We are releasing a tool to allow users to identify areas of the image in which the activation density was too high and correct for them, in both live- and fixed-cell experiments.

## Abstract (web summary)

Localisation microscopy enables nanometre-scale imaging of biological samples, but the method is too slow to use on dynamic systems. Here, the authors develop a mathematical model that optimises the number of frames required and estimates the maximum speed for super-resolution imaging.

The ability of fluorescence microscopy to image live cells has made it one of the most popular cell biology tools. Localization based techniques (PALM^1^, fPALM^2^ and STORM^3^) boost the resolution down to tens of nanometres by identifying fluorophore positions from a large number of time frames and reconstructing a super-resolution image. Unlike most light microscopy techniques, the resolution in localization microscopy is not determined solely by the optical set up. Instead, the resolution of the final reconstructed image depends on how the sample is labelled, how dense the fluorophores are^4^,^5^,^6^,^7^ and the precision with which a fluorophore can be localized, which in turn depends on the number of photons emitted by the molecule and the background level^8^. The density of the fluorophores will also affect the optimum blinking rate for an experiment^9^. The number of photons collected per molecule in an experiment on a given sample is determined by the laser power used and optics available, and the imaging speed is determined by the camera^10^.

As many cell processes are highly dynamic, when performing live-cell localization microscopy it is important to acquire the data necessary for a super-resolution reconstruction of a particular resolution as quickly as possible. The two most controllable factors in a live-cell experiment are usually the activation density, which is the average number of fluorophores that are activated in a time frame, and the speed at which time frames are acquired.

The speed at which time frames are acquired can be increased by using photoswitchable fluorophores. The switching rate of these fluorophores can be precisely controlled and so matched to the camera frame rate. By using high illumination intensities and short exposure times, the speed at which data can be acquired is increased^11^. In some cases the imaging speed will be limited by the properties of the fluorophore—using organic fluorophore pairs, it has been shown that the switching rate^12^ can limit the speed at which images of a certain number of molecules can be acquired.

One can also consider how many fluorophores should be activated per time frame, to minimize the number of time frames needed. The two extreme cases are obviously undesirable: if the activation density is too low it will take a long time to acquire the data, because many time frames are required, and if the activation density is too high the images of the fluorophores will overlap too much and their positions cannot be accurately fitted.

The most commonly proposed solution to the problem of imaging in live cells is to allow denser activation and analyse the data using a more sophisticated algorithm that can fit overlapping fluorophores^13^,^14^,^15^,^16^,^17^. These methods have pushed the time needed to acquire the data necessary for a particular resolution down dramatically. However, the maximum speed at which this data can be acquired is still limited and must currently be determined by trial and error.

As well as the above considerations for designing and performing the experiment, it is also important to have a robust metric for evaluating how well the data analysis algorithm has worked and measuring the resolution of the resulting reconstructed image afterwards. However, the determination of resolution is not a trivial matter for localization microscopy, as the reconstructed image is not a linear representation of the photons emitted by the sample (as is the case for most optical microscopy techniques). Therefore, considerable effort has been put into evaluating localization microscopy algorithms and into finding fair ways to evaluate localization microscopy images. For the evaluation of algorithms, the most standard method has been to perform tests on simulations of a known ground truth structure and then test how well that structure is reproduced^12^,^18^. Unfortunately, for real experiments ground truth is not available.

An evaluation using the data alone is possible using Fourier Ring Correlation (FRC)^7^, which splits the data into two sets of localized positions and evaluates how well they are correlated in the Fourier domain. However, as the authors point out, systematic errors in localization can lead to an FRC value, which does not reflect the true resolution. Such systematic errors can arise when the activation density is too high. Therefore, for experimental data, it is particularly important to achieve an activation density that allows the resolution to be calculated reliably. For live-cell experiments, this must be balanced against the need to operate at high speed, to allow dynamic samples to be imaged.

Here we demonstrate by considering a simplified mathematical model, but also via simulations and both live- and fixed-cell experiments, that the dimensionality of the sample at the length scale of the point spread function (PSF) changes the best imaging speed that can be achieved, by around an order of magnitude for an integer increase in dimensionality. The dimensionality of the sample is determined by its structure: small point-like objects much smaller than the size of the PSF are zero-dimensional, linear structures such a microtubules are one dimensional (1D) and structures such as a focal adhesion are two dimensional (2D). We show how to calculate, in advance, the minimum number of time frames needed to achieve a given resolution and the density of fluorophore activations in the raw data that will allow that speed to be achieved. This is important, because it is a fundamental constraint on the achievable localization microscopy imaging speed. In addition, we demonstrate a new method of post processing the collected data, which will warn users whether the data fitting is not reliable in certain data sets, or in certain regions of an image. This uses a machine-learning approach, trained via user input, to build a random forest classifier to evaluate the accuracy of each reported fluorophore localization, optimized for that particular data set and localization algorithm.

## Results

### Theory and simulations

We first examine a simple mathematical model of the process by which fluorophore activations are localized. In particular, we focus on the effect of activations that occur close to one another, causing the algorithm to reject them or incorrectly fit to their overlapping PSFs. Here we assume that we are operating in the regime where the resolution is limited by the density of fluorophores in the reconstructed image. This will always apply, as we are aiming to take as few time frames as possible, and as we lower the number of time frames we will always reach a point where the density is the limiting factor^7^.

Consider a sample 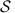, which is to be imaged using localization microscopy. The sample is tagged with fluorophores, which are stochastically activated and can undergo blinking and bleaching. The images of the sample are analysed to find the location of individual emitters. The resolution of the super-resolution reconstruction will then be determined by the localization precision, uncorrected drift and the density of localized emitters. As the first two factors are determined by the equipment and sample, and therefore will vary between experiments, we define the quality 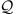 of the super-resolution image as the density of activations on the sample, which are successfully localized by the data analysis. As we are interested in the minimum number of time frames required to achieve a particular resolution, we have assumed that the experiment will operate in the density-limited case. We have therefore taken the number of time frames required to achieve a particular 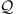 in the region about a point *x* in the sample as a measure of acquisition time, denoted 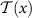.

We assume that for any time frame in the sequence the number of active emitters *N* is Poisson distributed, with a mean of 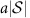. 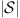 is the size of the tagged area of the sample, whereas *a* is a parameter that represents the probability of an emitter activating per unit area of sample in a given time frame. This models the situation where there is a very large ‘pool' of possible fluorophores, of which a small subset activate in any given time frame and the total proportion that undergo irreversible bleaching is considered negligible. This is a common situation in live-cell imaging due to the short available acquisition time, in contrast to fixed-cell imaging, where the proportion of fluorophores activated and subsequently bleached during the entire acquisition may become significant. The value of *a* is determined by the properties of the fluorophores, the laser power, any activation laser used, the frame rate of the camera and the embedding conditions, among other factors. For a given sample, the situation where *a*=0 corresponds to a probability of zero of observing any activations. As *a* is increased, the average number of activations grows linearly. Initially, so does the number of accurate localized fluorophore positions.

However, if *a* is increased beyond a certain point, although the average number of fluorophore activations will continue to grow, the average number of accurate localizations will plateau and then rapidly decrease. This occurs because the fluorophore PSFs are not sufficiently spatially separated for the algorithm to individually identify them. The necessary degree of separation varies between algorithms (experimentally, we have found it to be around 400–600 nm when using the QuickPALM^19^ or ThunderSTORM^20^ algorithms, with *λ*=488 nm, numerical aperture (NA)=1.4, pixel size 110 nm), but is always non-zero (see Supplementary Note 1 and Supplementary Fig. 1). We model this by assigning an exclusion area 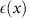 to the active emitter at position *x*. Within this exclusion area, no further activation can occur if the fluorophore is to be successfully localized. This is true also of multi-emitter fitting algorithms, although due to their ability to tolerate significant PSF overlap when localizing molecules the size of this exclusion area is much smaller. We assume 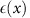 is circular, with radius *r*, where *r* depends on the algorithm used and the PSF.

We assume that the emitters are uniformly distributed across the sample (that is, within 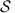), and absent elsewhere. Next, for an active emitter, the probability of falling within 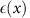 is equal to 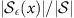, where 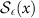 is the intersection between 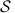 and 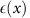 (that is, 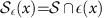). Given that *N* is Poisson distributed, it follows from this that the acquisition time 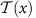 to achieve the desired quality 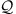 (in terms of localizations per unit area) can be estimated as


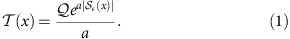


(For derivation see Supplementary Note 2.) Differentiating with respect to *a* shows that the minimum value of 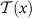 occurs at 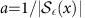 and is equal to 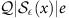.

For real samples, 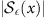 is not usually known. However, as it is equal to the intersection of a circle of radius *r* with the tagged portion of the sample, we can conclude that it will depend strongly on the dimensionality 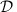 of the sample at the scale *r* and be of the form 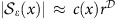, where *c*(*x*) is a position-dependent function, which is approximately constant for small changes in *r* (see Supplementary Note 2). This implies that samples with a high intrinsic dimensionality may take much longer to image than those with a low dimensionality (conversely, if a method can be found to reduce *r*, such as by using a multi emitter fitter, samples with a high intrinsic dimensionality will benefit the most—this is illustrated in Supplementary Fig. 2). More detail on calculating *c*(*x*) and the potential speed of imaging given a particular sample shape is given in Supplementary Note 3.

An example of an approximately 1D sample would be an actin filament or microtubule, whereas an example of a 2D sample would be a focal adhesion (which are generally at least a micrometre wide and several micrometres long). To give an example of how we might calculate the change expected in 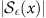, let us consider the case of an actin filament 7 nm wide^21^. This is imaged using localization microscopy and the data analysed using an algorithm with *r*=400 nm. If the target reconstruction density is 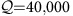 molecules per μm^−2^, then *c*(*x*)≈2 × 7 nm, 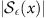
≈0.0056 μm^2^; therefore, 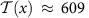 time frames at the optimum value of *a* (the fluorophore activation probability). If we were analysing a 2D sample such as a focal adhesion, which is ≫0.4 μm in all directions, then *c*(*x*)≈*π* and 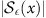
≈0.50 μm^2^. Therefore, 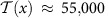 time frames at this sample's optimum value of *a*. Therefore, changing from a 1D to a 2D structure means that it takes almost a factor of 100 longer to acquire the data for a reconstruction of the same resolution.

In live-cell experiments it is often necessary to minimize the data acquisition time, as the sample can move. Therefore, it is desirable to use a high activation density, to maximize the quality of the reconstructed image. However, if the activation density is too high, then fluorophore PSFs in the acquired time frames can be close to each other or overlap. Ideally, in this case the algorithm would exclude these fluorophores from the fitting. There are a number of approaches to achieve this: QuickPALM performs symmetry tests on the fitted PSF, whereas ThunderSTORM can exclude based on the fitted PSF width.

However, in practice it is very difficult to avoid mis-localizations in all cases, in particular given the wide variability in experimental parameters. Our evaluation of localization algorithms indicates that when fitting two nearby fluorophores, there are three possible outcomes: above a certain distance the fluorophores will be successfully localized; below this, there is a region in which two fluorophores are localized but the positions can be biased; and then a distance is reached below which only a single fluorophore is fitted rather than two. Any localization algorithm will return incorrect localizations if the emitting fluorophores are close enough. The higher the activation density, the more mislocalizations will happen.

The errors that these mislocalizations introduce have a systematic bias. For example, on a line type structure the bias will be towards the centre of the line (see Sinkó *et al*.^18^ for a more detailed analysis). This makes individual lines appear sharper, but it is to be noted that this is not an increase in resolution and the ability to distinguish two crossing lines will be degraded. These artefacts are very difficult to detect, as methods that evaluate the resolution without access to the ground truth structure, such as FRC, will incorrectly report a higher resolution (see Supplementary Note 4).

It is clearly desirable to be able to test for this silent failure of the data analysis, where misfits are present in the reconstruction and may change the observed sample structure. However, such testing is challenging, both because of the absence of ground truth information and because of the wide range of background levels, photon numbers per molecule, activation densities and sample structures encountered in real experiments. We have taken a machine-learning approach, in which the user examines a small subset of the localized fluorophore positions selected at random (usually 300 patches) and classifies whether they are correct, background or an incorrect fitting of overlapping fluorophores. For each localization a feature vector is built, based on the pixel values around the localized position and in the corresponding image patches in the previous and next frame, along with all the information returned by the localization algorithm. Using principle component analysis (PCA) this vector is then compressed to only its 20 most significant components. The localizations classified by the user are used to train a random forest classifier^22^, which is then used on the other (unclassified) localizations. As the classifier is trained based on user feedback to recognize the characteristics of an incorrect localization for a particular data set, the classifier tunes its response to both the specific experimental conditions and the algorithm used.

Our method has the advantage that it can, in principle, be used with almost any localization algorithm. The only assumptions made about the algorithm are that it is analysing one frame at a time, it returns a list of individual localization co-ordinates, and that the position of the fluorophore is inferred from characteristics of the local image region. These assumptions hold true for the vast majority of localization microscopy algorithms, with exceptions such as 3B (ref. 16), which outputs a probability density, and SOFI (ref. 16), which outputs an image.

### Verifying the effect of sample dimensionality in simulations

**Figure 1:**
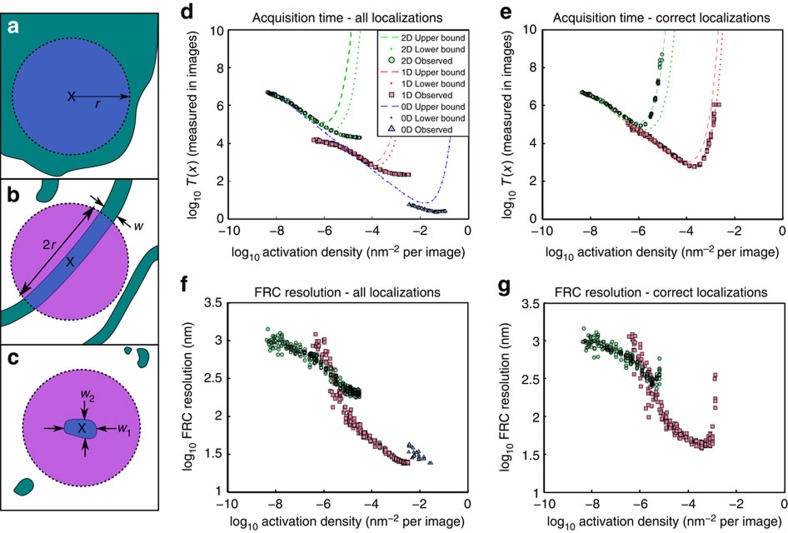
Effect of sample dimensionality on acquisiton time. Demonstrates how local dimensionality changes the acquisition time needed to achieve a particular resolution. (**a**–**c**) Examples of 0D, 1D and 2D sample structures. Samples are shown in green, the exclusion area in purple and the intersection between the two in blue. The exclusion area approximately follows the pattern 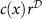 where 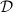 is the local sample dimensionality at scale *r*. (**a**) For a 2D sample, the intersection between the exclusion zone and the sample is given by 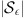
≈*πr*^2^; (**b**) for a 1D sample, it is 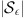
≈2*wr*; (**c**) and for a 0D sample, 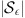
≈*w*_1_*w*_2_. (**d**,**e**) The theoretical and simulated values of the number of acquisition frames *T*(*x*) (to achieve a particular resolution) vary with the activation density *a*. Theoretical values are shown for exclusion radii of 400 nm (dashed line) and 600 nm (dotted line). The localizations returned by the fitting algorithm are displayed in **d** and those determined to be good fits after re-classification are shown in **e**. Only 2D and 1D data were reclassified; as the 0D simulated structures were very small, users were not able to reclassify accurately. An alternative approach is to evaluate the resolution for a given number of frames. This can be done with FRC (**f**,**g**). (**f**) The FRC resolution using all localizations returned by the analysis algorithm and (**g**) the FRC resolution using only those localizations classified as good.

To demonstrate the effect of local dimensionality on the maximum acquisition speed, we simulated structures for 0D, 1D and 2D samples (*λ*=488 nm, NA=1.4, pixel size 110 nm). Fluorophore blinking data were simulated for different activation densities and analysed using ThunderSTORM^20^ (for Fig. 1) and QuickPALM^19^ (see Supplementary Note 5 and Supplementary Fig. 3). For the mathematical theory, the resolution was quantified as the reconstruction density, 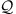. For each type of structure, the theory predicts high numbers of time frames will be required to achieve a given 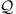 at both low and high activation densities, with a density in between requiring a minimum number of time frames (see Fig. 1d). The number of time frames required and the activation density required, vary between the different structures by more than an order of magnitude for each change in dimensionality. The radius of the exclusion region was estimated to fall in the range 400–600 nm and theoretical acquisition times for these two extremes are shown (it is noteworthy that the exclusion area that intersects the sample varies quadratically with the exclusion radius for a 2D structure and linearly for a 1D structure).

Although the theoretical results showed a clear optimum activation density, fits of simulated data showed the number of frames required to achieve a particular resolution decreased continuously with increasing activation density. This appears both for evaluations using 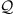 (Fig. 1d) and FRC (see Fig. 1f). (It is noteworthy that FRC is a stochastic technique, which relies on evaluating the correlation of random subsets of the observed localizations and therefore data sets with lower numbers of localizations give FRC results with higher variance.) This difference between theory and simulations arose, because more misfits occur at high activation densities. The misfits lead to artificial sharpening, which improves both the measured 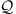 and FRC value (see Supplementary Note 4 and also Supplementary Fig. 4). At very high activation densities, the number of misfitted localizations stabilizes, leading to an apparent plateau in the number of frames required to achieve a particular resolution.

To quantify the effect of misfits, we applied our re-classification technique to divide the localizations into good localizations and mis-fits due to fluorophore overlap, either as individual molecules or as a high background. When only ‘good' localizations were used, the results of the simulations fell within the range of the theoretical predictions (see Fig. 1e), an impressive degree of agreement given the small number of free parameters in the theory, and the simplicity of the user input to the classifier. Interestingly, the 2D structure appears to fall closer to the theoretical result for a 600 nm exclusion radius, whereas the 1D falls closer to the result for the 400 nm exclusion radius. This may indicate that the user labelling the training set was more cautious as to the definition of a good localization when labelling 2D data.

Similarly, when the FRC measure is calculated for only those localizations classified as good, we no longer see a continuous improvement (see Fig. 1g). Although noisy (due to the stochastic nature of FRC calculations and the comparatively small number of good matches at high activation densities), each structure has a clear optimum. This optimum, for which both the values and variance of the FRC is minimized, falls at a slightly higher activation density than that estimated by considering the minimum acquisition time. The optimal density and corresponding best FRC values differ for the 1D and 2D cases, with the optimum FRC of the 1D structure lower than that of the 2D structure. This indicates a better resolution reconstruction for the 1D structure, as expected from the theory.

It is worth noting that we only show classification results for the 1D and 2D case. For our 0D sample, as the simulated structure only consisted of a single 8 × 8 nm region, the localizations were so concentrated that distortions due to multiple fluorophores being mistaken for a single activation were not discernible by the eye. This will not be the case for all 0D structures one encounters in practice—a sample consisting of many point-like structures randomly scattered across the field of view, for example, may feature groupings of points that are sufficiently dense to cause errors in the localization algorithm, whereas still changing the apparent PSF enough that an experienced user can clearly see the difference. In addition, for many applications, errors localizing fluorescent molecules within a 0D structure may be unimportant due to the small absolute size of the actual position error. However, this case does illustrate an important limitation of this method (namely that it can only learn to correct errors that the user can first identify) and to correct for it would require using a different measure of error to that applied to the 2D and 1D cases; hence, we have let the results stand as they are.

Furthermore, it is worth noting that the same basic analysis applies to three-dimensional (3D) localization microscopy, with the caveat that the best acquisition speed is also strongly determined by the sample's orientation with respect to the imaging plane. Further discussion of this is given in the Supplementary Note 6 and also illustrated in Supplementary Fig. 5. A 3D STORM/PALM synthetic data set was simulated in the same manner as the 2D data discussed above, using the astigmatic method to encode depth information, and analysed using QuickPALM. The results for this are shown in Supplementary Fig. 6. It is noteworthy that as the PSF is now stretched along each axis, our machine-learning method of removing false positives is no longer applicable—instead, we approximate this using a Bayesian classifier as described in Supplementary Note 7 (the limitations of this are discussed in Supplementary Note 6).

### Verifying the effect of sample dimensionality in live cells

**Figure 2:**
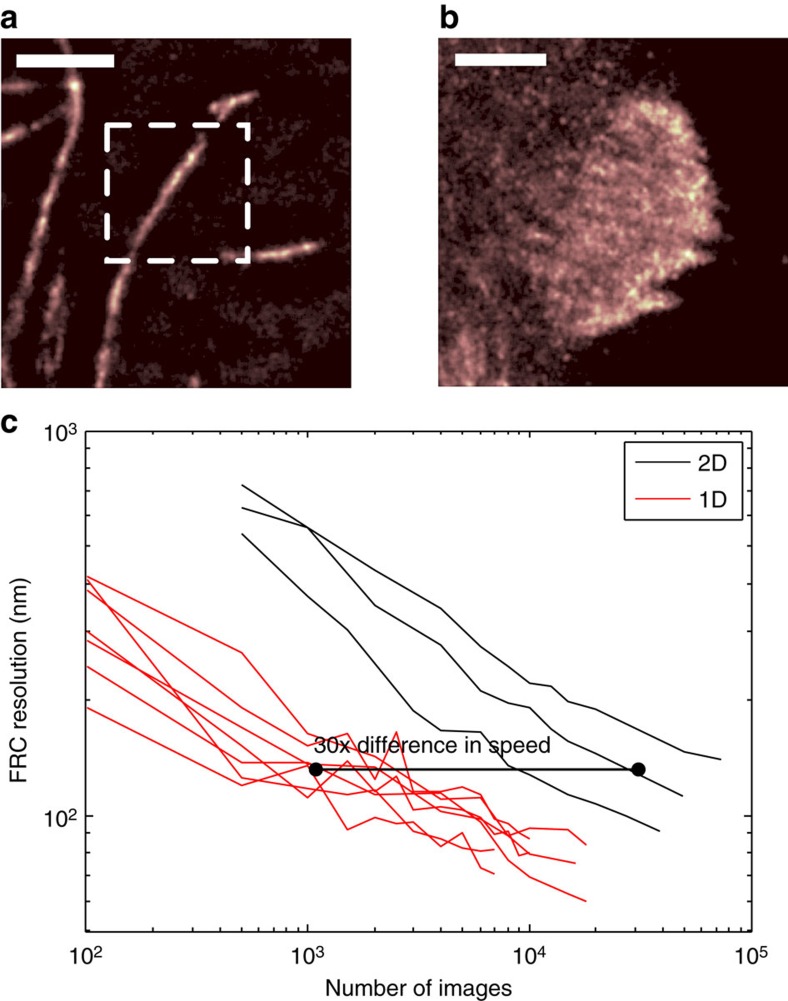
Variation of imaging speed with local sample dimensionality in live cells. Microtubules were used as 1D structure and vinculin in focal adhesions was used as a 2D structure. Reconstructed super-resolution images of the microtubule and focal adhesion samples are shown in **a** and **b**, respectively. The dotted box in **a** shows a representative sampled area and the whole of **b** is a representative sampled area. Areas of different sizes were used to allow selection of areas containing only 1D or 2D structure, and to minimize cutting across the structure to obtain the sample area, which alters the resolution reported by FRC. Scale bar, 1.75 μm in both images. The values of 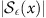 indicate an expected factor of 38 times difference in achievable acquisition speed. (**c**) The FRC resolution value against the number of time frames included in the sequence. Different lines correspond to image sequences of different areas. The observed difference in FRC shows an approximately × 30 difference in resolution. The activation densities were calculated to be ≈0.18 and ≈1.5 activations μm^−2^ for the microtubule and focal adhesion experiments, respectively.

To demonstrate the effect of dimensionality on the time it takes to acquire the data for a particular resolution in live-cell experiments, we used samples expressing the photoswitchable fluorophore mEOS2 (*λ*=584 nm, NA=1.4). Two structures were imaged: microtubules, which have a thickness of around 25 nm (ref. 23) and are therefore approximately 1D (see Fig. 2a), and focal adhesions, which grow to 2−6 μm long and around a micrometre wide^24^, making them 2D (see Fig. 2b). It is noteworthy that focal adhesions do have structure along the *z* axis as well^25^, but in these experiments we are not measuring the z-position of molecules.

We calculated the intersection between both of these structures and the exclusion zone, assuming an exclusion zone radius of 600 nm. For microtubules, which have a thickness of 25 nm (ref. 23), 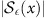
≈0.025*2*0.6=0.03 μm^2^, and for focal adhesions 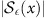
≈π*0.6^2^≈1.13 μm^2^. This gives a ratio between the 2D and 1D values of 38. It is worth noting that this assumes the microtubules are well separated from one another, and that focal adhesions are uniform and large; thus, it ignores edge effects, non-uniform labelling and other causes of density variations not related to the basic structure of the sample.

This prediction can be tested by quantifying the achieved resolution (using FRC) for different lengths of image sequence. This was achieved by analysing successively longer subsets of the collected image sequence. The horizontal displacement of the 1D and 2D data relative to each other (shown in Fig. 2c) indicates that, to achieve the same resolution, the 2D structure consistently needs approximately × 30 more time frames than the 1D structure. This is in general agreement with the calculated 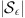 ratio and shows the order of magnitude type scaling observed in our tests on synthetic data sets. Therefore, a localization-based system and algorithm that can acquire the data required for a reconstruction of a certain quality of microtubules in, for example, 10 s, will require 3 min to image a focal adhesion at the same resolution.

### Demonstrating the identification and removal of artefacts

**Figure 3:**
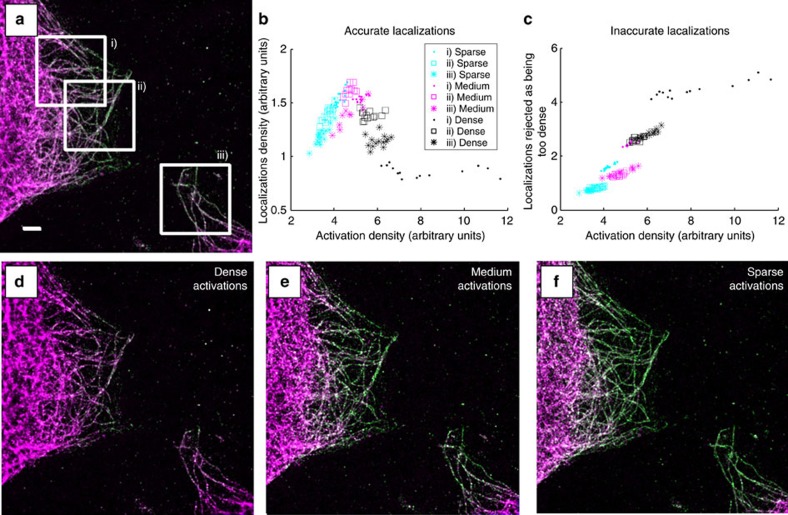
Variation of reconstruction quality with activation density. User-driven reclassification can identify the quality of fits of localization microscopy data analysis. Localization microscopy data were taken of a fixed cell sample, with tubulin labelled with Alexa 647. A sequence of 25,000 time frames of raw data was divided into three subsequences, each of which had a different activation density. The data were classified as either accurate fits to a single fluorophore (yellow–green) or inaccurate (shown in magenta). (**a**) The reconstruction using all three sub-sequences, (**d**) using the dense subsequence, (**e**) the medium and (**f**) the subsequence with the sparsest activation density. Misfits to multiple fluorophores increase as the activation density increases and within a single data set occur more frequently where the microtubules are close together or cross. (**b**) The variation in nominal activation density (estimated from the sum of localization intensities returned by ThunderSTORM, for 1,000 frame sequences) on the horizontal axis, against approximate density of good localizations on the vertical. A clear peak is visible within the achievable range of activation densities. (**c**) The number of localizations classified as too dense in the same manner. Scale bar, 2.5 μm.

Our user-driven localization reclassification provides a method that can indicate whether data are reliable. Microtubules labelled with Alexa 647 were excited with different laser powers so as to give three different levels of activation density. Reconstructions for the high, medium and low activation densities, which have been reclassified with user input, are shown in Fig. 3. Localizations classified as good are shown in yellow–green and misfits are shown in magenta. In the high-density reconstruction, there is a low proportion of good localizations, with most being classified as inaccurate. Localizations classified as too overlapping are concentrated in regions where there are many filaments close together, or at points where microtubules cross (as expected, as these areas will have a higher fluorophore density). The proportion of good localizations increases in the medium-density data set and is highest in the low-density data set.

**Figure 4:**
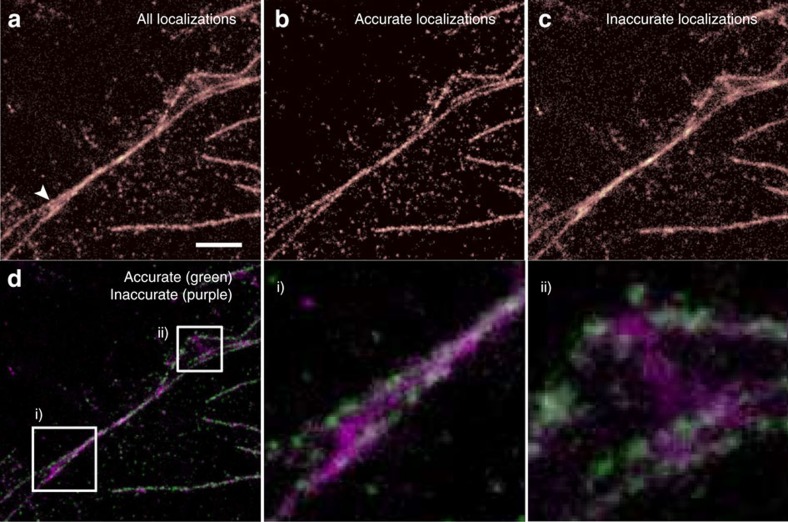
Artefact identification using random forest classifier. Re-classification of the data allows artefacts to be identified and removed. A fixed cell sample, with microtubules labelled with Alexa 647, was used to acquire a localization microscopy data set. (**a**) All localized positions found by the ThunderSTORM, (**b**) only those classified as accurate and (**c**) those classified as inaccurate. Although the structure indicated by an arrow in **a** appears to consist of three strands, the middle one appears only in the image made from misfits due to overlapping fluorophores PSFs, revealing it to be an artefact. The image reconstructed from accurate fits; (**b**) only two strands. (**d**) A false colour image of the same data, with insets (i) and (ii) shown in more detail. Scale bar, 1.5 μm.

In addition, as previously noted, there is a variation in the proportion of the localizations classified as good and bad, for any given excitation density. This is because, unlike in the simulations, the microtubules can be close to one other and can cross over each other in the image. This means that for some data sets, the reconstruction will be mostly high quality but will show artefacts in some regions. For example, when two filaments cross, they can come close enough together that PSFs on the two different strands will frequently overlap in the images. In Fig. 4a we see a reconstruction that shows two longer strands coming together, with a third, shorter strand between them. However, when the data are classified using a Random Forest into accurate/inaccurate localizations (as shown in Fig. 4b,c), we can see that the apparent middle filament arises solely from fits classified as too dense, due to simultaneous activation of a fluorophore in each of the real strands. The accurate/inaccurate localizations are shown together in two colours in Fig. 4d, with insets i and ii illustrating identified artefacts due to inappropriately dense fluorophore activation.

To investigate the impact that activation density has on the reconstruction quality, we took three different regions from the data set shown in Fig. 3a. We used the sum of the fluorophore brightnesses, divided by the median brightness for that activation level (as estimated by ThunderSTORM), as a proxy for the number of activated fluorophores (the accuracy of this proxy is demonstrated in the Supplementary Note 8 and Supplementary Fig. 7). This allows the data from misfitted fluorophores to be taken into account, as they are still fitted, albeit with an incorrect number of fluorophores. The number of activated fluorophores was converted into a density by estimating the area of the microtubules in the image, using a thresholded reconstruction from which small clusters (areas smaller than 10 pixels) had been removed. The calculation was performed for sequences of 1,000 frames. The results (see Fig. 3b) show a peak in the reconstruction density 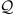 at a certain activation density, whereas the bad fits increase sharply at densities above the optimum (Fig. 3c).

**Figure 5:**
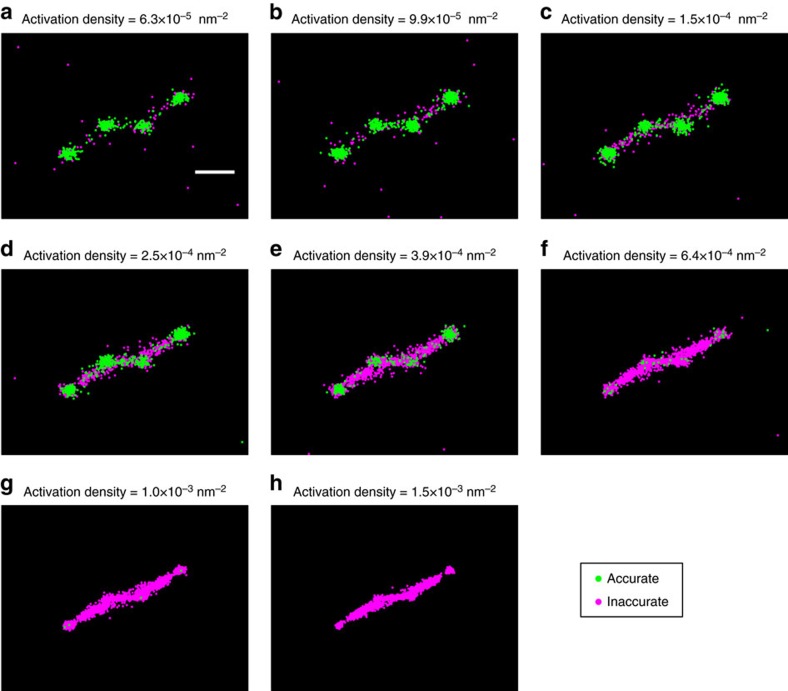
Artefact identification in simulated vesicles. (**a**–**h**) A simulation of imaging four vesicles, based on the test shown in Fig. 18 of Sinkó *et al*.^18^ Four vesicles, with relative positions (0, 0), (200, 200), (200, 400) and (400, 600) (measured in nm), were simulated at different activation densities. Each vesicle is simulated as a uniform circle of radius 30 nm. Localizations classified as accurate are shown in green, inaccurate in magenta. Scale bar, 200 nm.

To further illustrate the capabilities and limitations of our artefact removal method, we replicated the test performed in Fig. 8 of Sinkó *et al*.^18^ This involves simulating four vesicle structures, with *x* and *y* co-ordinates in nanometres of (0, 0), (200, 200), (200, 400) and (400, 600), where each vesicle is represented by a uniform circle of radius 30 nm. Fluorophore blinking and bleaching, at different activation densities, was simulated using the same Markov model as for our earlier tests (*λ*=488 nm, NA=1.4, pixel size=110 nm). The resulting reconstructions are shown in Fig. 5, with localizations classified as accurate shown in green and inaccurate in magenta. The majority of accurate classifications are localized correctly in four clumps, corresponding to the vesicle positions, whereas the localizations classed as inaccurate tend to form bridges between these. However, between the central two vesicles, a number of incorrect localizations forming a bridge have been classified as accurate—we attribute this to their separation being only 200 nm, which is marginally below the separation at which fluorophores can no longer be distinguished by eye. Hence, the random forest classifier cannot be given accurate labels to learn from and it makes errors as expected.

## Discussion

The speed of live-cell localization microscopy is limited by the rate at which information can be transmitted through the system and this in turn depends on the structure of the sample. Thus, the optimum activation density and optimum speed varies from sample to sample, even if the same fluorophore and microscope is used. This is fundamentally different from other microscopy techniques, which are limited by the optics and illumination source, but not the structure of the sample.

The activation density required to achieve the optimum imaging speed and the imaging speed that can be achieved, both vary by roughly an order of magnitude with each increase in the local dimensionality of the sample (that is, from 0D to 1D, to 2D). Therefore, when benchmarking the performance of live-cell localization microscopy techniques, the same type of structure must be used for any comparison to be meaningful. As live-cell localization microscopy becomes a more widely used technique, our method will allow users to calculate the achievable imaging speed for their structure in advance and to optimize their acquisition parameters to achieve the best possible results.

Our results also have broader implications for the evaluation of localization microscopy performance. Methods that seek to maximize the number of returned localizations or to minimize the measured FRC resolution or the full width at half maximum of the reconstruction can be prone to overestimating performance on real data, as artificial sharpening will be reported as improved resolution. By re-classifying the data, the presence of fits to overlapping PSFs can be identified. This allows users to identify whether certain regions of an image, or indeed the whole data set, contain an unacceptable number of misfits. Our method is applicable to the vast majority of localization algorithms and can be used alongside any of the current resolution determination techniques to show when data are reliable and how that reliability varies across the image. Such a confidence metric is a key component to bringing localization microscopy into the wider community.

## Methods

### Simulation parameters

Data were generated for different dimensionalities of sample, to evaluate the effect of dimensionality on the time taken to acquire the data necessary for a particular reconstruction quality (the value chosen was 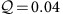 localizations per nm^2^). The resulting image sequences were analysed using ThunderSTORM^20^ and QuickPALM^19^ (discussed in Supplementary Note 5).

When generating samples, two factors were varied: the shape of the sample and the activation density *a*. Three different sample shapes were examined, each one uniformly tagged and localized in 2D using the standard QuickPALM method. These sample shapes were an 8 × 8 nm^2^ ‘0D' point, an 8 × 3,520 nm^2^ ‘1D' line and a 3,520 × 3,520 nm^2^ ‘2D' plane. The tagging density was set to 0.5 nm^−2^ throughout.

The blinking and bleaching dynamics of each tag were simulated using a Markov chain (see Supplementary Note 9 and Supplementary Table 1, which gives the transition probabilities). The activation density was controlled by altering the tagging density (that is, the number of emitters per unit volume or area) and the probability of each tag activating. The tagging density was varied to ensure that there were a large number of emitters available for activation, whereas still allowing the simulations to be run in a reasonable amount of time—this was most important for the 3D simulation, where the difference in volume between the largest and smallest sample spans six orders of magnitude. The activation probability was treated as a tunable parameter, which when varied resulted in different values of *a*. For each fluorophore activation probability and structure, ten different data sets were simulated using different random number seeds. Finally, random photon emissions, detection and a readout error simulating an electron multiplying charge-coupled device camera was applied (see Supplementary Note 9), assuming a wavelength of 488 nm and a NA of 1.4 objective (giving a *λ*/*Na* ratio of 348 nm). A pixel size of 110 nm was used. A fluorophore that was active for the entire duration of a time frame would emit ≈450 photons.

In all cases, our tests estimated the number of time frames that must be analysed to reach a target localization density in the reconstruction. It proved impractical to do this directly by creating longer and longer image sequences, until the target density 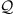 was reached, as the estimated time taken to achieve this varied by multiple orders of magnitude between samples. Instead, all samples were generated with sequence length 1,000 time frames and from this the average number of successful localizations per time frame calculated. Given that the density scales linearly with the number of samples, this figure was then used to extrapolate the number of time frames required to reach 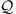, assuming that this average value is maintained throughout acquisition.

We found that the minimum separation between two reported localizations was ≈440 nm (see Supplementary Note 1). This implies that 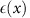 can be approximated as a circle of radius 440 nm. Using this, we estimated several approximate values of 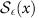 as follows: for the 0D sample, it was assumed that the sample 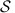 fell entirely within 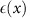 for all readings and therefore 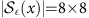 nm^2^; for the 1D sample, it was assumed that the intersection between 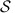 and 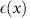 approximately formed a rectangle, with length equal to the diameter of 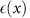 and width equal to the filament thickness (8 nm), giving 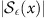
=8 × 880 nm^2^, and for the 2D sample, it was assumed that 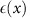 fell entirely within 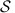 (ignoring edge effects), resulting in 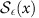 being identical to 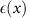 and hence giving 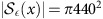 nm^2^.

To calculate 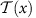, it is necessary to set a target reconstruction density 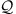. Results are shown for 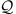
=1/25 nm^−2^ (1 localization per 5 × 5 nm^2^ region). It is also necessary to estimate *a*. This was performed by averaging the number of active emitters per time frame across all time frames in the sequence and dividing this by the tagged area per volume of the sample.

### Estimating the size of the exclusion region

To determine the size of the exclusion region, simulations were carried out with two fluorophore PSFs a certain distance apart. A series of images were generated in which the two PSFs were gradually moved closer together. The images were processed with ThunderSTORM and QuickPALM, both popular localization microscopy analysis algorithms. The localized positions were compared with the ground truth positions of the simulated fluorophores (see Supplementary Note 1 and Supplementary Fig. 1). Several factors likely to alter *r* were also examined in this manner, to determine the degree of their effect, and these are included in the Supplementary Material—the use of a multi emitter fitter is discussed in Supplementary Note 10 and illustrated in Supplementary Fig. 8, a higher NA objective is discussed in Supplementary Note 11 and shown in Supplementary Figs 9 and 10, and a much smaller pixel size is shown in Supplementary Fig. 11.

Both algorithms successfully found the correct positions of both fluorophores above ≈600 nm separation (*λ*=488 nm, NA=1.4). Below this, as the separation distance decreased, both algorithms showed a gradual bias towards the mean position of the two fluorophores. The distortion became larger at ≈400 nm, with QuickPALM rejecting half of the potential localizations, whereas ThunderSTORM developed a sharper bias towards the central position. Both algorithms fit the two fluorophores as one when separated by less than ≈400 nm. Therefore, for these analysis algorithms the exclusion region has a radius between 400 and 600 nm.

### DNA constructs for live-cell experiments

For the study of focal adhesions we used an mEOS2-vinculin construct^26^, which was kindly provided by Dr Clare Waterman (National Heart, Lung and Blood Institute, Bethesda, MD, USA). For the study of microtubules, we used the pQCXIP- tdEOS-*α*tubulin construct, which was a kind gift from Dr Michael Winding and Professor Vladimir Gelfand (Northwestern University, IL, USA).

### Cell lines and protein expression

To perform this study, HeLa human cervical carcinoma cell line (ATCC, Middelsex, UK) were grown in complete DMEM medium supplemented with 2 mM glutamine, 100 U ml^−1^ penicillin/streptomycin and 10% fetal bovine serum (all the cell media reagents from Sigma, Dorset, UK).

For transient expression of mEOS2-vinculin and mEOS2-tubulin, HeLa cells were transfected with Effectene Transfection Reagent (Qiagen) according to manufacturer's instructions. Briefly, cells were seeded in 24-well plates the day before transfection. Next day, cells were transfected and incubated overnight with the transfection mixture. Thereafter, transfected cells were harvested and re-seeded in 35 mm cell-imaging dishes and cultured for another 24 h. For live-cell imaging, medium was replaced with Optimem (Life Technologies, Paisley, UK) supplemented with 10% fetal bovine serum.

For imaging of fixed samples, Hela cells were seeded in 35 mm cell-imaging dishes, cultured overnight, thereafter washed twice with prewarmed PBS and fixed with 4% paraformaldehyde for 15 min at room temperature (RT). Fixed cells were permeabilized with methanol for 10 min at −20 °C and rehydrated in PBS. Cell samples were blocked with 5% BSA–PBS before incubation with the primary mouse anti-β-tubulin antibody (Sigma) in 3% BSA–PBS for 2 h at RT. Cells were washed five times with PBS, followed by a short block with 5% BSA–PBS. The primary antibody was detected with goat anti-mouse Alexa Flour 647 secondary antibody (Invitrogen) diluted in 3% BSA–PBS. After 1 h incubation at RT, the actin cytoskeleton was labelled with phalloidin Alexa Fluor 488 for 20 min at RT, cells were washed four times with PBS and stored at 4 °C in PBS containing 0.02% sodium azide, until imaging.

### Microscopy

Imaging was carried out on the Nikon N-STORM super-resolution system, with a × 100 1.49 NA Nikon objective. Images were recorded with an Andor Ixon DU897 Ultra camera (DU-897U-CS0-#BV), with a 16 μm pixel pitch and an exposure time of 10 ms. For samples expressing mEOS-2, the samples were continuously imaged using a low laser intensity at 405 nm, to switch fluorophores into the 561 nm absorbing state, and with a stronger intensity at 561 nm. For fixed-cell samples, the samples were illuminated using a 647 nm laser, with both the 647 nm laser and the 405 nm laser being used to control the activation density observed in the data. All experiments were carried out using near-total internal reflectance (TIRF) illumination.

### Data processing

For live-cell data, the sequences were edited to remove the beginning and end of each data set. The beginning was removed to exclude the brief period of extremely high-density data, which commonly occurs at the start of the acquisition, whereas the end was removed to prevent blurring due to movement of the live sample. To create an image sequence of a given length, frames were sampled uniformly at random from those remaining. This randomization prevents repeat localizations of a single emitters across several consecutive frames from artificially boosting the measured FRC of short image sequences.

The FRC was calculated using the Matlab implementation provided with the original paper. In every case, quoted FRC values are found by taking the average of 20 different random splits of the localization data.

The activation density was estimated by dividing the average number of localizations returned by the algorithm per frame by an estimate of the total area of the sample. This area estimate was found by hand annotating the QuickPALM reconstruction of each sample, based on the localizations returned by the maximum length image sequence available, using a reconstruction pixel of size 10 × 10 nm.

### Machine-learning classification of fit quality

Machine-learning methods train an algorithm to allow it to classify data. A user trains the algorithm by classifying features and the algorithm then optimizes a measure of classification performance on that training dataset. In this case, we select a small subset of localized positions from a data set to be classified by the user and we then classify the rest of the localizations in the data set using the trained algorithm. This allows the trained algorithm to be optimized for specific qualities of that data set.

Features are vectors consisting of a list of measurements of the localization under consideration, which are believed to be important. We build our feature vectors using the set of pixel values surrounding each reported localization. A window of 21 × 21 pixels around each localization is used. The localization lies in the centre of the middle pixel (bilinear interpolation is used to approximately infer the shifted pixel values) and the patch is rotated so the gradient across the centre pixel is aligned vertically (again using bilinear interpolation). The brightness of these shifted, rotated pixels is then scaled by multiplication with a Gaussian window, so as to reduce the apparent brightness of pixels farther from the centre point, as these are believed to be less informative of the fluorophore characteristics. This list of 441 scaled pixel values forms approximately one-third of the raw feature vector.

The remaining two-thirds of the raw feature vector is formed by performing the same shift, rotation and scaling on those pixel patches which fall in the frames immediately before and after the current frame. Thus, the feature vector contains information as to what that region of the sample looked like immediately before and afterwards. This adds a further 882 dimensions to the raw feature.

The final component of the raw feature is any further numeric information supplied by the localization algorithm. ThunderSTORM, for example, returns the fitted PSF width and fluorophore brightness, as well as its estimate of the background level and localization uncertainty, with each localization. This is a valuable information for the classifier, which can be used when learning the distinguishing characteristics of good and bad localizations. The additional information is usually quite low dimensional compared with the set of pixel values, typically resulting in a raw feature vector with a total size of <1,350 dimensions.

Two further manipulations convert the raw feature into a feature used for classification. First, each element is replaced with the natural log of its magnitude. Second, PCA is used to reduce the dimensionality of the feature from ∼1,350 to 20, where the components are based on the covariance matrix of the set of raw features observed for that particular acquisition.

These two manipulations are tied together. Dimensionality reduction is used as many of the elements of the raw feature vector are highly correlated—to aid storage of the data and speed up the training of the classifier, it is desirable to compress this. PCA was chosen as its runtime scales only linearly with the number of samples, while still capturing the majority of the variance of the observed data. This allows it to be run directly on each data set—thus, rather than choosing a fixed dimensionality reduction that is then applied to all data sets, a unique reduction is generated for each data set, to best capture its individual variability.

A weakness of PCA, however, is that finding the set of dimensions that best capture the covariance of the data is only optimal if the data are distributed as a multivariate Gaussian. Where this is not the case, performing PCA is equivalent to assuming that the data can be approximated as such a Gaussian. This is a strong assumption, particularly in microscopy data, where observed pixel intensities may vary by orders of magnitude between the beginning and end of an acquisition. Hence, we consider instead the natural log of the raw feature vectors values and perform PCA using these instead. This assumes the data is approximately log-normal distributed, which is a much milder assumption, and prevents a small number of extremely high/low-intensity patches dominating the form of the covariance matrix.

The end result of this is a 20-dimensional feature vector for each image patch. A set of ∼300 patches are selected for the user to hand label. Stratified sampling is used to ensure the set of patches to be labelled approximately spans the entire acquisition period, reducing the variance of the set, while avoiding the introduction of bias. The user labels each localization as either being ‘good' if they believe it corresponds to a single fluorophore, ‘background' if it is a random fluctuation due to Poissonian pixel noise or ‘too dense' if the fit appears to be inaccurate due to too high a density of fluorophores being activated. Using this, a random forest classifier is trained to infer the labels from the supplied feature vectors and is then applied to the remaining, unlabelled portion of the set.

The random forest classifier optimizes the classification performance on the training data set. Random forest classifiers are a standard machine-learning technique. They make use of an ensemble of tree classifiers, making decisions based on a majority vote. Each tree classifier is trained individually on the supplied training data. Tree classifiers are known to be unbiased and quick to train. However, they are high variance. Hence, they are well suited to ensemble methods, which reduce this variance by a factor of ∼1 over the number of trees trained, provided the trees are uncorrelated with one another. The correlation is reduced by forcing each tree to make a decision at each stage based on a small, random subset of the available dimensions and additionally using bootstrapping to train each one on a slightly different subset of the available training data. In this case, we used a forest of 300 individual trees.

This post localization classification scheme was developed in Matlab. An implementation will be made available for download.

### Data availability

Software is available as Supplementary Software 1, and updated versions of the software and the test data are available from coxphysics.com/lr. All other relevant data are available from the corresponding author.

## Additional information

**How to cite this article:** Fox-Roberts, P. *et al*. Local dimensionality determines imaging speed in localization microscopy. *Nat. Commun.* **8,** 13558 doi: 10.1038/ncomms13558 (2017).

**Publisher's note**: Springer Nature remains neutral with regard to jurisdictional claims in published maps and institutional affiliations.

## Supplementary Material

Supplementary Information Supplementary Figures 1-11, Supplementary Table 1, Supplementary Notes 1-11 and Supplementary References.

Supplementary Software Random Forest post processing method for localisation microscopy data.
