# Supplementary material for: Local dimensionality determines imaging speed in localization microscopy
Source: Nat Commun. 2017 Jan 12;8:13558. doi: 10.1038/ncomms13558 (PMC5241698; doi:10.1038/ncomms13558)
Supplement: Supplementary Information — Supplementary Figures 1-11, Supplementary Table 1, Supplementary Notes 1-11 and Supplementary References. [file ncomms13558-s1.pdf]

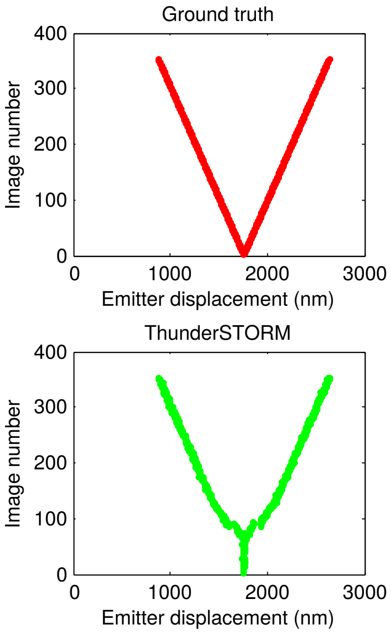

Supplementary Figure 1: Localisation results used to estimate radius of exclusion area. A synthetic image sequence was generated of a pair of emitters, initially coincident, which are gradually separated. Plots indicate the image number in the sequence against separation. Clockwise from the top left shows the ground truth emitter positions, the results using QuickPALM, and the results using ThunderSTORM.

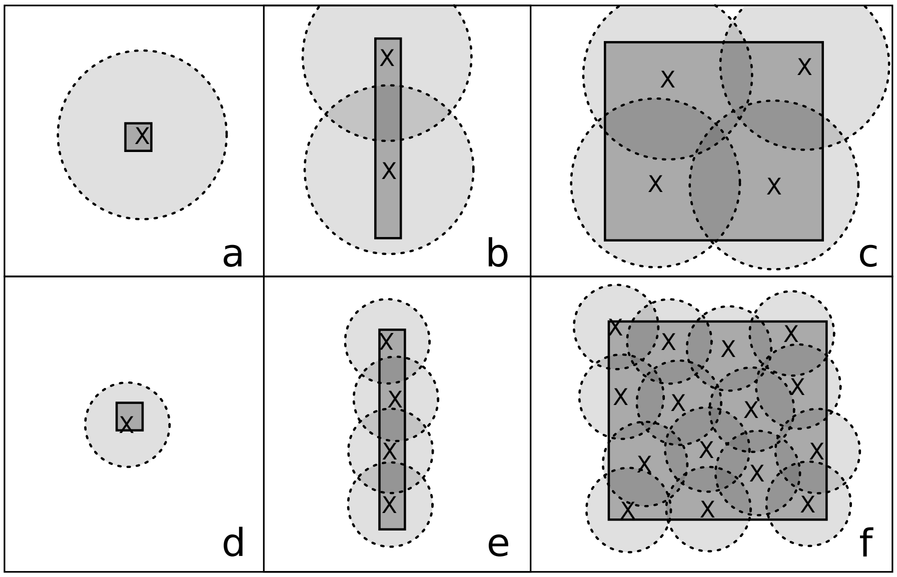

Supplementary Figure 2: A graphical representation of the effect of changing r on samples of varying dimensionality. Sub figures a-c show one, two and three dimensional samples respectively, each with active emitters. The samples are shown in dark gray, the emitter positions indicated with an 'X'. Additionally, the exclusion areas are shown as a light gray circle with a dotted border. Sub figures e-g show the effect of using a different analysis method, where the radius of the exclusion area has been halved. There is no improvement in the 0D case, a doubling of the acquisition speed for the 1D case, and close to a quadrupling of it for the 2D case.

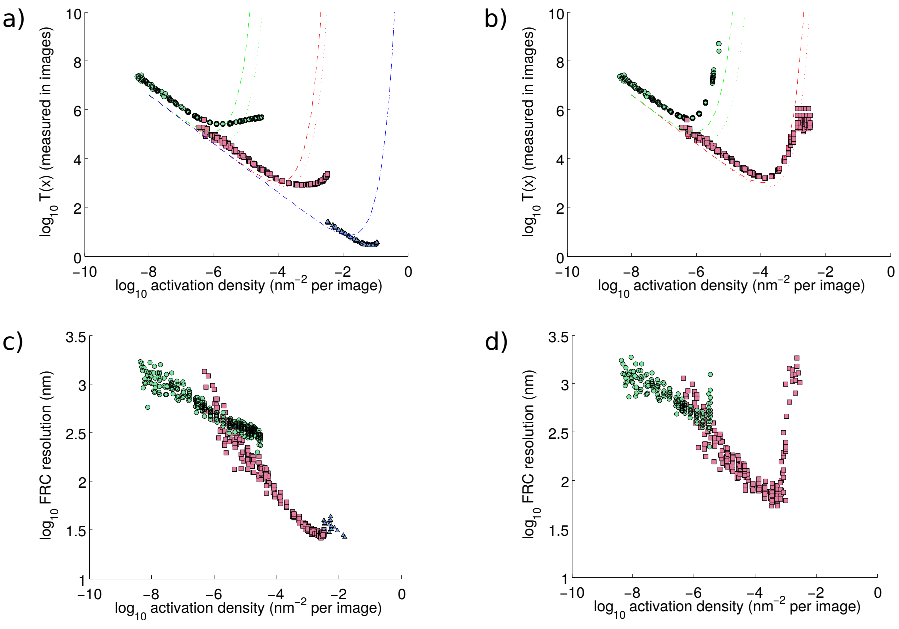

Supplementary Figure 3: Effect of dimensionality on expected acquisition time when using QuickPALM. a) shows the expected time to reach target quality Q against activation density assuming every localisation returned by QuickPALM is accurate. 0D data shown in blue triangles, 1D in red squares, and 2D in green circles. Theoretical predictions based on our upper and lower estimates of the exclusion area are shown with dashed and dotted lines respectively for each sample. b) shows the change in behaviour following classification for the 1D and 2D data. c) Shows the same data as a), but using FRC as the resolution measure. Similarly to b), d) shows the FRC measured for the 1D and 2D data following classification.

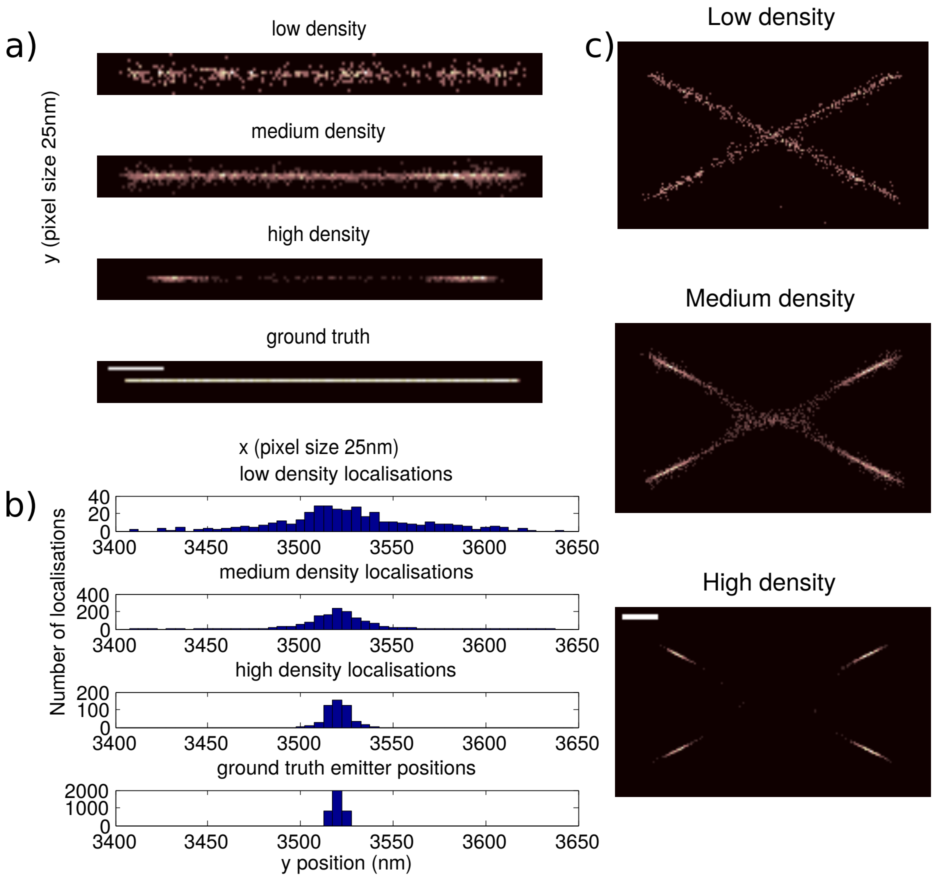

Supplementary Figure 4: Example artifical sharpening. High activation densities can change the apparent structure and lead to artificial sharpening. (a) Shows a filament-like computer generated structure. Ground truth is shown in the bottom image, while from the top down are three reconstructions at low, medium and high activations densities (15 μm^{-2}, 150 μm^{-2} and 1500 μm^{-2} respectively). The high density reconstruction shows severe distortion of the filament structure, only returning localizations at either end. This is because in the raw data the central area contains a higher degree of overlap. Note that despite the structure being incorrect, the localizations are on average much closer to the ground truth than the low density reconstruction. This is illustrated in (b), which shows a histogram plot of the distribution of localizations projected down onto the y-axis. So at high activation densities, false structure can be returned repeatably, and may also appear to match the underlying structure better than that returned by low density activations. (c) shows an example on a cross-shaped synthetic sample, evaluated at the same three densities as the filament-like structure in (a). Note that in the medium density image pinching of the central crossing can clearly be seen, and in the high density case the structure of the middle of the cross is absent, demonstrating that sharper images can have degraded resolution. All scale bars 500nm.

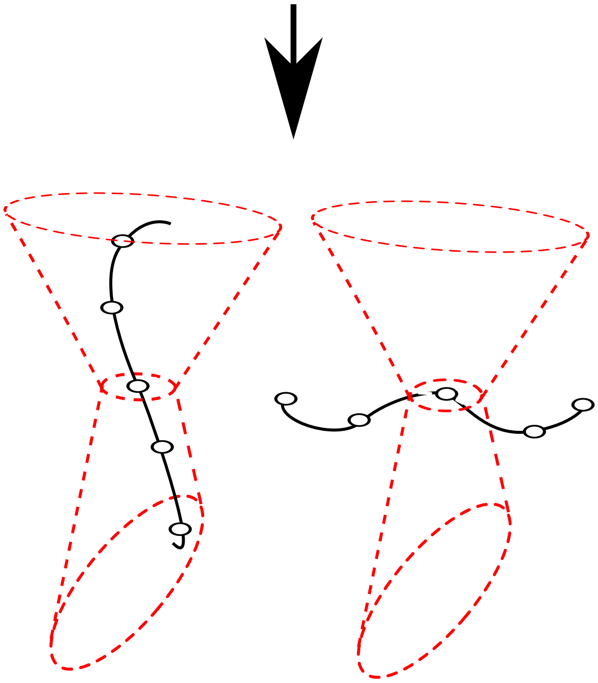

Supplementary Figure 5: An illustration of the importance of sample orientation when performing 3D localisation microscopy. Two filament like structures (so approximately 1D) tagged with fluorophores (indicated by white circles) suspended in a 3D sample are shown. The filaments are being imaged in the direction indicated by the large arrow, using the astigmatic method to infer 3D data (hence their point spread function changes with depth). In both cases, the middle fluorophore along the filament has activated. For the right hand filament, no other fluorophores along its length falls within the exclusion region of this active fluorophore, as indicated by the dotted red region. For the left hand filament, however, lies entirely within the exclusion region of the central fluorophore. Thus, the two have very different optimal activation densities, due solely to their orientation.

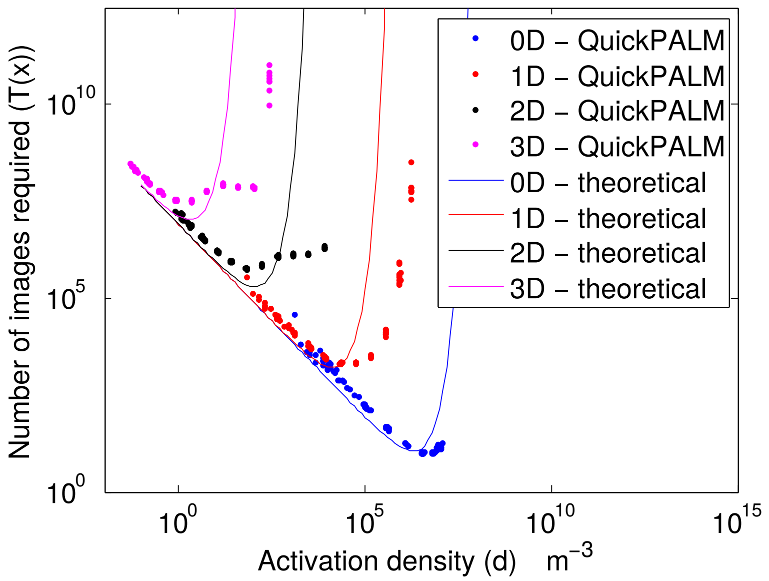

Supplementary Figure 6: Variation of imaging speed for three dimensional localisation microscopy. The graph shows a log-log plot of the estimated number of images required to generate a 3D reconstruction with $\mathcal{Q} = 8000,000$ molecules μm^{-3}, versus the active emitter density a. Results are shown for computer generated structures with dimensionality varying from $0 - 3$D (dotted) as well as the theoretically predicted results (continuous). Analysis performed using QuickPALM.

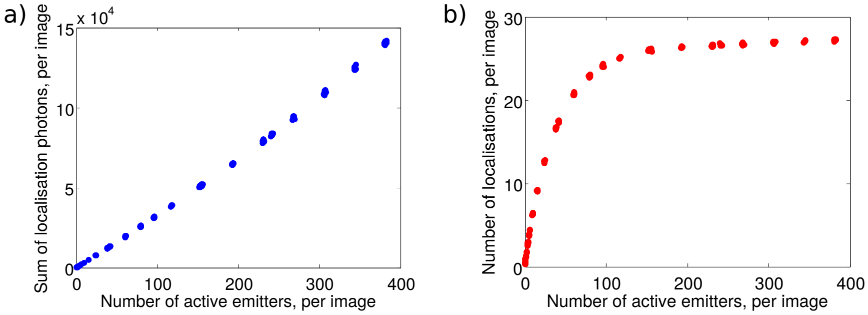

Supplementary Figure 7: An illustration of the use of average sum of localisation brightnesses as a proxy for the number of active fluorophores. test performed on the 2D simulated dataset. The horizontal axis in both figures shows the average number of active emitters, per image, for the structure. a) plots the sum of localisation brightnesses as estimated by ThunderSTORM against this - the relationship is very close to linear throughout, with a gradient equal to the estimated emitter brightness. b) shows the average number of localisations returned by ThunderSTORM on the same dataset. This relationship is linear up to no more than ≈ 5 activations, after which point ThunderSTORM begins to mistake groups of active emitters for a single emitter, under estimating the true number of fluorophores.

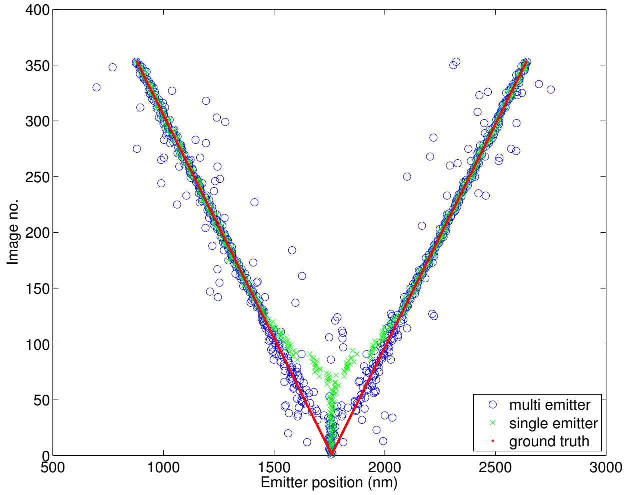

Supplementary Figure 8: Estimating the size of the exclusion region using the ThunderSTORM algorithm in multi-emitter fitting mode. Two emitters were simulated, and their separation gradually decreased. There is very little change in the separation distance at which the algorithm cannot reliably localise both. Note that the multi-emitter fitter can successfully localise both emitters at much smaller separations than the single emitter.

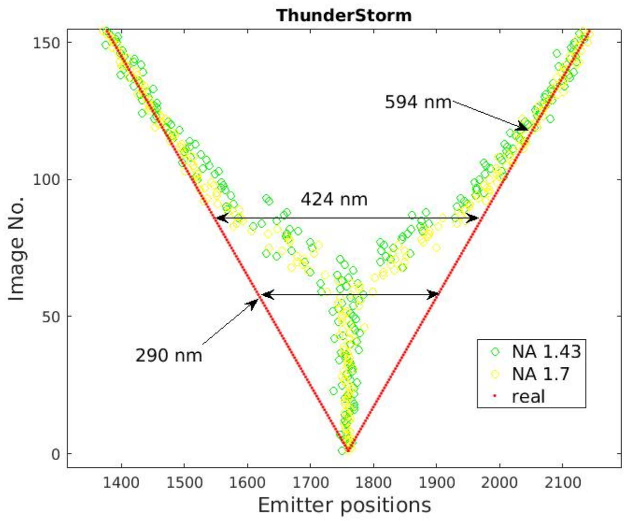

Supplementary Figure 9: Estimating the size of the exclusion region using the ThunderSTORM algorithm for two different objectives (NA 1.43 and 1.7). Two emitters were simulated, and their separation gradually decreased. There is very little change in the separation distance at which the algorithm cannot reliably localise both.

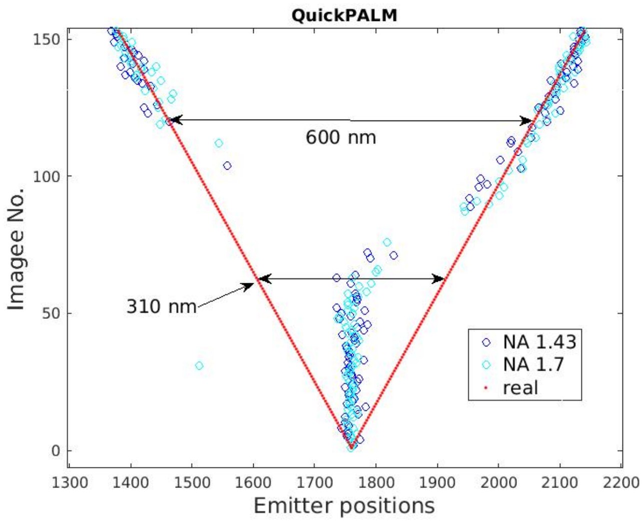

Supplementary Figure 10: Estimating the size of the exclusion region using the QuickPALM algorithm for two different objectives (NA 1.43 and 1.7). Two emitters were simulated, and their separation gradually decreased. There is very little change in the separation distance at which the algorithm cannot reliably localise both.

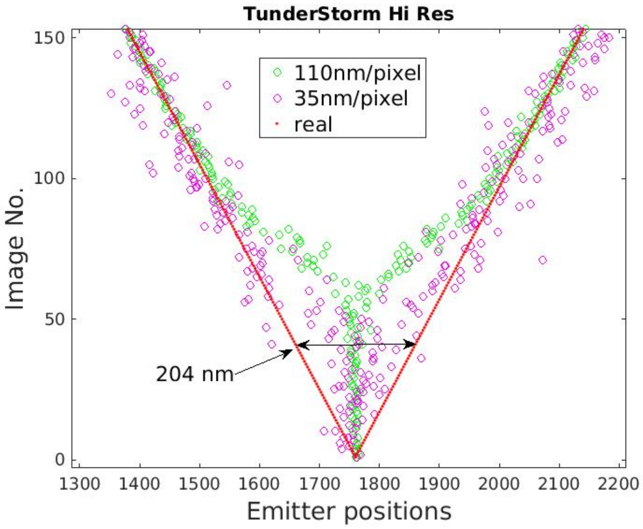

Supplementary Figure 11: Estimating the size of the exclusion region using the ThunderSTORM algorithm for two different pixel sizes (35nm and 110nm). Two emitters were simulated, and their separation gradually decreased. The 35nm width pixels, while resulting in noisier localisations, allow the two emitters to be successfully localised at a much shorter separation, and hence permit more rapid acquisition.

	$s_{t+1} =$ on	$s_{t+1} =$ off	$s_{t+1} =$ bleached
$s_t =$ on	$(0.975) * (1 - 0.005)$	$(1 - 0.975)$	$(0.975) * (0.005)$
$s_t =$ off	a	$1 - a$	0
$s_t =$ bleached	0	0	1

Supplementary Table 1: State transition probabilities for the Markov model simulation. Parameter a was varied across different runs, and a estimated from the average number of active emitters per image. States are updated every 0.5 ms.

1 Supplementary Note - Estimating exclusion region

Many of the provided results rely on estimating the size of the exclusion region of a algorithm - that is, the volume of image surrounding a single fluorophore activation which, if any other activations occur within it, will prevent that fluorophore being localised correctly.

This was estimated by generating a sequence of images in Matlab. Each image consisted of a uniform background level, and a pair of emitters modelled as having a Gaussian point spread function. The distance between the pair of emitters was initially set to zero and then linearly increased along the Y axis until their PSFs were well separated. These images were then analysed by the localisation algorithms. A plot of the coordinate of the returned localisations along the axis of separation against image number was created form this. If the algorithm were to perfectly locate each activation in each image this would resemble a V shape. In practise it resembles a distorted Y shape, due to the errors caused when the emitters become too close for the algorithm to resolve. This is shown in Supplementary Fig. 1.

Both QuickPALM and ThunderSTORM showed a sharp transition in behaviour as the emitters separation increased. This occurs for QuickPALM at a separation of ≈ 600 nm, below which it consistently failed to localise both emitters. ThunderSTORM successfully identified that there were two emitters to much lower degrees of separation; however, there was a step increase in the accuracy of its localisations at ≈ 400 nm separation, below which the emitter separation distance was underestimated to a marked degree. These two transitions in behaviour were taken to be the approximate distances above which the algorithms were able to reliably localise emitters, and hence the radius of the exclusion region.

2 Supplementary Note -Derivation of approximate $\mathcal{T}(x)$

We begin by defining the properties of and notation for the sample being analyzed.

- Define $\mathcal{S}$ as the region of the image which has been tagged, and we wish to make a localization microscopy representation of. We assume nothing outside the sample will be tagged.
- $|\mathcal{S}|$ is the area of $\mathcal{S}$

When imaging an actin strand, for example, $\mathcal{S}$ would be the area of the image which, were it not obscured by the point spread function, and ignoring pixelation effects, would contain the strand.

- Define a as the density of emitter activations on $\mathcal{S}$ per image. It will depend on the concentration of tagging medium used, laser power, etc. It has units of activations per unit area, per

image.

- Define N is the number of emitters which activate in region $\mathcal{S}$ in a single image. It is Poisson distributed with $\lambda = a|\mathcal{S}|1$, i.e.

$$P(N|a, \mathcal{S}) = \textbf{Poisson}(N; a|\mathcal{S}|1). \tag{1}$$

Note the multiplication by 1, to indicate that this the number of activations for a single image. For notational brevity this will be dropped for the remainder of the derivation.

Defining N as being Poisson distributed is an approximation. However we believe it is a good one. When a sample is tagged by a fixed number of emitters, and assuming random activations, then in any random image exposure the number of active emitters is binomially distributed. However, in accordance with the Poisson limit theorem, if the mean number of activations is held fixed and the number of tags allowed to tend towards infinity, then this distribution tends towards a Poisson distribution. As the number of tags is usually much greater than the number of active emitters at any given time, this approximation will hold in all cases we are considering. For completeness, however, we include the derivation for the case where the distribution over N is instead binomial later.

In a given image, each emitter which activates will have a position x. This will be two dimensional in 2D localisation microscopy, and three dimensional in 3D localisation microscopy. If N emitters activate, then, we have a set of activations $\{x_1 \ldots, x_N\}$ distributed according to $P(x_1, \ldots x_N|N)$.

- Assume the samples in x are independent and identically distributed (i.i.d) and do not vary with N. Then $P(x_1, \ldots x_N|N) = \prod_{i=1}^{N} p(x_i)$

Unfortunately, not all these activations will result in resolvable localizations - some will be too close together for the algorithm to correctly localize.

- Define r as the minimum separation between two active emitters for which the localization algorithm can still correctly localize them. The exact value will depend on the width of the point spread function, the depth of the sample, and the implementation details of the localization algorithm - but it will exist (as no localization method can separate arbitrarily closely spaced emitters). If $|x_i - x_j| \leq r$, then both localizations must be discarded.

- Define $\epsilon(x_i)$ as the "exclusion area" around an active emitter. It is the area where, if any other emitters activate within it, both emitters must be discarded. A point x is within $\epsilon(x_i)$ iff $|x_i - x| \leq r$.
- Define $\mathcal{S}_\epsilon(x)$ as $\mathcal{S} \cap \epsilon(x)$. This gives the region of the image within which an activation may occur while falling within $\epsilon(x)$.

Whether two or more samples fall within one another's exclusion area can be considered a random variable.

- Define the binary random variable b_i. b_i is equal to 1 iff all activations x_j where $j \neq i$ in the set of activations $\{x_i, \ldots x_N\}$ fall outside $\mathcal{S}_\epsilon(x_i)$.

The empirically reconstructed distribution of emitter positions D for a single image can then be expressed as

$$D(x) = \sum_{i=1}^{N} \delta(x, x_i) b_i. \tag{2}$$

This places a delta function at each observed x_i, provided no other activation occurs in its exclusion area. Note that we do not normalize this by $\sum_{i=1}^{N} b_i$. This is because when constructing a super resolution image, we do not normalize the integral each individual frame in the image sequence. Rather, we take the sum over all frames, and if desired normalize that. Also note that, implicitly, by considering a delta function centered at each point x_i in each frame, we are modelling a spatially varying *density* of localizations per unit area per image.

We define the target reconstruction quality $\mathcal{Q}$ as the density of correctly resolved samples in the final localization reconstruction (i.e. localisations per unit area of the reconstructed sample). The final localization reconstruction is found by summing the individual reconstruction densities D from each image. Finding the full probability distribution over the number of images required to achieve $\mathcal{Q}$ is complicated. However, with a sufficiently large number of frames in the sequence, and ignoring bleaching, the average contribution of each image will tend towards the expected value of the above equation. This can be written as

$$E\left[D(x)\right] = E_{P(N|a,\mathcal{S})}\left[E_{P(x_1,\ldots,x_N|N)}\left[E_{P(b_1,\ldots b_N|x_1,\ldots,x_N)}\left[\sum_{i=1}^{N} \delta(x_i, x) b_i\right]\right]\right] \tag{3}$$

where $E_{P(x)}[g(x)]$ represents the expected value of function $g(x)$ over distribution $P(x)$. As such we can find an approximate number of images required by finding $\mathcal{T}(x)$ such that

$$\mathcal{T}(x) E\left[D(x)\right] = \mathcal{Q} \tag{4}$$

i.e. the number of images necessary to achieve a reconstruction of quality $\mathcal{Q}$ assuming each one on average improves the reconstruction by $E\left[D(x)\right]$. Note that both T and D vary with x, and hence have been made functions of it. As such, different regions of the image will take a different amount of time to reach a particular target Q.

To calculate $T(x)$ we will simplify the expression for $E\left[D(x)\right]$. This involves taking the average over the random variables x, b and N.

Averaging with respect to b and x To average over x and b, we first make a change in order of summation,

$$E\left[D(x)\right] = E_{P(N|a,\mathcal{S})}\left[\sum_{i=1}^{N} E_{P(x_1,\ldots,x_N|N)}\left[E_{P(b_1,\ldots b_N|x_1,\ldots,x_N)}\left[\delta(x_i,x)b_i\right]\right]\right]. \tag{5}$$

This immediately allows a great number of variables to be marginalized out:

$$E\left[D(x)\right] = E_{P(N|a,\mathcal{S})}\left[\sum_{i=1}^{N} E_{P(x_i|N)}\left[E_{P(b_i|x_i)}\left[\delta(x_i,x)b_i\right]\right]\right]. \tag{6}$$

Writing out the expectations in full gives

$$E\left[D(x)\right] = \sum_{N=0}^{\infty} P(N|a,\mathcal{S}) \sum_{i=1}^{N} \int_{x_i\in\mathcal{A}} \sum_{b_i=0}^{1} p(x_i,b_i|N)\delta(x_i,x)b_i dx_i \tag{7}$$

which in turn can be evaluated using the sifting property of the delta function, and the binary nature of b_i, giving

$$E\left[D(x)\right] = \sum_{N=0}^{\infty} P(N|a,\mathcal{S}) \sum_{i=1}^{N} p(x_i = x, b_i = 1|N). \tag{8}$$

By symmetry, this simplifies to

$$E\left[D(x)\right] = \sum_{N=0}^{\infty} P(N|a,\mathcal{S}) N p(x_1 = x, b_1 = 1|N). \tag{9}$$

This leaves the quantity $p(x_1 = x, b_1 = 1|N)$ to evaluate. It can be expressed as

$$p(x_1 = x, b_1 = 1|N) = p(b_1 = 1|x_1 = x, N)p(x_1 = x|N) \tag{10}$$

The latter term, $p(x_1 = x|N)$, is simply the density function of the emitters across the sample, and is equal to $p(x)$. The former is slightly more involved to calculate

$$p(b_1 = 1|x_1 = x, N) = \\ \int_{x_2\in\mathcal{S}} \ldots \int_{x_N\in\mathcal{S}} p(b_1 = 1, x_2, \ldots x_N|x_1 = x, N)dx_2 \ldots dx_N. \tag{11}$$

Rearranging this, and taking advantage of the independent and identically distributed nature of x gives

$$p(b_1 = 1|x_1 = x, N) =$$
$$\int_{x_2 \in \mathcal{S}} \dots \int_{x_N \in \mathcal{S}} p(b_1 = 1|x_2, \dots x_N, x_1 = x, N) p(x_2) \dots p(x_N) dx_2 \dots dx_N. \quad (12)$$

Note that b_1 is equal to 1 if and only if all other samples fall outside $\mathcal{S}_\epsilon(x)$. We can change our integration region to reflect this:

$$p(b_1 = 1|x_1 = x, N) =$$
$$\int_{x_2 \in \mathcal{S} - \mathcal{S}_\epsilon(x)} \dots \int_{x_N \in \mathcal{S} - \mathcal{S}_\epsilon(x)} 1 \, p(x_2) \dots p(x_N) dx_2 \dots dx_N, \quad (13)$$

which is simply

$$p(b_1 = 1|x_1 = x, N) = \prod_{i=2}^{N} \int_{x_i \in \mathcal{S} - \mathcal{S}_\epsilon(x)} p(x_i) dx_i. \quad (14)$$

By symmetry, and using a dummy variable x' to perform the integration, this can be rearranged to

$$p(b_1 = 1|x_1 = x, N) = \left(\int_{x' \in \mathcal{S} - \mathcal{S}_\epsilon(x)} p(x') dx' \right)^{N-1} \quad (15)$$

which is equal to

$$p(b_1 = 1|x_1 = x, N) = \left(1 - \int_{x' \in \mathcal{S}_\epsilon(x)} p(x') dx' \right)^{N-1}. \quad (16)$$

Substitution this back into the expression for $E\left[D\right]$ gives the simplified expression

$$E\left[D\right] = \sum_{N=0}^{\infty} P(N|a, \mathcal{S}) N p(x) \left(1 - \int_{x' \in \mathcal{S}_\epsilon(x)} p(x') dx' \right)^{N-1}. \quad (17)$$

Averaging with respect to N We have now expressed $E\left[D\right]$ as

$$E\left[D(x)\right] = \sum_{N=0}^{\infty} P(N|a, \mathcal{S}) N p(x) \left(1 - \int_{x' \in \mathcal{S}_\epsilon(x)} p(x') dx' \right)^{N-1}. \quad (18)$$

We shall change notation slightly to simplify the expression we're working with, using the definition

$$f(x) \equiv \int_{x' \in \mathcal{S}_\epsilon(x)} p(x') dx' \quad (19)$$

giving

$$E\left[D(x)\right] = \sum_{N=0}^{\infty} P(N|a, \mathcal{S}) N p(x)(1 - f(x))^{N-1}. \tag{20}$$

Writing out the Poisson distribution for $P(N|a, \mathcal{S})$in full, we get

$$E\left[D(x)\right] = \sum_{N=0}^{\infty} \frac{(a|\mathcal{S}|)^N e^{-a|\mathcal{S}|}}{N!} N p(x)(1 - f(x))^{N-1}. \tag{21}$$

Noting the factor of N in the numerator, we can change the range of the summation to

$$E\left[D(x)\right] = \sum_{N=1}^{\infty} \frac{(a|\mathcal{S}|)^N e^{-a|\mathcal{S}|}}{N!} N p(x)(1 - f(x))^{N-1}, \tag{22}$$

and moving all factors which are not a function of N outside the summation gives

$$E\left[D(x)\right] = p(x) \sum_{N=1}^{\infty} \frac{(a|\mathcal{S}|)^N e^{-a|\mathcal{S}|}}{N!} N (1 - f(x))^{N-1}. \tag{23}$$

The N on the numerator cancels with the $N!$ on the denominator, giving

$$E\left[D(x)\right] = p(x) \sum_{N=1}^{\infty} \frac{(a|\mathcal{S}|)^N e^{-a|\mathcal{S}|}}{(N-1)!} (1 - f(x))^{N-1}. \tag{24}$$

Changing variables, by substituting $N' = N - 1$ gives

$$E\left[D(x)\right] = p(x) \sum_{N'=0}^{\infty} \frac{(a|\mathcal{S}|)^{N'+1} e^{-a|\mathcal{S}|}}{N'!} (1 - f(x))^{N'}. \tag{25}$$

Rearranging the equation yields

$$E\left[D(x)\right] = p(x) a|\mathcal{S}| \sum_{N'=0}^{\infty} \frac{\left(a|\mathcal{S}| - a|\mathcal{S}|f(x)\right)^{N'} e^{-d|\mathcal{S}|}}{N'!}, \tag{26}$$

and moving an extra factor of $e^{-a|\mathcal{S}|f(x)}$ out from the term inside the summation simplifies to

$$E\left[D(x)\right] = p(x) a|\mathcal{A}| e^{-a|\mathcal{S}|f(x)} \sum_{N'=0}^{\infty} \frac{\left(a|\mathcal{S}| - a|\mathcal{S}|f(x)\right)^{N'} e^{-(a|\mathcal{S}| - a|\mathcal{S}|f(x))}}{N'!}. \tag{27}$$

Note that the summation term simply corresponds to summing over a Poisson distribution with $\lambda = d|\mathcal{S}| - d|\mathcal{S}|f(x)$. As such, it sums to one, giving the result

$$E\left[D(x)\right] = p(x) a|\mathcal{S}| e^{-a|\mathcal{S}|f(x)} \tag{28}$$

i.e.

$$E\left[D(x)\right] = p(x) a|\mathcal{S}| e^{-a|\mathcal{S}| \int_{x' \in \mathcal{S}_\epsilon(x)} p(x') dx'} \tag{29}$$

This gives the expression for $\mathcal{T}(x)$ as

$$\mathcal{T}(x) = \frac{\mathcal{Q} e^{a|\mathcal{S}| \int_{x' \in \mathcal{S}_\epsilon(x)} p(x') dx'}}{p(x) d|\mathcal{S}|} \tag{30}$$

Uniform distribution of samples We have calculated an expression for the expected density of a reconstruction of a localization microscopy image. A particularly elegant result comes from considering the case where emitters are distributed uniformly throughout the sample. In that situation,

- $p(x) = 1/|\mathcal{S}|$
- $\int_{x' \in \mathcal{S}_\epsilon(x)} p(x') d'x = |\mathcal{S}_\epsilon(x)|/|\mathcal{S}|$

in which case the $E[D]$ is equal to $ae^{-a|\mathcal{S}_\epsilon(x)|}$.

- Some simple calculus shows that the value a^* where this density is maximized at point x is equal to $1/|\mathcal{S}_\epsilon(x)|$
- At a^*, the density is equal to $(e|\mathcal{S}_\epsilon(x)|)^{-1}$

This gives an optimum value for $\mathcal{T}(x)$ of

$$\mathcal{T}^*(x) = \mathcal{Q}e|\mathcal{S}_\epsilon(x)|. \tag{31}$$

This can now be directly linked to the dimensionality of the sample by noting the dependence between the size of $\mathcal{S}_\epsilon(x)$ and the radius r of $\epsilon(x)$:

- A '0D' sample is one which is smaller than r all directions. $|\mathcal{S}_\epsilon(x)| = |\mathcal{S}|$
- A '1D' sample is one which is smaller than r in one direction, but much larger than it in the other. Assuming a width of w, and approximating the intersection as a rectangle, then $|\mathcal{S}_\epsilon(x)| \approx 2wr$
- A '2D' sample is one which is larger than r in both directions. It is therefore simply a circle, with $|\mathcal{S}_\epsilon(x)| \approx \pi r^2$
- And for the case of 3D localization, a '3D' sample is one which is larger than r in all directions, including axially. It is therefore approximately a sphere, with $|\mathcal{S}_\epsilon(x)| \approx \frac{4}{3}\pi r^3$

This can be written in a general form as

$$|\mathcal{S}_\epsilon(x)| \approx c(x) r^{\mathcal{D}} \tag{32}$$

where $c(x)$ is some position dependent constant, as illustrated in Supplementary Fig. 1. This gives

$$\mathcal{T}^*(x) \approx \mathcal{Q}ec(x)r^{\mathcal{D}}. \tag{33}$$

Note that $c(x)$ is only approximately constant with respect to r - sufficiently large variations in r will alter it. In particular near the edges of samples there may be ranges of values of r which cause sharp, rapid changes in $c(x)$.

Solving for alternative distributions over N In the above we have assumed that the probability distribution over N takes the form of a Poisson distribution. In reality, this is not the case - in particular, the Poisson distribution allows a small (but non-zero) probability of an arbitrarily high number of emitters activation. Whereas in practice, this number will always be capped by the maximum number of emitters which have been attached to the sample. In the case where this number is high, the Poisson distribution is a good approximation. However, where the number is more moderate, it is better to use a binomial distribution.

To do this, we replace $P(N|a, \mathcal{S})$ with $P(N|\rho, N_m)$. In this parameterization, N_m is the maximum number of emitters available to activate, and ρ is the probability that any given emitter will do so. $E[D]$ then becomes

$$E[D] = \sum_{N=0}^{N_m} P(N|\rho, N_m) N p(x) \left(1 - \int_{x' \in \mathcal{S}_\epsilon(x)} p(x') dx'\right)^{N-1}. \tag{34}$$

The derivation now follows similarly. We again change notation slightly to simplify the expression we're working with, using the following definition,

$$f(x) \equiv \int_{x' \in \mathcal{S}_\epsilon(x)} p(x') dx' \tag{35}$$

giving

$$E[D] = \sum_{N=0}^{N_m} P(N|\rho, N_m) N p(x) (1 - f(x))^{N-1}. \tag{36}$$

Writing out the binomial distribution for $P(N|\rho, N_m)$ in full, we get

$$E[D] = \sum_{N=0}^{N_m} \frac{N_m!}{N!(N_m - N)!} \rho^N (1-\rho)^{N_m - N} N p(x) (1 - f(x))^{N-1}. \tag{37}$$

Noting the factor of N in the numerator, we can change the range of the summation to

$$E[D] = \sum_{N=1}^{N_m} \frac{N_m!}{N!(N_m - N)!} \rho^N (1-\rho)^{N_m - N} N p(x) (1 - f(x))^{N-1}. \tag{38}$$

The N on the numerator cancels with the $N!$ on the denominator, giving

$$E[D] = \sum_{N=1}^{N_m} \frac{N_m!}{(N-1)!(N_m-N)!} \rho^N (1-\rho)^{N_m-N} p(x)(1-f(x))^{N-1}. \tag{39}$$

Changing variables, by substituting $N' = N-1$ and similarly $N'_m = N_m - 1$ gives

$$E[D] = \sum_{N'=0}^{N'_m} N_m \frac{N'_m!}{N'!(N'_m-N')!} \rho^{N'+1} (1-\rho)^{N'_m-N'} p(x)(1-f(x))^{N'}. \tag{40}$$

Moving all possible terms outside the summation simplifies the expression to

$$E[D] = p(x) N_m \rho \sum_{N'=0}^{N'_m} \frac{N'_m!}{N'!(N'_m-N')!} \rho^{N'} (1-\rho)^{N'_m-N'} (1-f(x))^{N'}, \tag{41}$$

and gathering together terms with a common exponent yields

$$E[D] = p(x) N_m \rho \sum_{N'=0}^{N'_m} \frac{N'_m!}{N'!(N'_m-N')!} (\rho(1-f(x)))^{N'} (1-\rho)^{N'_m-N'}. \tag{42}$$

We now introduce a new term of the form $(\rho(1-f(x))+1-\rho)^{N'_m}$ inside the summation, and its inverse outside, giving

$$\begin{aligned} E[D] = \quad & p(x) N_m \rho (\rho(1-f(x))+1-\rho)^{N_m-1} \\ & \times \sum_{N'=0}^{N'_m} \frac{N'_m!}{N'!(N'_m-N')!} \frac{(\rho(1-f(x)))^{N'}(1-\rho)^{N'_m-N'}}{(\rho(1-f(x))+1-\rho)^{N'_m}}. \end{aligned} \tag{43}$$

Defining $c = \rho(1-f(x))/((\rho(1-f(x))+1-\rho))$ this now simplifies to

$$\begin{aligned} E[D] = \quad & p(x) N_m \rho (\rho(1-f(x))+1-\rho)^{N_m-1} \\ & \times \sum_{N'=0}^{N'_m} \frac{N'_m!}{N'!(N'_m-N')!} c^{N'} (1-c)^{N'_M-N'}, \end{aligned} \tag{44}$$

which clearly results in the summation being equal to 1, simplifying the expression to

$$E[D] = p(x) N_m \rho (\rho(1-f(x))+1-\rho)^{N_m-1}. \tag{45}$$

This can be further simplified to

$$E[D] = p(x) N_m \rho (1-\rho f(x))^{N_m-1}, \tag{46}$$

and substituting the known value of $f(x)$ finally gives

$$E[D] = p(x) N_m \rho \left(1 - \rho \int_{x' \in \mathcal{S}_\epsilon(x)} p(x') dx'\right)^{N_m-1}. \tag{47}$$

This is functionally quite similar to the form of solution found when N was Poisson distributed. Indeed, if we fix the product ρN_m to a constant value, and left $N_m \to \infty$, then this tends exactly to the Poisson solution.

The activation probability ρ which maximises $E[D]$ can be found in a straightforward manner by differentiating this equation and setting the result to 0. This gives $\rho = 1/N_m$. Note that we would usually expect N_m to grow approximately linearly with $|\mathcal{S}|$, assuming all parts of the sample are roughly equally likely to be tagged.

Solving when N_m can be reduced by bleaching For the case where N_m is finite, and each fluorophore has an activation probability ρ, we have shown that expected value of D can be expressed as

$$E[D] = \sum_{N=0}^{N_m} P(N|\rho, N_m) N p(x) \left(1 - \int_{x' \in \mathcal{S}_\epsilon(x)} p(x') dx'\right)^{N-1}, \tag{48}$$

where $P(N|\rho, N_m)$ is a binomial distribution.

Note that when we calculate this expected value, we assume that each image features N activations from a constant sized pool of N_m available fluorophores. A more physically realistic model for many situations would be to assume that this pool of available fluorophores is depleted over time. This models fluorophores entering a bleached state, from which they no longer activate.

We approximate this as follows. Suppose, as before, that there is an initial pool of N_m available fluorophores available to activate. We shall acquire T images in our sequence. In a given image, each available fluorophore activates with probability ρ, as before. Additionally, though, in each image each available fluorophore may also bleach, with probability ρ_b. When bleached, a fluorophore may no longer activate, reducing the number of available fluorophores in all subsequent images. As such, instead of being able to consider a simple, constant distribution for N at each given time period, we have to consider the distribution over $P(N(1), N(2), \ldots, N(T)|\rho, \rho_b, N_m)$, where $N(1)$ is the number of active fluorophores in image 1, $N(2)$ in image 2, and so on.

Choosing an objective function to optimise for such a situation is non-trivial. One might justifiably attempt to maximise the number of successful localisations over a given time period; minimise the acquisition time subject to the constraint that a certain quality of reconstruction is achieved with a given probability; maximise the expected number of good localisations per image; and so on. Exploring this space of optimisation objectives is outside the scope of a single paper. Instead, we shall examine the objective function we consider most closely comparable to that we have used so far, which we define as

$$E[D_T] = E\left[\sum_{t=1}^{T} N(t) p(x) \left(1 - \int_{x' \in \mathcal{S}_\epsilon(x)} p(x') dx'\right)^{N(t)-1}\right], \tag{49}$$

where the right hand expectations is taken with respect to $P(N(1), N(2), \ldots, N(T)|\rho, \rho_b, N_m)$.

Note the summation term $\sum_{t=1}^{T}$ within the expectation - we use this as we are interested in the resulting summed distribution over all T images. Earlier, as the distribution of N was considered constant over time, this could be done simply be multiplying $E\left[D\right]$ by T as the final step, but now this is no longer possible.

Due to the linearity of expectations, we can rearrange the equation as follows

$$E\left[D_T\right] = \sum_{t=1}^{T} E\left[N(t)p(x)\left(1 - \int_{x' \in \mathcal{S}_\epsilon(x)} p(x')dx'\right)^{N(t)-1}\right]. \tag{50}$$

This has a much nicer form, as it allows the expectation to be simplified greatly by marginalising over all $N(t')$ for $t' \neq t$, giving the expression

$$E\left[D_T\right] = \sum_{t=1}^{T} \sum_{N(t)=0}^{N_m} P(N(t)|\rho, \rho_b, N_m)N(t)p(x)\left(1 - \int_{x' \in \mathcal{S}_\epsilon(x)} p(x')dx'\right)^{N(t)-1}. \tag{51}$$

Now we have to find $P(N(t)|\rho, \rho_b, N_m)$. This is simplified by nothing that, given a constant bleaching probability ρ_b, the number of available unbleached fluorophores after each time period, given the number available before, is itself binomially distributed. Using the notation that $B(x; n, p)$ is a binomial distribution over x with n trials and a chance of success equal to p, and the identity that if $P(X) = B(X; M, q)$ and $P(Y|X) = B(Y; X, p)$ then $P(Y) = B(Y; M, pq)$, it can be shown that the probability distribution over the number of emitters which activate at time t is

$$P(N(t)|\rho, \rho_b, N_m) = B(N(t); N_m, \rho(1 - \rho_b)^{t-1}). \tag{52}$$

This has a pleasant interpretation. The maximum number of emitters available at any given time period is N_m, as there is a non-zero (but usually small) probability that no emitters will have bleached at all previously. The total pool of potential activatable emitters at any given time is therefore fixed at N_m. In order for any emitter to activate, it must first not have bleached at any given time period prior to t (which occurs with probability $(1 - \rho_b)^{t-1}$) and must then activate during the current time period (with probability ρ), giving an overall activation probability of $\rho(1 - \rho_b)^{t-1}$.

Note, however, that the distribution for N where N_m is constant was also a binomial distribution with N_m trials. The only difference is that ρ has been replaced with $\rho(1 - \rho_b)^{t-1}$, so there is now a time dependent activation probability due to bleaching. As such, we can re-use the earlier result to simplify the terms within the summation over t and marginalise out $N(t)$ to give

$$E\left[D_T\right] = \sum_{t=1}^{T} p(x)N_m\rho(1 - \rho_b)^{t-1}\left(1 - \rho(1 - \rho_b)^{t-1} \int_{x' \in \mathcal{S}_\epsilon(x)} p(x')dx'\right)^{N_m-1}. \tag{53}$$

This seems intuitively reasonable. If $\rho_b = 0$ then we recover the case where N_m is constant, and $E[D_T] = TE[D]$. For the case where $\rho_b > 0$, as t becomes large the term $\rho(1-\rho_b)^{t-1}\int_{x'\in\mathcal{S}_\epsilon(x)} p(x')dx'$ tends towards 0, and so the effect of variations in size over the sample of the exclusion region diminishes; or in other words, it is early in the acquisition, where few fluorophores have bleached, that the effect of varying local fluorophore density of the sample is most pronounced.

Unfortunately, at this point, the expression becomes significantly less tractable. In particular, in order to specify an appropriate laser power to stimulate the fluorophores with, it is necessary to know its effect on both ρ and ρ_b. These will likely be correlated, but still may vary significantly between different kinds of fluorophores. Even if an analytic expression can be found for the relationship, the optimal laser power will be a nonlinear function of T, and most likely it will not have a closed form solution. An approximate solution will therefore have to be found via numerical methods.

3 Supplementary Note - Determining speedup due to change of r

Estimating $c(x)$ is challenging, as this depends on properties of the sample which may be only very roughly known. This is particularly true for low dimensional samples of unknown size - for example, if $r = 0.4\ \mu$m, then both a $0.01 \times 0.01\ \mu$m^2 and a $0.06 \times 0.06\ \mu$m^2 sample will have the same dimensionality (0D) but very different values of $c(x)$, resulting in non-trivial variations in $\mathcal{T}(x)$. It also has a strong effect near the edge of higher dimensional samples. However, overall we still have a good order of magnitude estimate, as the scaling with $\mathcal{D}$ will likely dominate all but exceptionally large inaccuracies in $c(x)$.

Even if $c(x)$ cannot be estimated, we can approximate the expected speedup caused by using an algorithm with a smaller $\epsilon(x)$. If r is reduced by a factor of k where $k \leq 1$, then the new number of images required, $\mathcal{T}_{\textbf{new}}(x)$, obeys the relationship

$$\frac{\mathcal{T}_{\textbf{new}}(x)}{\mathcal{T}(x)} \approx \frac{\mathcal{Q}c(x)(kr)^{\mathcal{D}}e}{\mathcal{Q}c(x)r^{\mathcal{D}}e} = k^{\mathcal{D}} \tag{54}$$

and so the number of images required is reduced by a factor of $k^{\mathcal{D}}$, improving with sample dimensionality.

In the main text, we have proposed that the shortest average acquisition time in terms of the number of images required is approximately equal to

$$\mathcal{Q}c(x)r^{\mathcal{D}}e. \tag{55}$$

In general this is difficult to evaluate, as it requires estimating the term $c(x)$. However, even if this cannot be done, we can approximate the expected speedup caused by using an algorithm with a

smaller $\epsilon(x)$. If r is reduced by a factor of k where $k \leq 1$, then the new number of images required, $\mathcal{T}_{\mathbf{new}}(x)$, obeys the relationship

$$\frac{\mathcal{T}_{\mathbf{new}}(x)}{\mathcal{T}(x)} \approx \frac{\mathcal{Q}c(x)(kr)^{\mathcal{D}}e}{\mathcal{Q}c(x)r^{\mathcal{D}}e} = k^{\mathcal{D}} \tag{56}$$

and so the number of images required is reduced by a factor of $k^{\mathcal{D}}$, improving with sample dimensionality. This is illustrated in Supplementary Fig. 2.

4 Supplementary Note - Artificial sharpening

To demonstrate this, we simulated filament type structures, which are common in biological samples (shown in Figure 4a). We generated three datasets, one at a low activation density ($15\ \mu$m^{-2}), one at an intermediate density ($150\ \mu$m^{-2}), and one at a high density($1500\ \mu$m^{-2}). The reconstruction of high density data shows obvious artefacts, with almost all the localisations being concentrated at either end of the filament. This is because the point spread functions of the emitting fluorophores in the middle of the filament were overlapping too much to be successfully localised. However, if you consider the distribution of localisations across the filament it appears substantially narrower than in the reconstruction from low density data (shown in Figure 4b. The reconstruction from intermediate density data shows some sharpening, without the obvious artefacts of the reconstruction from high density data.

This artificial sharpening occurs because if an erroneous localisation is carried out on, for example, two fluorophores that lie on either side of the filament, the fitted central position will lie between the position of the two fluorophores, closer to the central line of the structure than a fit to either fluorophore alone would have provided, with the variance reduced by a factor of approximately $1/2$. A similar result will hold for larger groups of fluorophores which activate sufficiently closely together that they are mistaken for one. The end result is that the returned localization estimations are distributed along the middle of the filament with lower variance than when fitting to a correctly identified single emitter, despite the fitting process being severely distorted by the large amount of overlap. An attempt to quantify how well the algorithm has performed by examining how close each localization is to the ground truth structure will conclude that the high activation density produces good results - and by its own metric, would do so correctly. However, if we examine a structure such as a cross (see Figure 4c) it can be seen that the artificial sharpening leads to pinching in the centre of the cross, or in the high density case, to the centre of the cross being absent. Thus, although the reconstructed image is sharper at high densities, it has lower resolution. This illustrates the care which must be taken when assessing the quality of a localization microscopy reconstruction, as even a set of parameters whose reconstruction correlates highly with the underlying structure maybe fitting incorrectly, with the potential to fail undetectably on other samples or in other regions of the image.

5 Supplementary Note - QuickPALM results

The post-processing classifier is intended to be localisation algorithm agnostic - provided the algorithm returns a list of localisation positions and the image number in the sequence the corresponding activation was observed in, it ought to be usable. The intent behind this is to allow the practitioner to make use of whatever localisation method is most suitable given the behaviour of their data, available computing resources, time constraints, and so on. To test this we analysed the synthetic datasets using QuickPALM [19], a popular and fast localisation scheme which is available as an ImageJ plugin.

The training method followed exactly the same process as that when using ThunderSTORM. The data is analysed using QuickPALM, then for each localisation a feature vector is built up using the surrounding pixel data as well as the additional information about that localisation returned by the QuickPALM algorithm, which is then compressed to 20 dimensions using PCA. The user then labels a small subset of localisations, judging their quality by eye. Using this labelled data a Random Forest Classifier is trained, which is then used to classify the remaining, unlabelled localisations. The results of this are shown in Supplementary Fig. 3.

6 Supplementary Note - 3D results

As noted in the main text, the same basic analysis of the effect of sample dimensionality applies to 3D localisation microscopy as 2D. There are, however, two important caveats.

Firstly, to infer depth, the point spread function of the fluorophores generally changes depending on its distance in the axial direction from the camera, such as by inducing an astigmatism [30,31] or a double helix [32] (though some other methods avoid this [33]). Changing the point spread function in this way increases $\mathcal{S}_\epsilon(x)$ in the image plane.

Secondly, if the sample itself is intrinsically 3D at the scale of the point spread function, as opposed to being a lower dimensional structure distributed over a three dimensional space, then this increases $|\mathcal{S}_\epsilon(x)|$ by a factor proportional to the sample's depth. This occurs because while we refer to the portion of the sample surrounding a localisable active fluorophore as the exclusion area, in reality it is a volume. When performing 2D localization the depth of this volume can be minimised using methods such as TIRF. When performing 3D localization this is not possible. As the recorded image is projected onto the camera sensor, the point spread function of activations with significant separation in the axial direction can still overlap if they are not well separated in the direction of the image plane - this results in an exclusion region which is elongated in the Z direction compared to X and Y. As such, the orientation of the sample,as well as its dimensionality, will have a significant effect on the necessary acquisition time. A filament like structure, for example, parallel to the image plane, can be imaged far more rapidly than one perpendicular to it, as the exclusion areas

are very different; similarly, there is a significant difference between a sample which is locally 2D but which has structure in the third dimension (a plane with folds in it, for example), compared to a sample which is truly 3D. See Supplementary Fig. 5 for an illustration of this.

The effect of dimensionality on 3D localization microscopy was tested using synthetic data, with depth estimated using the astigmatic method [30,31] . Four simple synthetic structures were simulated, each containing a uniform distribution of fluorophores, using the same blinking and bleaching dynamics as the simulated 2D structures. The astigmatism was simulated by scaling the point spread function linearly with depth, along either the horizontal or vertical axis depending on whether the active fluorophore fell above or below the focal plane:

- three $8 \times 8 \times 8$ nm^3 '0D' points, well separated in x and y, and with z depths -400 nm, 0 nm, and 400 nm. Tagging density 5 nm^{-3}.
- an $8 \times 3520 \times 8$ nm^3 '1D' line, with a z depth that varies linearly in x from -400 nm to 400 nm. Tagging density 0.05 nm^{-3}.
- a $3520 \times 3520 \times 8$ nm^3 '2D' plane, with a z depth that varies linearly in x from -400 nm to 400 nm. Tagging density 0.05 nm^{-3}.
- and a $3520 \times 3520 \times 440$ nm^3 '3D' cuboid. Tagging density 0.0005 nm^{-3}.

These were localized in three dimensions using 3D QuickPALM.

We found that the minimum separation between two reported localizations was ≈ 440 nm. Where it was necessary to estimate $\mathcal{S}_\epsilon(x)$, the following assumptions were made:

- For the 0D sample, it was assumed that the sample $\mathcal{S}$ fell entirely within $\epsilon(x)$ for all readings. Therefore $|\mathcal{S}_\epsilon(x)| = 8 \times 8 \times 8$ nm^3.
- For the 1D sample, it was assumed that $\epsilon(x)$ is approximately a sphere, of radius 440 nm. Its intersection with an 8×8 nm wide filament was approximated by a cuboid, with width equal to the filament and length equal to the diameter of the circle, ignoring edge effects. This gives $|\mathcal{S}_\epsilon(x)| = 8 \times 8 \times 880$ nm^3.
- For the 2D sample, it was assumed that $\mathcal{S}_\epsilon(x)$ is an approximately circular slice, with radius 440 nm. Ignoring edge effects, this gives $|\mathcal{S}_\epsilon(x)| = 8 \times \pi 440^2$ nm^3.
- For the 3D sample, it was assumed that $\mathcal{S}_\epsilon(x)$ is approximately cylindrical, with radius 440 nm and depth equal to the sample. Ignoring edge effects, this gives $|\mathcal{S}_\epsilon(x)| = 440 \times \pi 440^2$ nm^3.

To calculate $\mathcal{T}(x)$, it is necessary to set a target reconstruction density $\mathcal{Q}$. Results are shown for $\mathcal{Q} = 1/125$ nm^{-3} (one localization per $5 \times 5 \times 5$ nm^3 region). It is also necessary to estimate a. This was performed by averaging the number of active emitters per image across all images in the sequence, and dividing this by the tagged area/volume of the sample.

A similar scaling behaviour to that of the 2D data was observed, with the addition of another dimension adding more than another order of magnitude on to the imaging time, as shown in Supplementary Fig. 6.

Note that at this time the random forest classification method has not been tested for use in 3D localisation using the astigmatic method, as it relies on regions which are too dense being identifiable by eye. This is significantly more challenging when using a non-isotropic point spread function, as it introduces significant ambiguity between a region which is too dense and a single fluorophore at a particular depth. As such, we made use of the fact that this is computer simulated data, and hence the ground truth fluorophore positions were available. A quick estimate of the number of correct localisations was made using a Bayesian classifier built on this data, as discussed in Supplementary Method 7. This finds an estimate of the probability that each returned localisation corresponds to a single fluorophore given its position, and uses this as a weight to find an estimate of the average number of accurate localisations per image. We did not use this method more widely in the analysis as it tends to slightly over estimate the number of accurate localisations (as even a localisation in a very dense region will likely be classified as having a non-zero probability of being accurate). While this is not noticeable for low activation densities, at high activation densities where the predicted number of localisations decreases exponentially it tends to systematically under estimate the necessary acquisition time.

7 Supplementary Note - Bayesian classification of good/bad localisations

Quantifying the quality of a localization microscopy analysis is a challenging problem. A major reason for this is that certain errors are qualitatively different. The sort of errors encountered when analysing an image include:

- A reported localization, when no emitter activated (a false positive)
- No reported localization, when an emitter activated (a false negative)
- A reported localization a significant distance from an activated emitter (a true positive imprecisely localized)
- A reported localization in the vicinity of two or more closely spaced active emitters (multiple emitters mistaken for one, often imprecisely localized)

For our tests we wish to evaluate the total number of ‘good’ localizations the algorithm has found, where a good localization is one in which a single emitter has been correctly identified, and its position reported with the expected level of accuracy given the photon count, signal to noise ratio, etc (this second requirement is necessary to eliminate spurious localizations due to background noise).

As we are using synthetic data we know the ground truth locations and brightness of each activated emitter in each image, as well as the background level. This allows us to build a statistical model to approximately evaluate the probability that in image i a localization l corresponds to emitter em, $P(em|l,i)$ (the exact process by which this model is built is described later).

Using the model for $P(em|l,i)$, and additionally defining

- N_I as the number of images
- $L(i)$ as the set of reported localizations in image i
- $E(i)$ as the set of emitters which were active in image i

we can evaluate a score S for how well the reported localizations match the ground truth emitter positions. This is given by

$$S = \sum_{i=1}^{N_I} \sum_{l \in L(i)} \max_{em \in E(i)} P(em|l,i). \tag{57}$$

This counts the number of reported localizations, but weighs each one by it maximum probability assignment to the ground truth activations. Comparing it to the errors described above, we find the following:

- A reported localization, when no emitter activated, contributes 0 to S
- No reported localization, when an emitter activated, contributes 0 to S
- A reported localization a significant distance from an activated emitter, contributes between 0 and 1 to S depending on the size of position error
- A reported localization in the vicinity of two or more closely spaced active emitters contributes between 0 and 1 to S, depending on the position error from the closest active emitter, and decreasing as the number of nearby emitters increases

We argue that this closely approximates a count of the number of 'good' localizations, i.e. those which correspond to a single active emitter which has been well localized.

To evaluate S we must specify the exact form of the model for $P(em|l,i)$. We assume localization errors are normally distributed and unbiased, and detection probability increases approximately linearly with emitter brightness (measured in photons), allowing us to define the joint distribution $P(em,l|i)$ as

$$P(em,l|i) = w_{em}\mathcal{N}(l|\mu_{em},\sigma_{em}) \tag{58}$$

where μ_{em} is the position of active emitter em, the standard deviation σ_{em} calculated using the square root of formula for localization variance given in [29], and w_{em} is proportional to the number of photons emitted by this emitter in image i. Evaluating this can be thought of as finding the probability that the localization is a true positive, and was generated by emitter em.

There is also a possibility that the reported localization l is due to a random background fluctuation b (and so is a false positive). This is modelled as

$$P(b,l|i) = w_b\frac{1}{U} \tag{59}$$

where $1/U$ sets a uniform distribution across the image (and so U is set to the size of the image) and w_b is set to the number of photons emitted by the background in image i (for our tests this was set to 5 photons per pixel).

Using this we can calculate the probability of observing a localization at point l in an image, by summing the false and true positive probabilities, with the result

$$P(l|i) = w_b\frac{1}{U} + \sum_{em\in E(i)} w_{em}\mathcal{N}(r|\mu_{em},\sigma_{em}). \tag{60}$$

Calculating $P(em|l,i)$ follows by the application of Bayes rule, giving the result

$$P(em|l,i) = \frac{P(em,l|i)}{P(l|i)} = \frac{w_{em}\mathcal{N}(l|\mu_{em},\sigma_{em})}{w_b\frac{1}{U} + \sum_{em\in E(i)} w_{em}\mathcal{N}(r|\mu_{em},\sigma_{em}}. \tag{61}$$

This is evaluated for all emitter/localization pairs, and hence used to calculate S.

Note that the background component b is not a member of $E(i)$. This is important when calculating

$$\max_{em\in E(i)} P(em|l,i) \tag{62}$$

as this ensures localizations which are most probably generated by background noise have a maximum value of $P(em|l,i)$ which is approximately 0, and so contribute little to S.

For the 3D localization case the classification was still performed in 2D rather than 3D. However the normal distribution $\mathcal{N}(l|\mu_{em}, \sigma_{em})$ was replaced with a non-isotropic 2D Gaussian $\mathcal{N}(l|\mu_{em}, \Sigma_{em})$, with a diagonal covariance matrix Σ_{em}. The diagonal elements of Σ_{em} were determined based on the emitter's axial depth in the Z direction, stretched according to the simulated astigmatism.

8 Supplementary Note - Estimating changes in activation density from photon count

When evaluating experimental data, it is difficult to evaluate the number of fluorophores emitting in each image unless the density is known to be one at which accurate single molecule fitting can be performed (for example, in our live cell experiments to evaluate imaging speed, we kept the density in a regime where we were confident that fitting would be accurate). However, if the activation density is so high that individual fluorophores are not accurately fitted, it becomes impossible to accurately estimate it. We have found a good proxy for the activation density to be the sum of the brightness, in photons, of the localisations, as estimated by ThunderSTORM.

If the activation density is low, then the vast majority of ThunderSTORM localisations will each correspond to a single fluorophore. In this case the total number of localisations returned will be approximately equal to the number of active fluorophores, while the sum of their estimated brightnesses will be the same number scaled by the average brightness of each emitter. However, if the activation density is too high, ThunderSTORM will begin to mistake groups of two or more fluorophores for a single one. In this case the total number of localisations returned will underestimate the number of active fluorophores, possibly by quite a significant factor. The sum of the brightnesses of the fluorophores, however, will not be so badly affected - the average estimated intensity of the returned localisations will rise by the very same factor that the number of localisations falls by, and so the sum cancels the two out.

Supplementary Fig. 7 demonstrates this relationship for the case of the 2D synthetic data, where an accurate estimate of the ground truth number of activations is available. ThunderSTORM was run on each dataset of 1000 images, returning a list of localisations each time. Without any further processing the average photon count per image was calculated by summing over the brightness of the returned localisations and dividing by 1000. The average number of localisations was calculated similarly. As we have access to the ground-truth activations from the simulations, this could be plotted against the average true number of fluorophore activations.

The relationship between the average number of photons and the average number of activations was approximately linear over a large range of activation densities, marking it as a good candidate proxy for the true activation density. However the total number of localisations, while initially linear, plateaued at around $4 - 5$ localisations per image, and thereafter began to significantly underestimate the true number of activations. Note that even when ThunderSTORM is grossly underestimating the number of active fluorophores, the sum of their brightnesses still varies

linearly. As such, where access to ground truth activations is not available, we use this to estimate the relative number of activations instead.

9 Supplementary Note - Emitter and camera simulations

When creating the synthetic image sequences we attempted to ensure they were as faithful as possible to the sort of images observed in practice, so as to be certain that any observed phenomena were not artefacts. The simulation process can be broken into three steps.

In the first step, emitters were uniformly randomly distributed throughout a pre-defined sample shape, with an additional constraint that no emitters can attach within 2 nm of one another.

In the second step, photon emissions were simulated from each emitter. This was modelled by allowing each emitter to take one of three states - on, off, and bleached. The probabilities of these are given in Supplementary Table 1. States were updated in time steps of size 0.5 ms. At each time step, an emitter in the on state emitted a random number of photons. This random number was drawn from a Poisson distribution, with a typical mean of 25. As the camera was simulated running at 100 Hz with a 1 ms dead period, a typical emitter which was active for an entire image acquisition period will generate approximately 500 photons, of which 450 will be captured by the simulated camera.

Emitters stochastically change between the on and off states. The probability of transition between the states was varied between simulations, so as to alter the probability of each emitter being active in a given image. Additionally, emitters in the on state had a probability of moving into the bleached state. Once in the bleached state they remain there permanently. All emitters were initialised in the on state. This resulted in extremely dense activations in the early portion of the image sequence which caused QuickPALM to perform spurious fits, so the first 200 frames were discarded. Only the subsequent 1000 images were collected to form the sequence that was later analyzed. The output of this stage was a list of which emitter was active at each time step, and how many photons it emits.

In the third and final stage, the known time and location of photon emissions were used to generate an image. The camera was simulated running at 100 Hz, with a 1 ms dead period at the beginning of each exposure to simulate the write time. This was used, in conjunction with the time of emission of each photon, to assign photons to exposures. A Bessel function shaped point spread function was used to randomly perturb the position of each photon, where the width of the Bessel function was set assuming a wavelength of 488 nm, and a NA of 1.4. During 3D analysis any stretching of the point spread function along one axis or another due to astigmatism was simulated at this stage, by linearly stretching the position of the emitter photons relative to the emitter position. This allowed calculation of which camera pixel each photon was incident to (a pixel size of 110 nm was assumed). Lastly, errors introduced by the simulated EMCCD camera

were based on the model discussed in [27] . Errors at the detector stage were modelled by replacing the photon count of each pixel with a sample drawn from a Poisson distribution, with a mean equal to the number of incident photons multiplied by the quantum efficiency (set at 0.7) plus a contribution from the dark current (simulated as a Poisson distributed variable with a mean of 5 photons, per pixel). Errors in the register stage were modelled using a cascade of 536 Poisson processes (an approximation first suggested by [28]) with $\lambda = 0.01$.

10 Supplementary Note - Change in exclusion region when using a multi emitter fitting algorithm

Multi emitter fitting algorithms, in which a region of the image which has been identified as containing active fluorophores may have multiple point spread functions fitted to it rather than one, allow the analysis of regions of a sample at higher activation densities than can be achieved using single emitter models. This in turn allows more fluorophores to be imaged at once, and so seems likely to reduce the average acquisition time necessary to achieve a particular quality of reconstruction.

This fits naturally into the framework we have already provided. The acquisition time is determined by the activation density a, and the size of the exclusion region $|\mathcal{S}_\epsilon(x)|$. If the size of the exclusion region can be shrunk then the maximum usable activation density grows correspondingly. However, the exclusion region around x is defined explicitly as the size of the tagged portion of the sample which, when a fluorophore activates at x, cannot contain any more active emitters if that fluorophore is to be accurately localised. By allowing multiple emitters to be fitted to a single region with overlapping point spread functions, multi emitter fitters reduce the size of the exclusion region, and hence increase the maximum usable activation density.

An example for a similar dataset to that used in Supplementary Method 11 is shown in Supplementary Fig. 8, comparing Thunderstorm when run in both single and multi emitter mode on a simulated dataset of two emitters whose separation is linearly varied. As can be seen, the single emitter begins to introduce localisation errors at a separation of ≈ 600nm, and fails completely by 400nm. The multi emitter, however, continues to identify two distinct emitters down to a separation of ≈ 200nm.

11 Supplementary Note - Estimating exclusion region with a higher NA objective

Given the size of the exclusion region, and hence imaging speed, is in part determined by the apparent size of the point spread function, the question can be asked as to whether an improvement in imaging speed can be achieved simply by using a higher numerical aperture objective. This will reduce the size of the point spread function, increasing the density of localisations which can be successfully resolved.

All our simulations and experiments use a 1.4Na objective. Increasing the numerical aperture beyond this is not impossible, but is difficult and expensive. Hence, we have instead opted to simulate the effect of using a 1.7 NA objective, and in particular study whether there is a significant change in the size of exclusion region. This was performed in exactly the same way as Supplementary Method 1, with the ThunderSTORM and QuickPALM results shown in Supplementary Fig 9 and 10 respectively.

While there is a visible different in the performance of both algorithms using the higher numerical aperture objective, the separation at which the algorithms fail, and their failure mode, is virtually unchanged in both cases. As such there does not seem to be much benefit to further increasing the NA of the objective, especially given the associated cost.

However, the same is not true of changing the camera pixel size, as shown Supplementary Fig. 11. Here, ThunderSTORM is used with two cameras, one with pixels of width 110nm and one with 35nm. Here the two separate emitters can be isolated down to a separation of ≈ 200nm, potentially allowing much more rapid acquisition. The price that is paid, though, is an increase in the standard deviation of the localisations, due to the higher relative contribution of the background level.

1 Supplementary References

References

1. Betzig, E. *et al.* Imaging intracellular fluorescent proteins at nanometer resolution. *Science* **313**, 1642–1645 (2006).

2. Hess, S. T., Girirajan, T. P. & Mason, M. D. Ultra-high resolution imaging by fluorescence photoactivation localization microscopy. *Biophys J.* **91**, 4258–4272 (2006).

3. Rust, M. J., Bates, M. & Zhuang, X. Sub-diffraction-limit imaging by stochastic optical reconstruction microscopy (STORM). *Nat. Methods* **3**, 793–796 (2006).

4. Wolter, S., Endesfelder, U., Van de Linde, S., Heilemann, M. & Sauer, M. Measuring localization performance of super-resolution algorithms on very active samples. *Opt Express* **19**, 7020–7033 (2011).

5. Mukamel, E. A. & Schnitzer, M. J. Unified resolution bounds for conventional and stochastic localization fluorescence microscopy. *Phys. Rev. Lett.* **109**, 168102 (2012).

6. Rees, E. J., Erdelyi, M., Schierle, G. S. K., Knight, A. & Kaminski, C. F. Elements of image processing in localization microscopy. *J Opt* **15**, 094012 (2013). URL `http://stacks.iop.org/2040-8986/15/i=9/a=094012`.

7. Nieuwenhuizen, R. P. J. *et al.* Measuring image resolution in optical nanoscopy. *Nat. Methods* **10**, 557 – 562 (2013).

8. Ober, R. J., Ram, S. & Ward, E. S. Localization accuracy in single-molecule microscopy. *Biophys J* **86**, 1185–1200 (2004).

9. Small, A. R. Theoretical limits on errors and acquisition rates in localizing switchable fluorophores. *Biophys J* **96**, L16–L18 (2009).

10. Lin, Y. *et al.* Quantifying and optimizing single-molecule switching nanoscopy at high speeds. *PLoS ONE* **10**, e0128135 (2015).

11. Dempsey, G. T., Vaughan, J. C., Chen, K. H., Bates, M. & Zhuang, X. Evaluation of fluorophores for optimal performance in localization-based super-resolution imaging. *Nat. Methods* **8**, 1027–1036 (2011).

12. Van de Linde, S., Wolter, S., Heilemann, M. & Sauer, M. The effect of photoswitching kinetics and labeling densities on super-resolution fluorescence imaging. *J.of Biotechnol* **149**, 260 – 266 (2010).

13. Holden, S. J., Uphoff, S. & Kapanidis, A. N. DAOSTORM: an algorithm for high-density super-resolution microscopy. *Nat. Methods* **8**, 279 (2011).

14. Dertinger, T., Colyer, R., Iyer, G., Weiss, S. & Enderlein, J. Fast, background-free, 3D super-resolution optical fluctuation imaging (SOFI). *Proc. Natl. Acad. Sci. U.S.A.* **106**, 22287–22292 (2009).

15. Huang, F., Schwartz, S. L., Byars, J. M. & Lidke, K. A. Simultaneous multiple-emitter fitting for single molecule super-resolution imaging. *Biomed. Opt. Express* **2**, 1377–1393 (2011).

16. Cox, S. *et al.* Bayesian localization microscopy reveals nanoscale podosome dynamics. *Nat. Methods* **9**, 195–200 (2012).

17. Zhu, L., Zhang, W., Elnatan, D. & Huang, B. Faster storm using compressed sensing. *Nat. Methods* **9**, 721–723 (2012).

18. Sinkó, J. *et al.* Teststorm: Simulator for optimizing sample labeling and image acquisition in localization based super-resolution microscopy. *Biomed. Opt. Express* **5**, 778–787 (2014). URL `http://www.osapublishing.org/boe/abstract.cfm?URI=boe-5-3-778`.

19. Henriques, R. *et al.* Quickpalm: 3d real-time photoactivation nanoscopy image processing in imagej. *Nat. Methods* **7**, 339 – 340 (2010).

20. Ovesnỳ, M., Křížek, P., Borkovec, J., Švindrych, Z. & Hagen, G. M. Thunderstorm: a comprehensive imagej plug-in for palm and storm data analysis and super-resolution imaging. *Bioinformatics* **30**, 2389–2390 (2014).

21. Heilemann, M. *et al.* Subdiffraction-resolution fluorescence imaging with conventional fluorescent probes. *Angew. Chem. Int. Ed. Engl.* **47**, 6172–6176 (2008).

22. Breiman, L. Random forests. *Machine Learning* **45**, 5–32 (2001).

23. Shtengel, G. *et al.* Interferometric fluorescent super-resolution microscopy resolves 3d cellular ultrastructure. *Proc. Natl. Acad. Sci. U.S.A.* **106**, 3125–3130 (2009).

24. Goffin, J. M. *et al.* Focal adhesion size controls tension-dependent recruitment of α-smooth muscle actin to stress fibers. *The Journal of Cell Biology* **172**, 259–268 (2006).

25. Kanchanawong, P. *et al.* Nanoscale architecture of integrin-based cell adhesions. *Nature* **468**, 580 – 584 (2010).

26. Case, L. B. *et al.* Molecular mechanism of vinculin activation and nanoscale spatial organization in focal adhesions. *Nat. Cell Biol* **Advance online publication** (2015). Http://dx.doi.org/10.1038/ncb3180.

27. Hirsch, M., Wareham, R. J., Martin-Fernandez, M. L., Hobson, M. P. & Rolfe, D. J. A stochastic model for electron multiplication charge-coupled devices–from theory to practice. *PloS One* **8**, e53671 (2013).

28. Tubbs, R. N. Lucky exposures: Diffraction limited astronomical imaging through the atmosphere. *Observatory* **124**, 159–160 (2004). `astro-ph/0311481`.

29. Mortensen, K. I., Churchman, L. S., Spudich, J. A. & Flyvbjerg, H. Optimized localization analysis for single-molecule tracking and super-resolution microscopy. *Nat. Methods* **7**, 377–381 (2010).

30. Holtzer, L., Meckel, T. & Schmidt, T. Nanometric three-dimensional tracking of individual quantum dots in cells. *Applied Physics Letters* **90**, 053902 (2007).

31. Huang, B., Wang, W., Bates, M. & Zhuang, X. Three-dimensional super-resolution imaging by stochastic optical reconstruction microscopy. *Science* **319**, 810–813 (2008).

32. Pavani, S. R. P. *et al.* Three-dimensional, single-molecule fluorescence imaging beyond the diffraction limit by using a double-helix point spread function. *Proc. Natl. Acad. Sci. U.S.A.* **106**, 2995–2999 (2009).

33. Juette, M. F. *et al.* Three-dimensional sub-100 nm resolution fluorescence microscopy of thick samples. *Nat. Methods* **5**, 527–529 (2008).
