# Supplementary material for: Local dimensionality determines imaging speed in localization microscopy
Source: Nat Commun. 2017 Jan 12;8:13558. doi: 10.1038/ncomms13558 (PMC5241698; doi:10.1038/ncomms13558)
Supplement: Supplementary Software — Random Forest post processing method for localisation microscopy data. [file ncomms13558-s2.zip › NCOMMS13558 SI software/User_Guide.pdf]

User Guide for reclassifying localisation microscopy data

This is the user guide for the Random Forest post processing method for localisation microscopy data. We're going to show all our demonstrations using Matlab R2014a, installed on a Linux Ubuntu 14.04 operating system. Some of the menus may look a bit different if your operating system or version of Matlab is different, but the functionality is the same.

Things you need to do/know first:

- Perform your PALM/STORM experiment.
- **Save the image sequence as a series of individual images** in their own folder somewhere. Note that this technique cannot currently examine .tiff image stacks or .nd2 files – you need to separate them out into individual images. If your microscope software will not do this for you, you can use ImageJ or Fiji to export your data (you may need a package such as Bioformats Importer to import your files).
- **Analyse the results** using a localisation algorithm, and save the fluorophore positions in a **.csv file**. This .csv file **must** contain an X and Y co-ordinate for each localisation, as well as the **number of the frame in the time sequence** where the localisation took place. Almost all localisation algorithms supply this information. (Example data and an example .csv file are provided in the directory which contains your software, in the subdirectory example_images).
- You need a working copy of **Matlab** with the **Statistics and Machine Learning toolbox** installed.
- You'll need some time to sit and label a number of localisations for the Random Forest classifer to be trained with.

To start with, you need to open Matlab.

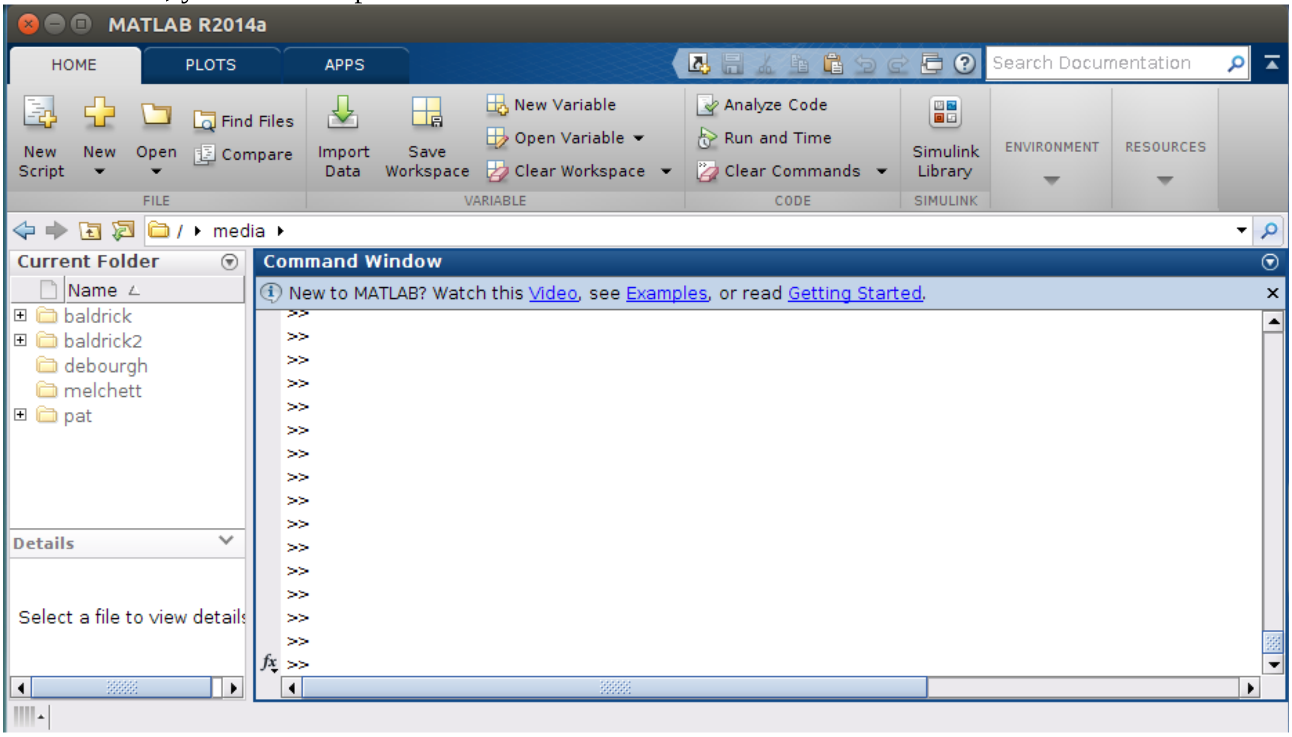

Once you've opened Matlab, you need to change directory into the folder in which the supplied .m files have been stored. This can be done using the cd (change directory) command. In the example shown the directory with the software is pat/DATAPART2/sampling_paper/software/, but yours will be different.

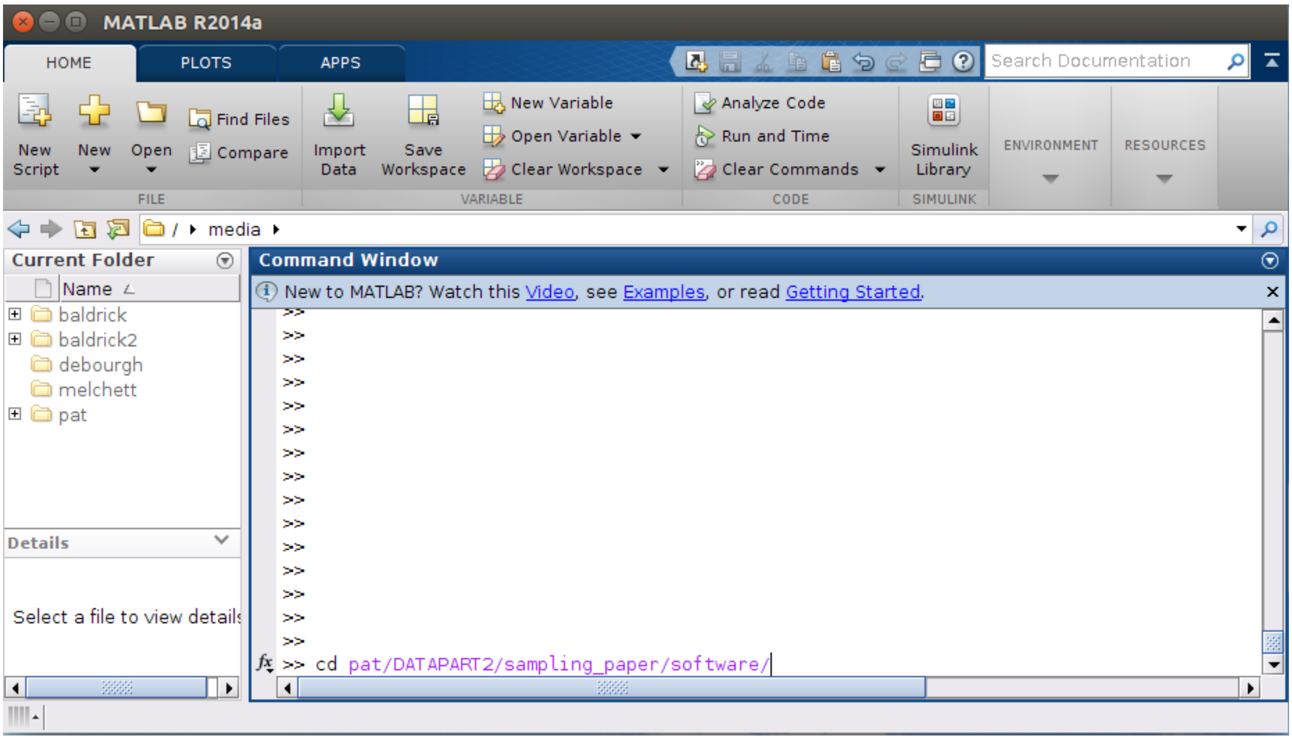

There should be 7 files in there:

- START.m
- label_samples.m
- make_features_from_scratch.m
- run_training.m
- stratisample.m
- train_tree.m
- display_results.m
- DISPLAY.m

There will also be a directory called example_images. This contains 26000 images (analysed data presented in the paper). The name of the first image is img00000.tiff and the rest are numbered sequentially. There is also a file of localised molecule positions, thunderstorm.csv.

To start, you just need to run START().

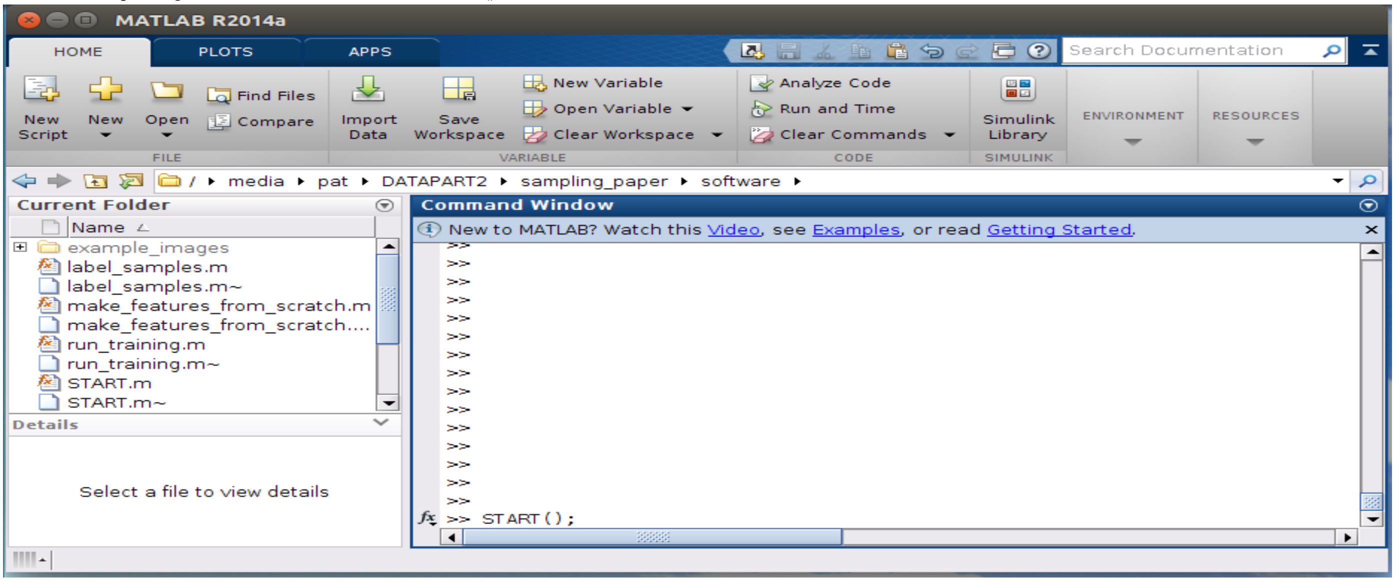

The first thing which will happen is a window will pop up, like this:

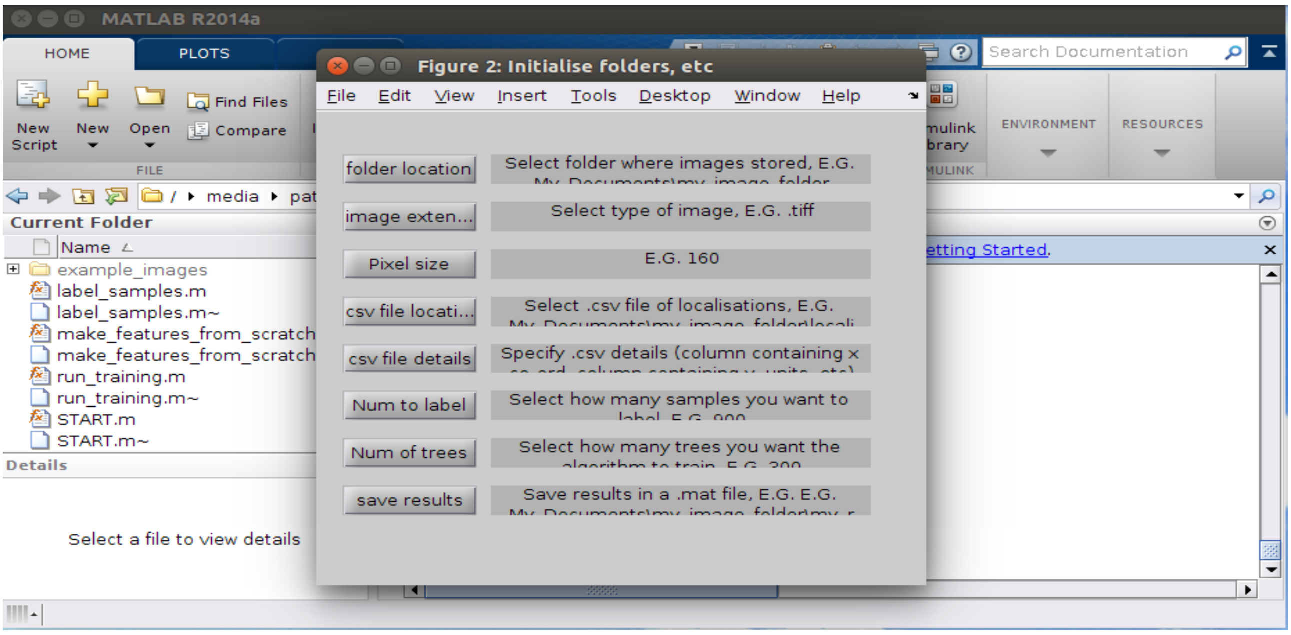

It's probably not going to be the right size to read clearly, so you can change that by dragging the corners like normal:

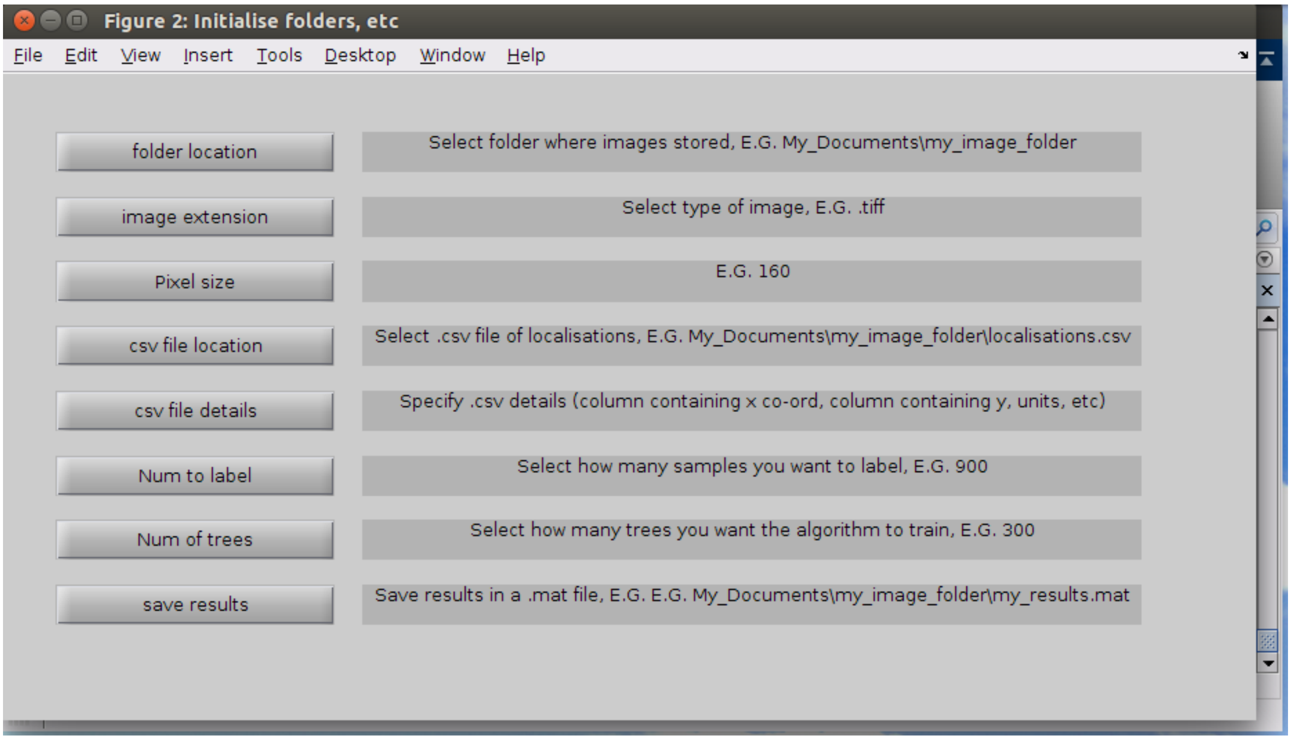

Each of the buttons in this window corresponds to some piece of information the algorithm needs. Let's start at the top and click 'folder location'. That should open a popup window like this:

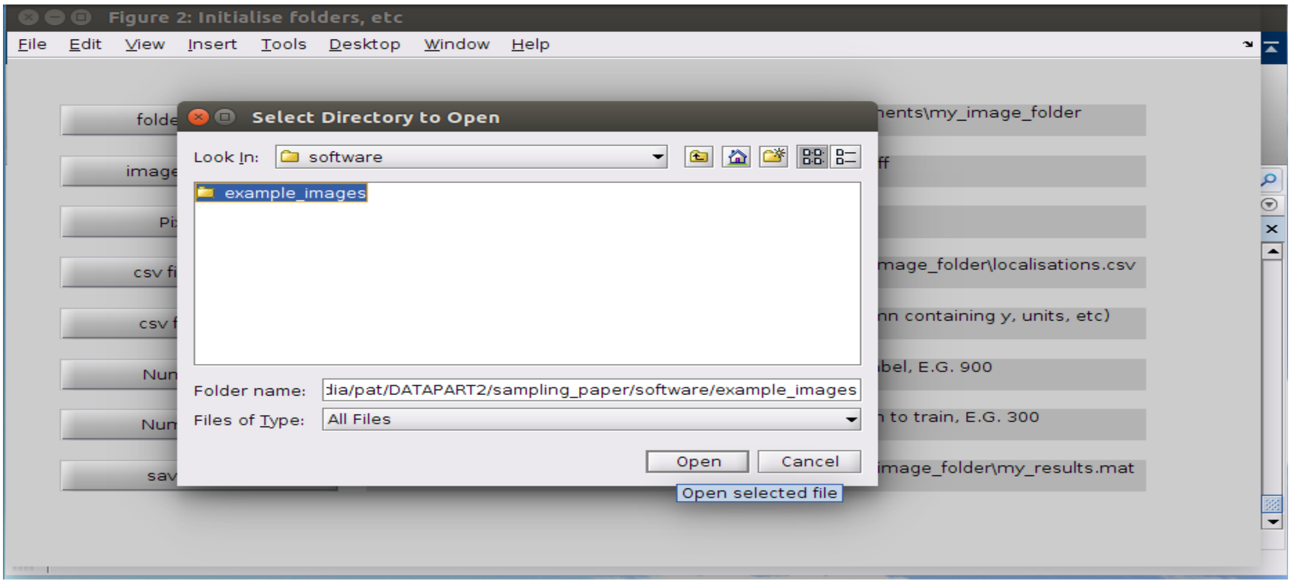

This is asking for the *folder in which the images you analysed using super resolution are stored*.

As a slight digression, let's take a quick look at what I've got stored in that folder:

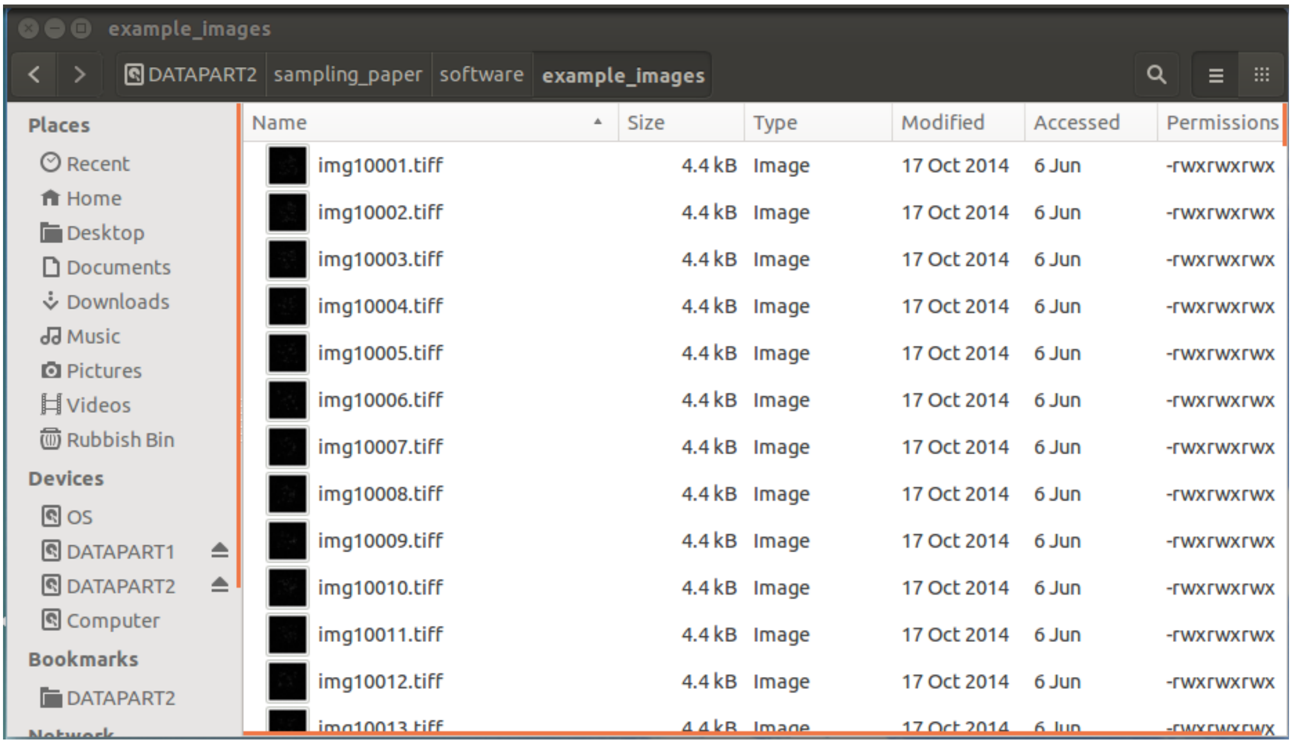

This folder is full of the images which were analysed by the localisation algorithm. This is because the random forest classifier has to go back to the raw data to get enough information to perform its classification – it can't just work on the localisations alone. Note also that each image is saved as a separate file, there must be no other images in that folder with the same file extension, and the images must be in order (so the first image in the folder is the first image which your localisation algorithm analysed).

We'll go back to Matlab now:

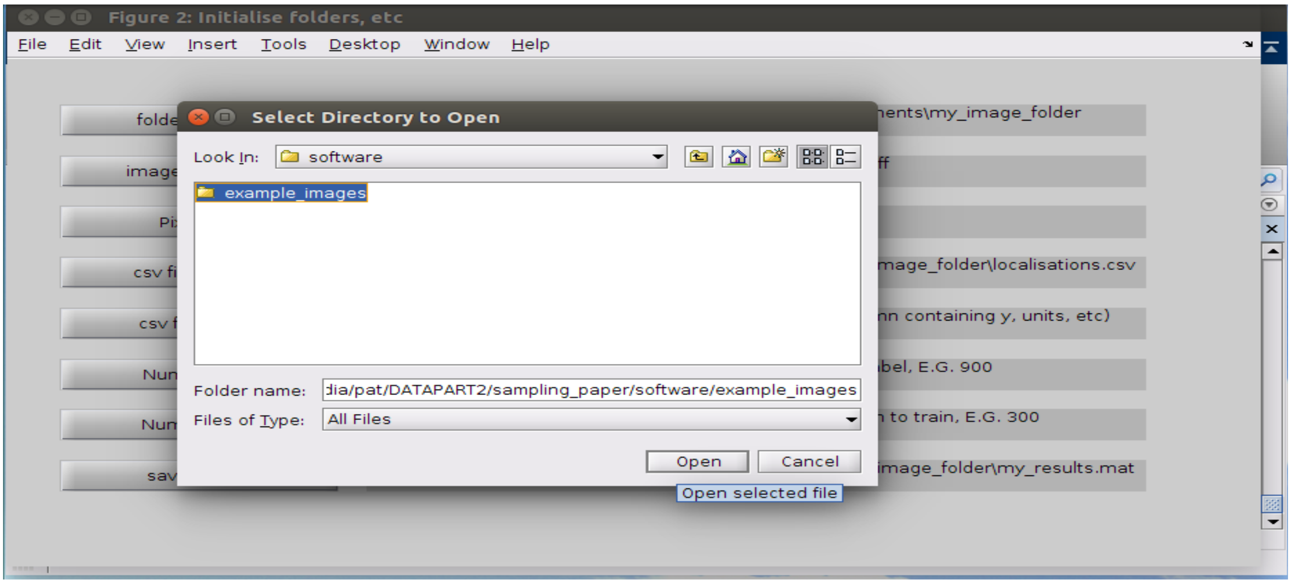

When you've found the correct folder, highlight it and click 'Open'. I'm going to select example_images. The popup window will disappear, and the window with lots of buttons will look something like this:

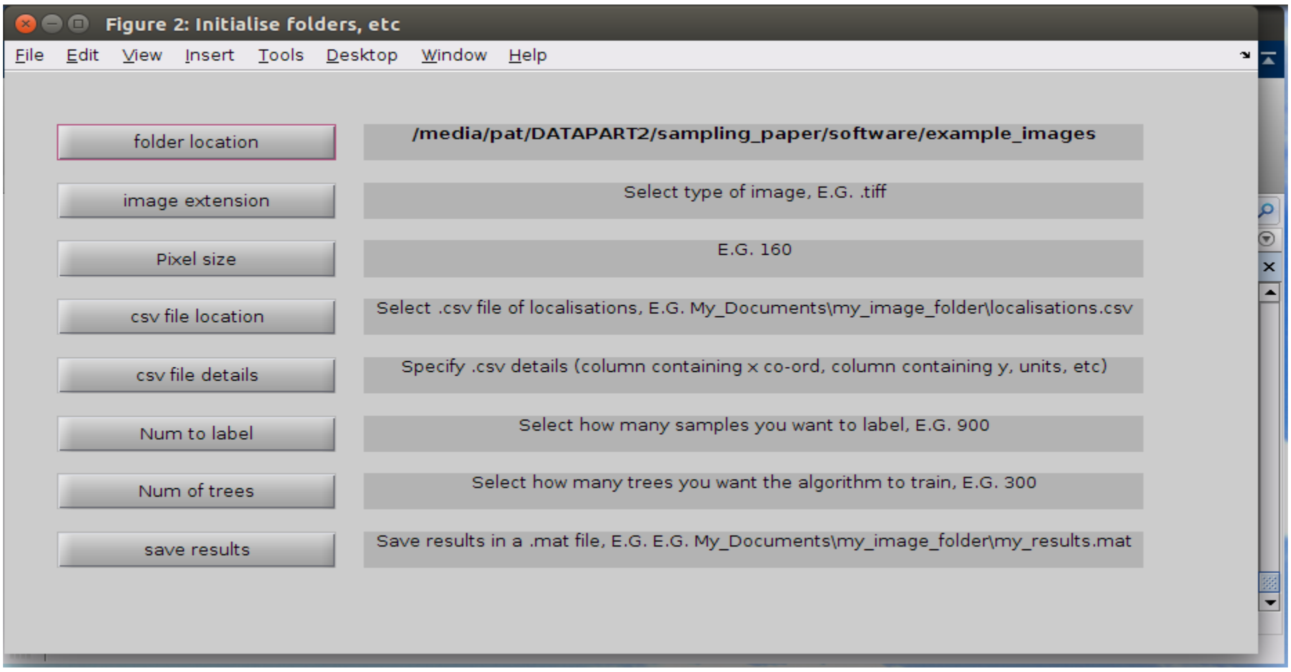

As you've selected a folder, the text next to that button has gone bold, and tells you which folder you've selected (in case you want to check you've got the right one).

The next button down sets the image extension. Click it and this should happen:

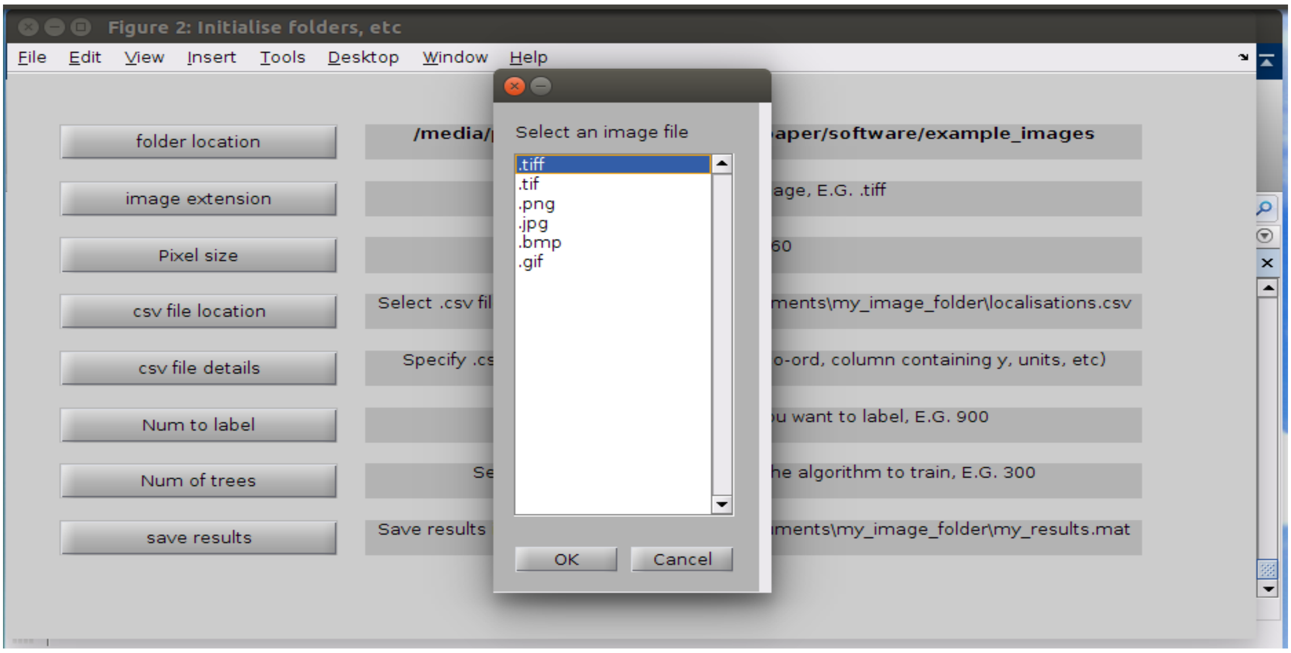

A new popup window asks what kind of images are stored in that folder. My images are all saved as .tiff, so I'll leave it on the default and click 'OK'. Note that when you do this, Matlab will check the folder it's expecting to find the images in and make sure it can find at least one image with your chosen file extension. If not, you get an error box like this:

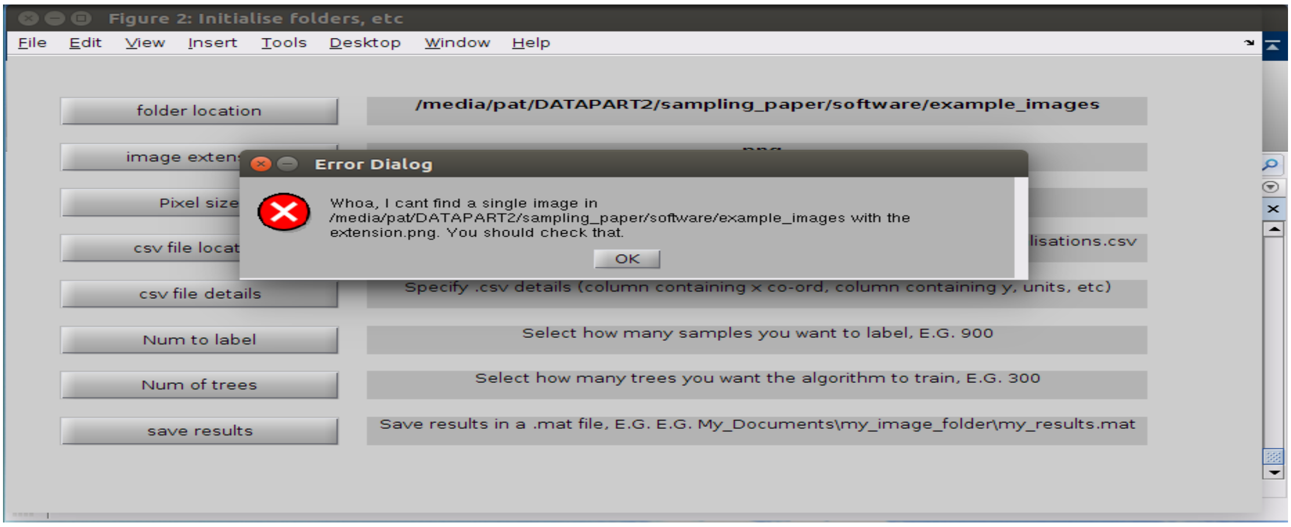

If you see this, check you've selected the folder with your images in, and used the right file extension (remember that Matlab will treat '.tif' and '.tiff' as being different).

The next button let's us set the pixel size. Click it and this happens:

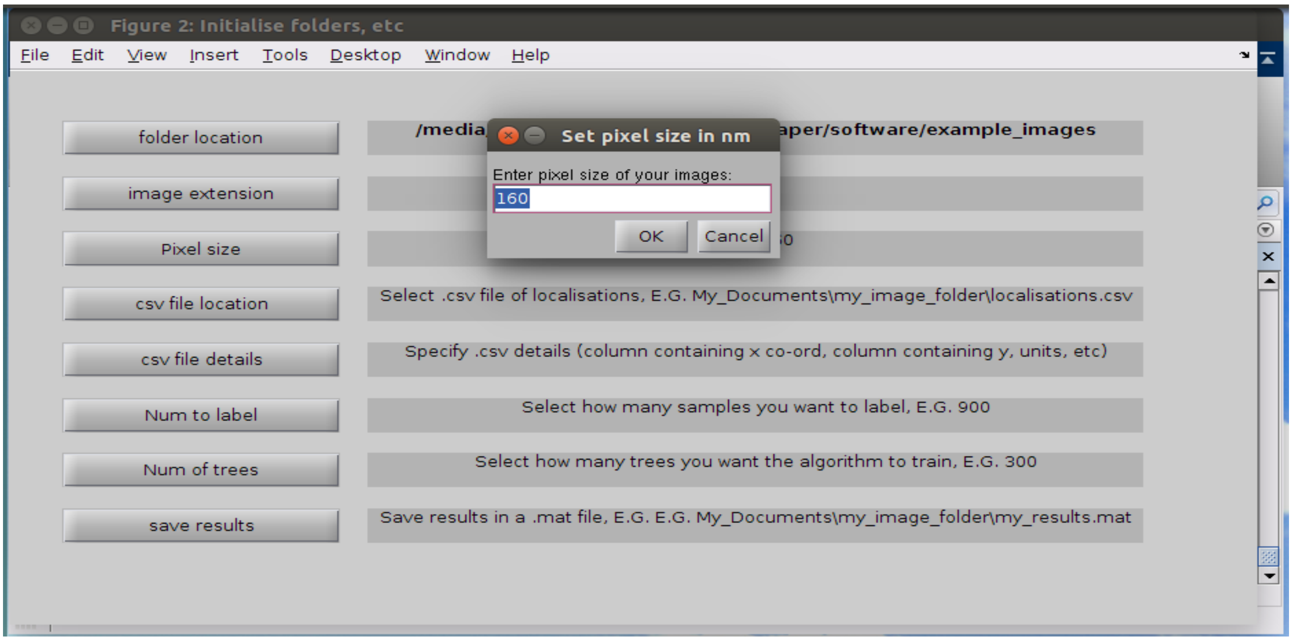

This is asking for the size of the pixels in nm. We need this information as some localisation algorithms return estimated fluorophore positions in nm, so to correlate this with the image information, we need to be able to rescale appropriately.

The next button asks us to select the .csv file in which the localisations are stored. Another popup window will appear, asking you to select a file.

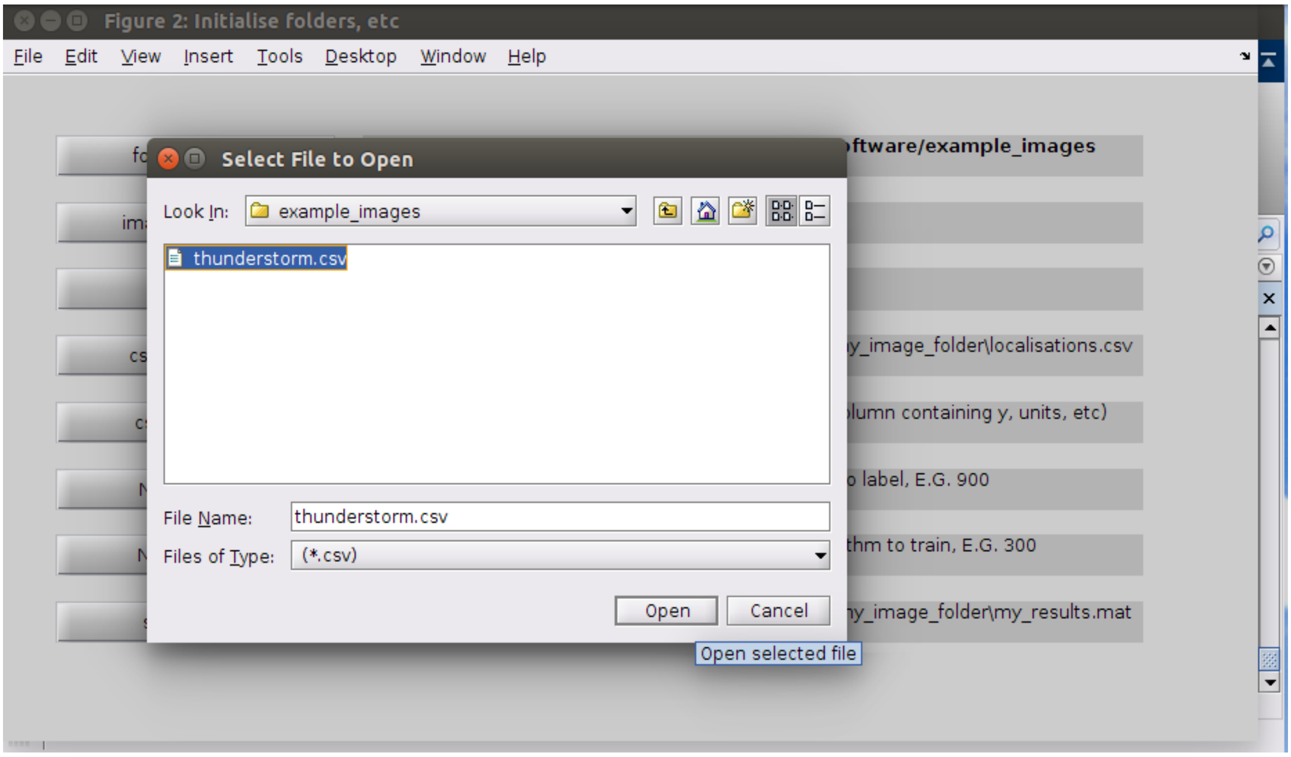

Most localisation algorithms I use give the option to save the results in a .csv file. If yours doesn't, then so long as you can read them into Matlab somehow you can use the csvwrite() function to create your own .csv file.

I've used ThunderSTORM as my localisation algorithm in this case, and saved the results in thunderstorm.csv in the same folder my raw images are stored in. So I'm going to select that and

click 'Open'.

The next button is going to let us set some details about how the data is stored in the .csv file. Click it and you get something like this:

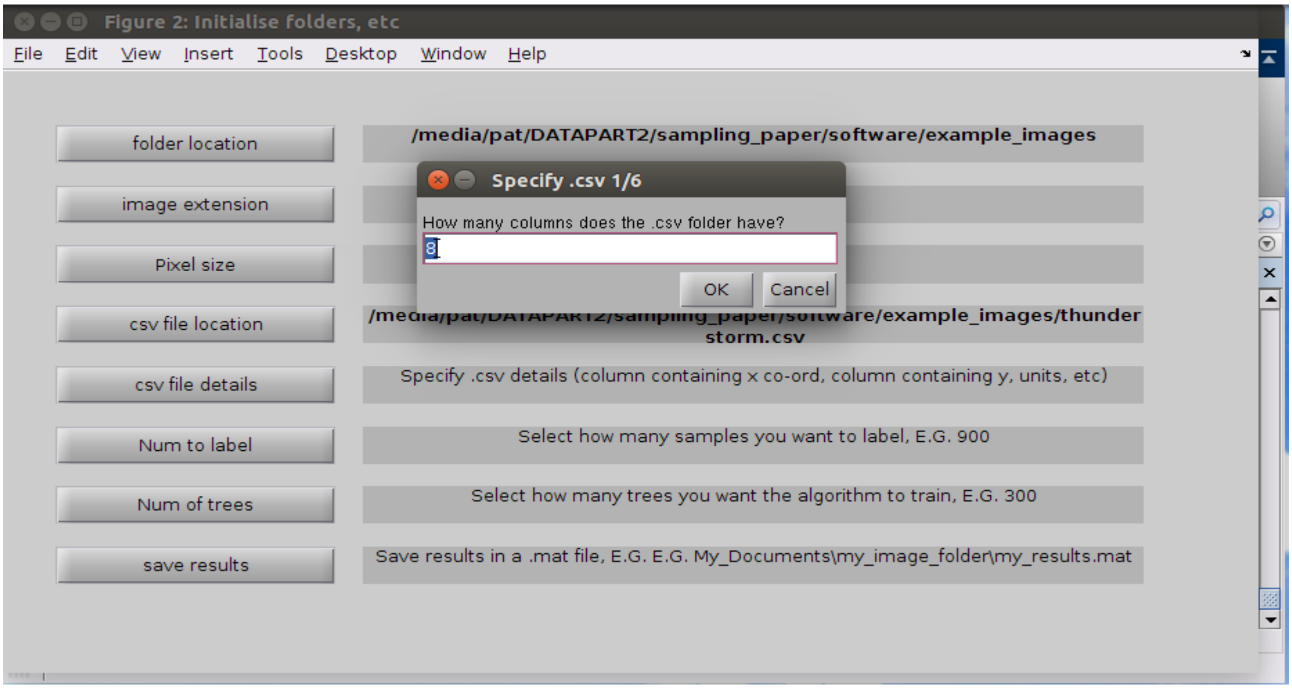

This is where you tell the classifier which columns in the .csv file store the information it needs. If you've created your own .csv file, or use this particular algorithm a lot, then you probably know this. If not, then no problem – let's just open the .csv file and take a look. I'll navigate to the folder my .csv file is in and find it:

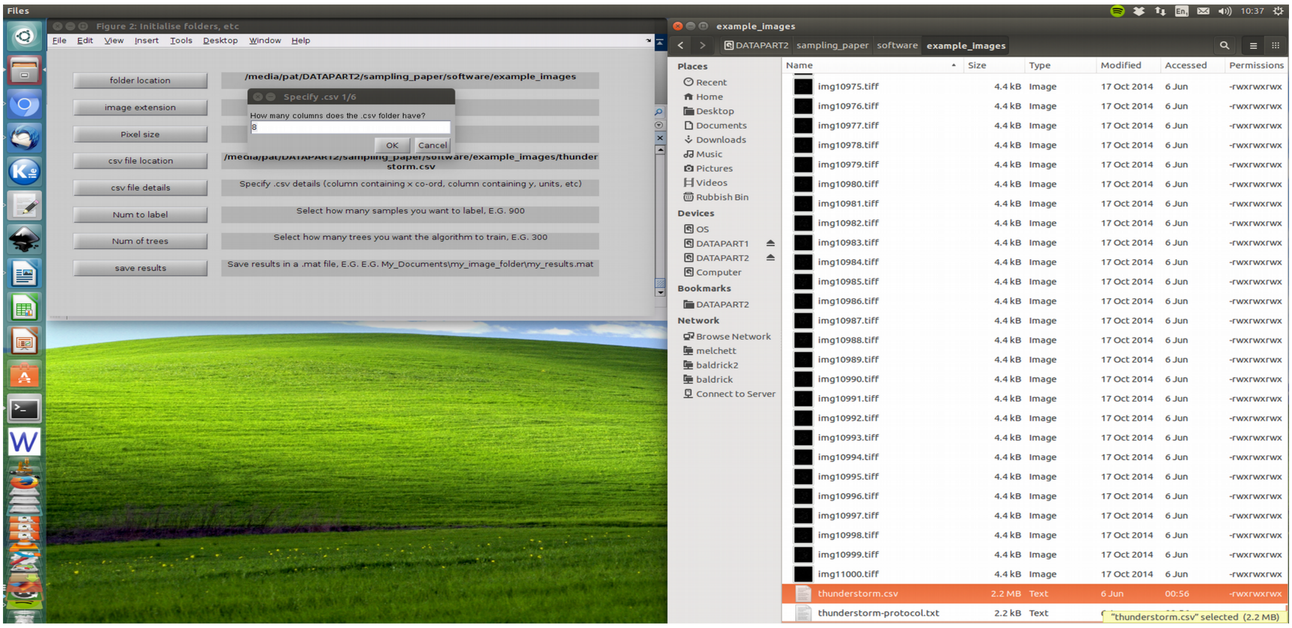

The file I want is thunderstorm.csv. Double click this and it should open. Excel, Libre Office, gedit, notepad, etc can all open .csv files. My computer will open it using Libre Office:

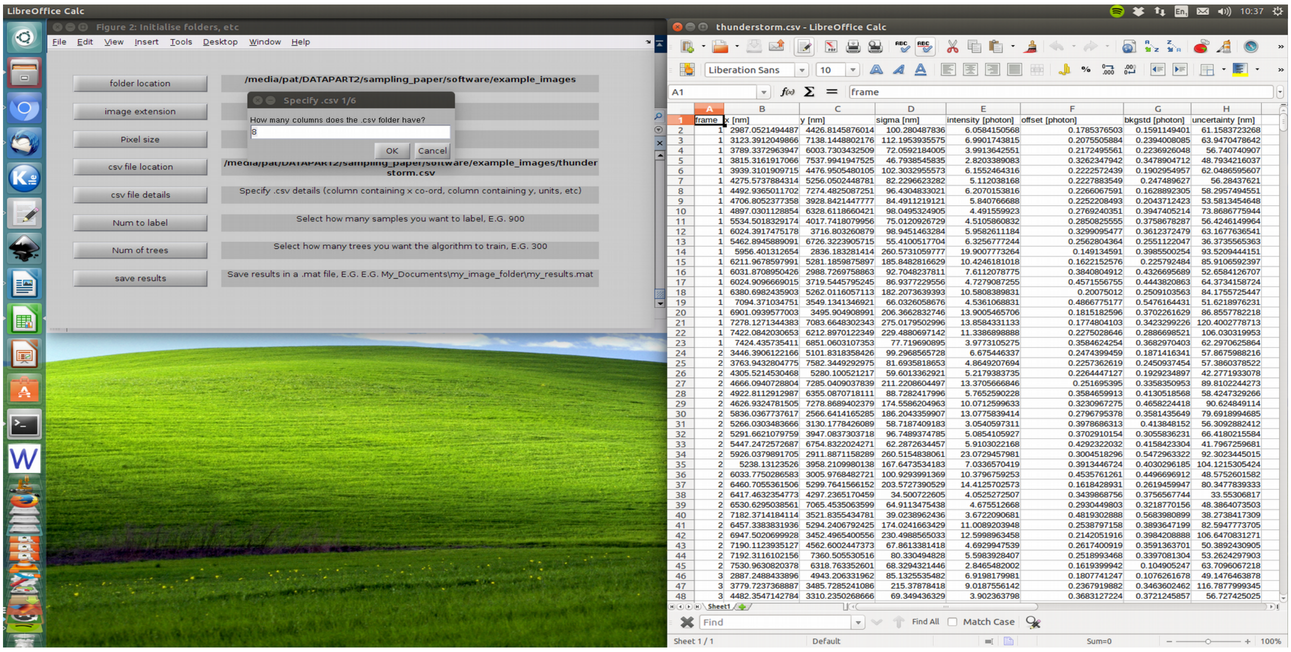

And it looks like that. Let's just zoom in on the top right hand corner:

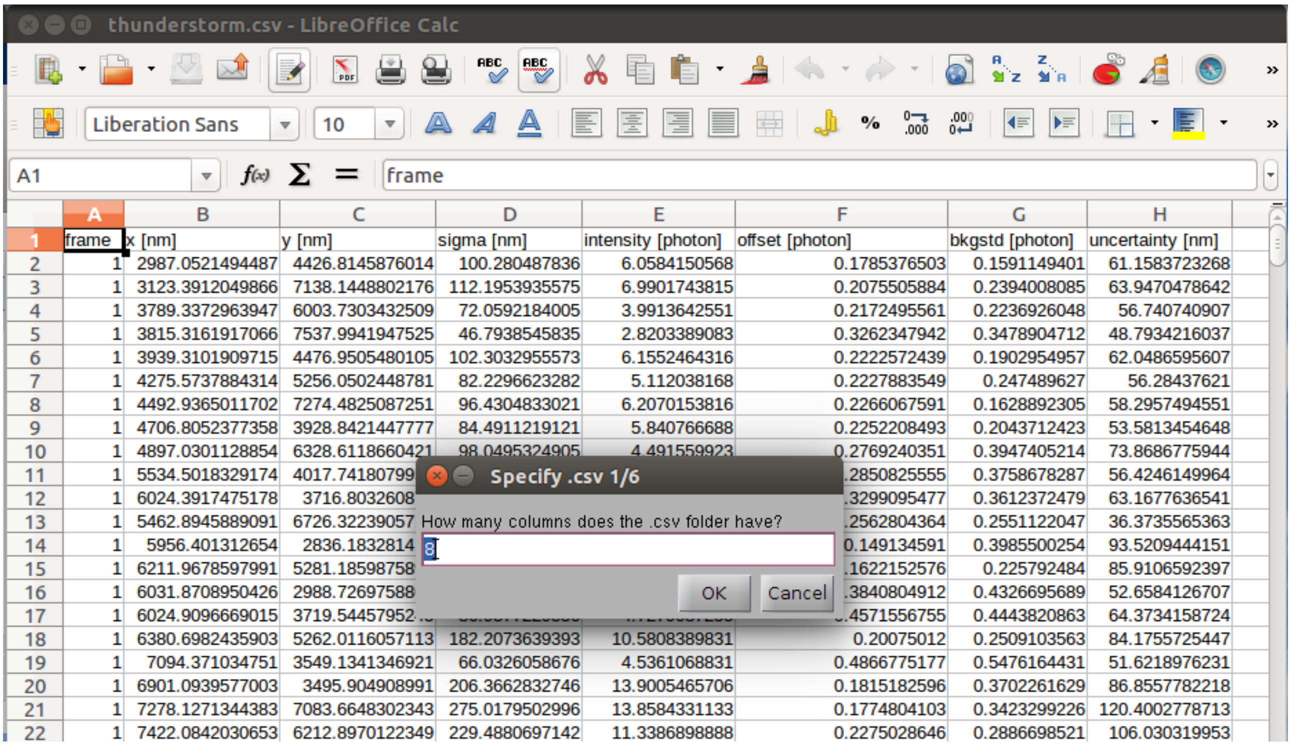

The first piece of information we need is how many columns are in the file. This is to help Matlab parse it later. We can see there are 8 columns, so enter that and hit 'OK':

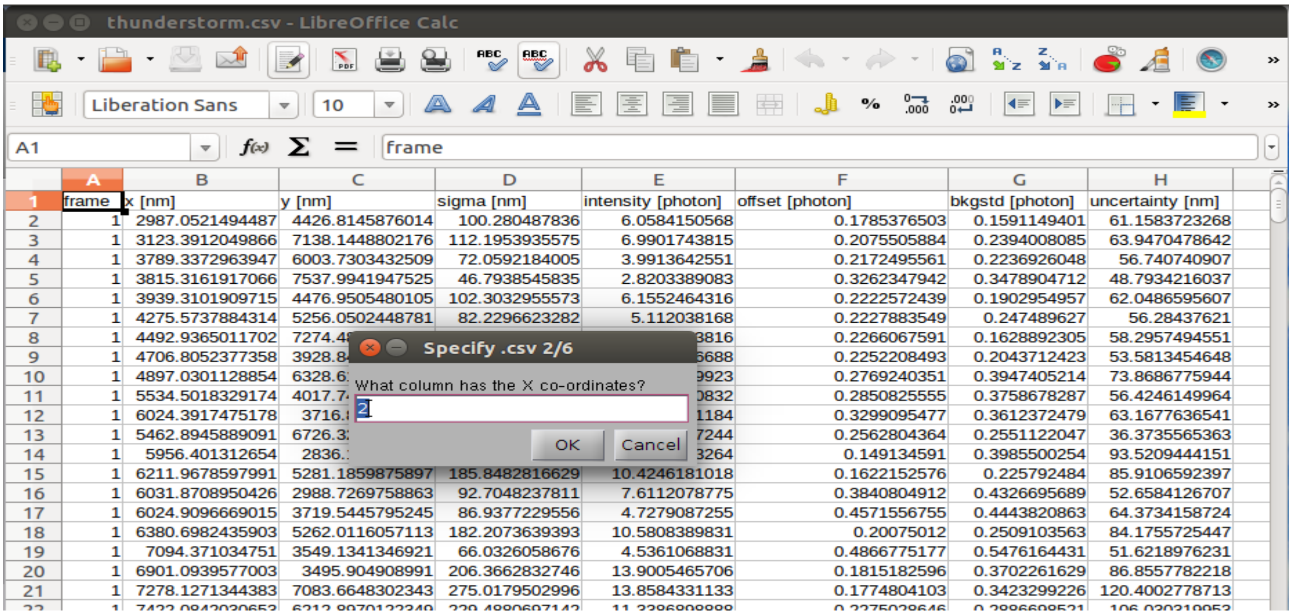

Next,we need to know which column has the X co-ordinate of the localisations. In this file, that's the second column, to set it to 2 and hit 'OK'.

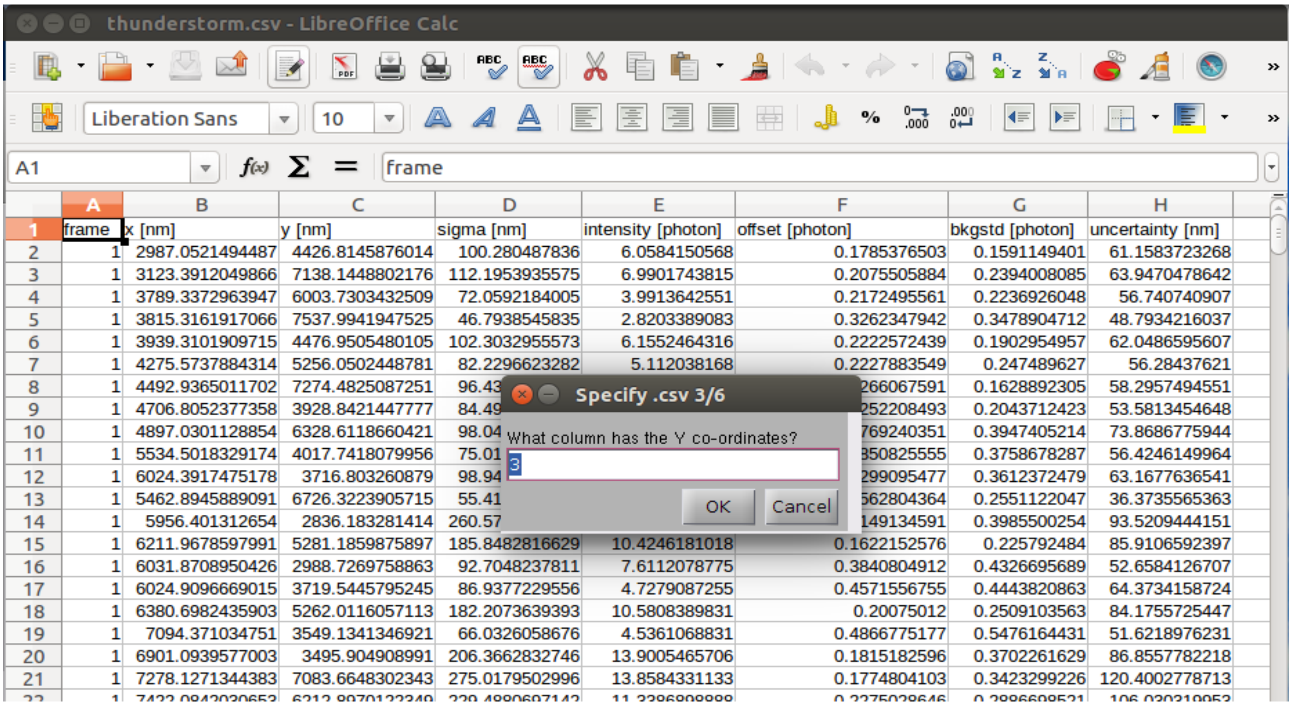

And now we need the Y column. That's just column 3. Enter and hit 'OK'.

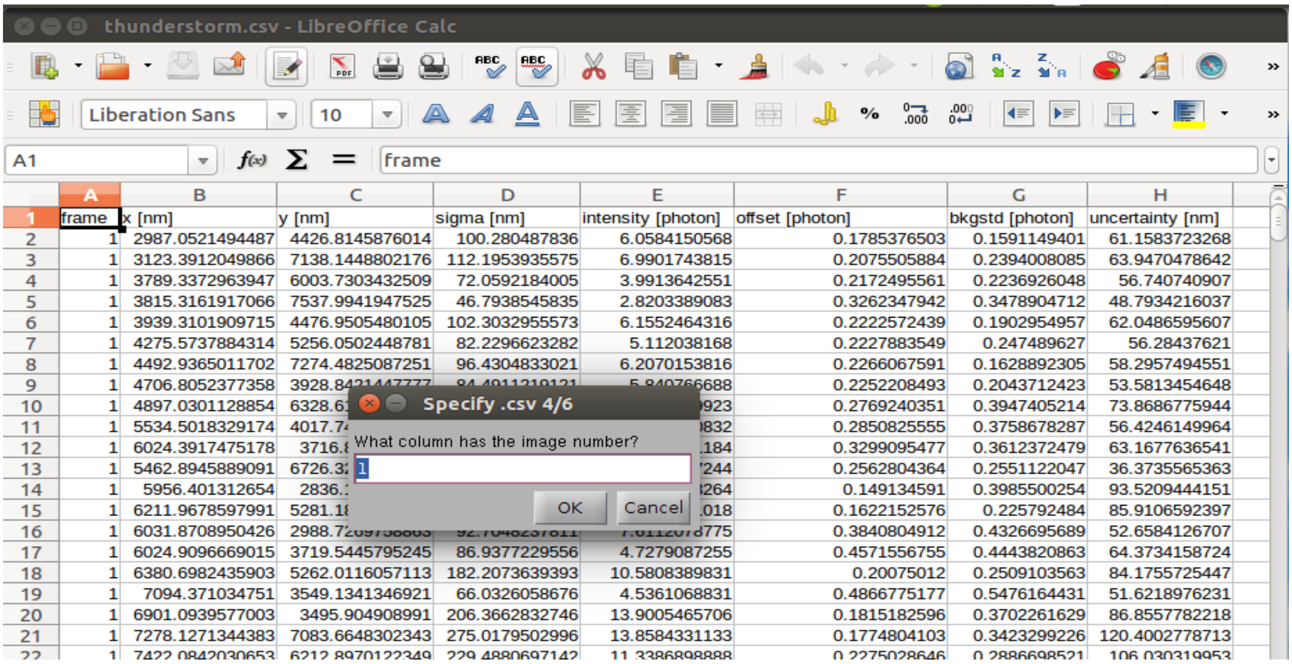

The next box asks for the 'image number'. Each localisation your algorithm returns ought to tell you what image it corresponds to – so if it found a molecule in the first image in the sequence, it ought to store a 1 in the corresponding row of the .csv file. In ThunderSTORM, this is the first column, so I'll set that to 1 and hit OK.

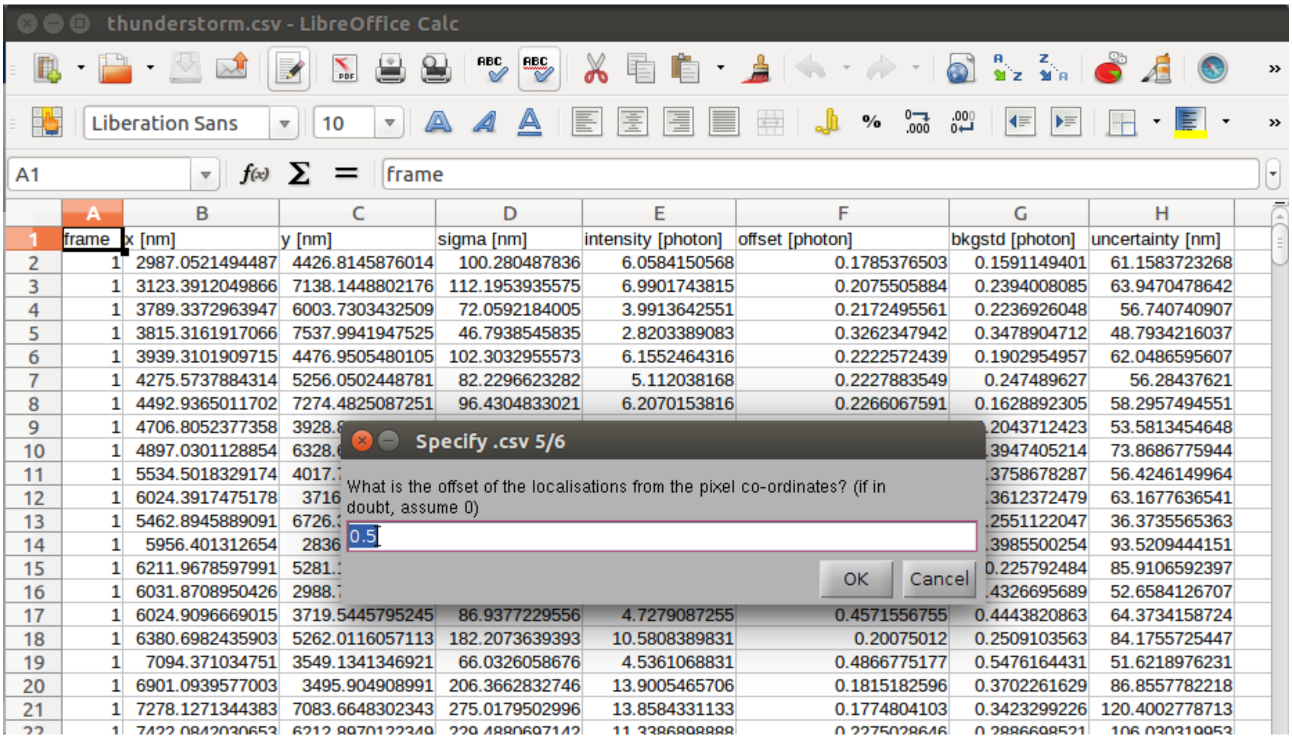

The next window asks what the *offset* of the localisations are, in pixel co-ordinates. This depends on the co-ordinate system your localisation algorithm uses – basically, if we were to plot the point (0,0) on the image, would that correspond to the *middle* of a pixel, or the *top left corner* of a pixel?

If you're not sure then the best way to check is to create an image with a single simulated

fluorophore in it, at a position you know, then run your localisation algorithm on the result and find out what co-ordinates it gives.

If you don't know this value then it's not a disaster. The classifier will not behave quite as well as this will introduce a degree of rotation dependency, but with enough training data it should be able to overcome this.

For ThunderSTORM, I know that the offset is 0.5 pixels, so I'll enter that and hit 'OK'

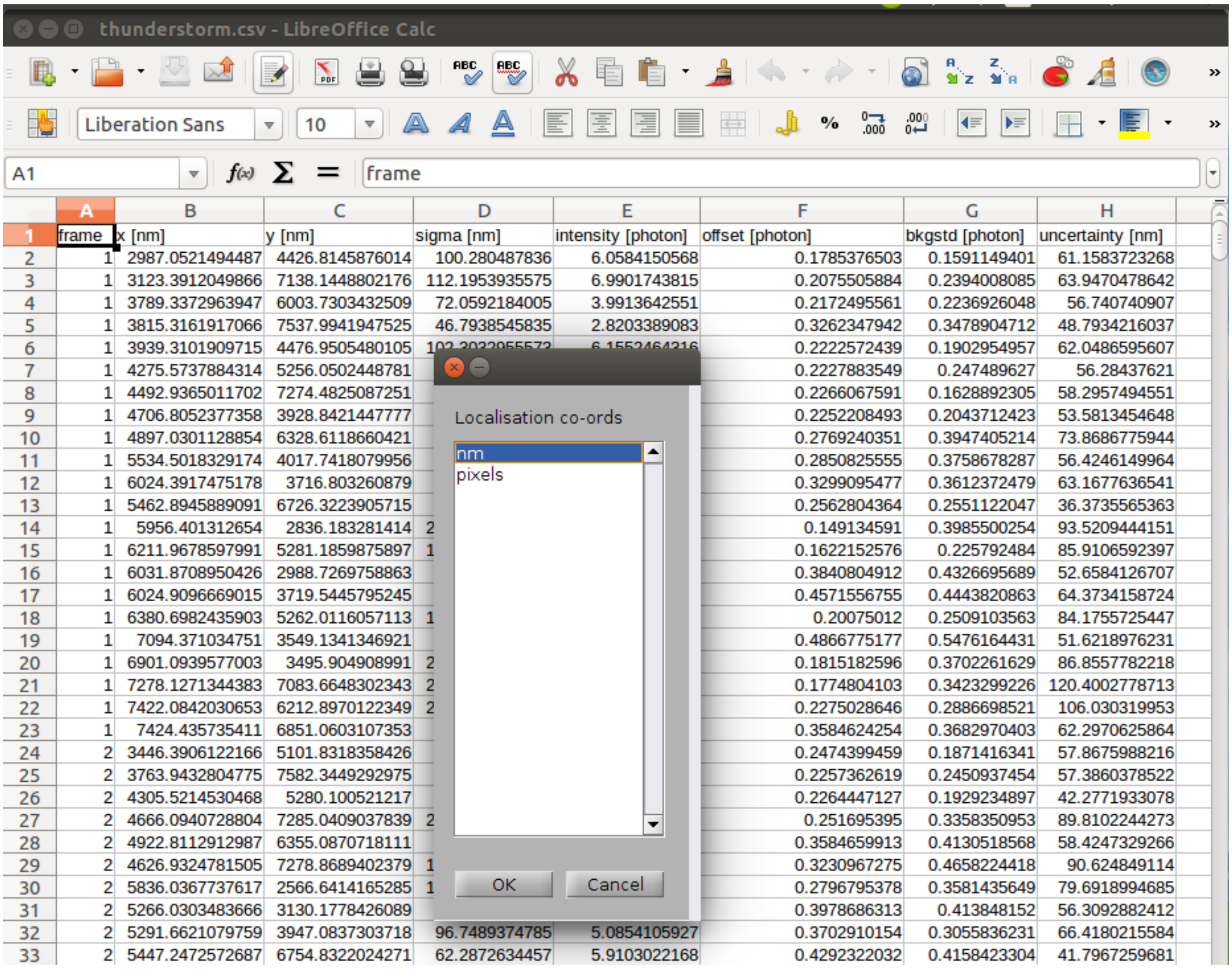

The co-ordinates of the localisations is the last part. This is asking whether the columns with the X and Y co-ordinates in are saved in terms of pixels or nm. ThunderSTORM saves in nm, so I'll select that and press OK.

That's all the information we need about the .csv file. If we go back to the window with all the buttons, it now is like this:

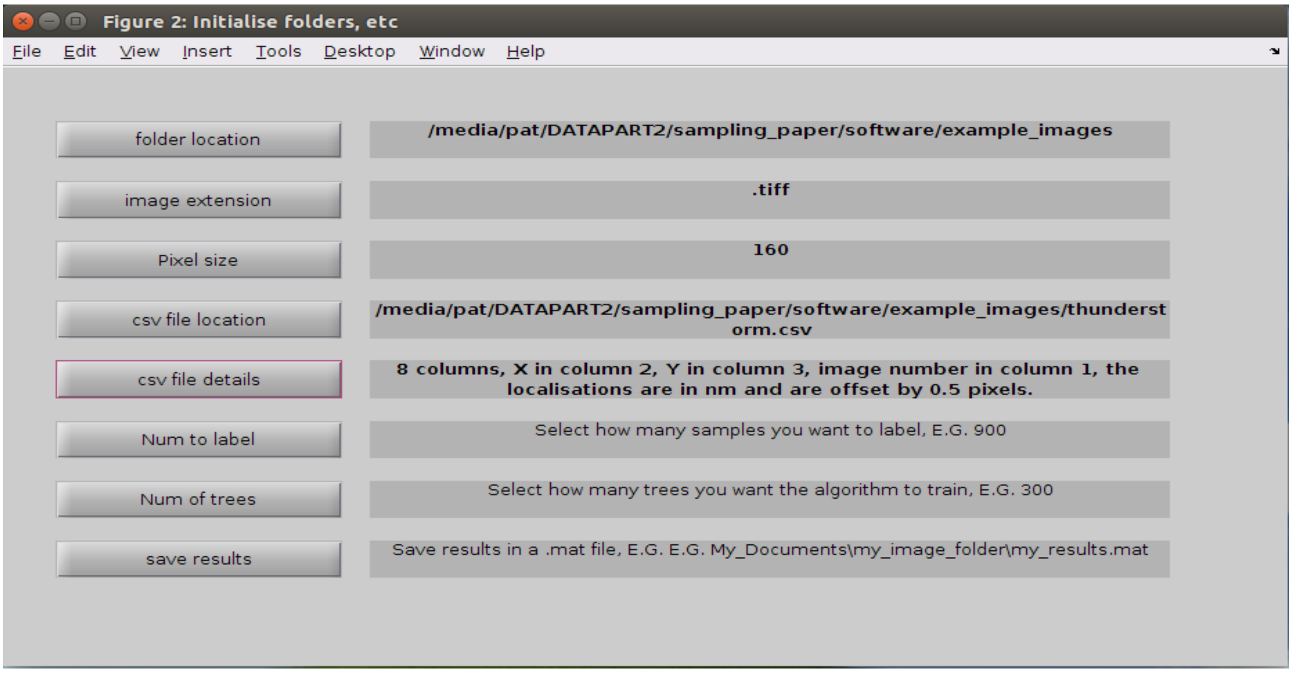

The details of the .csv file are shown in bold, so you can check them.

The next button sets how many localisations you're going to label:

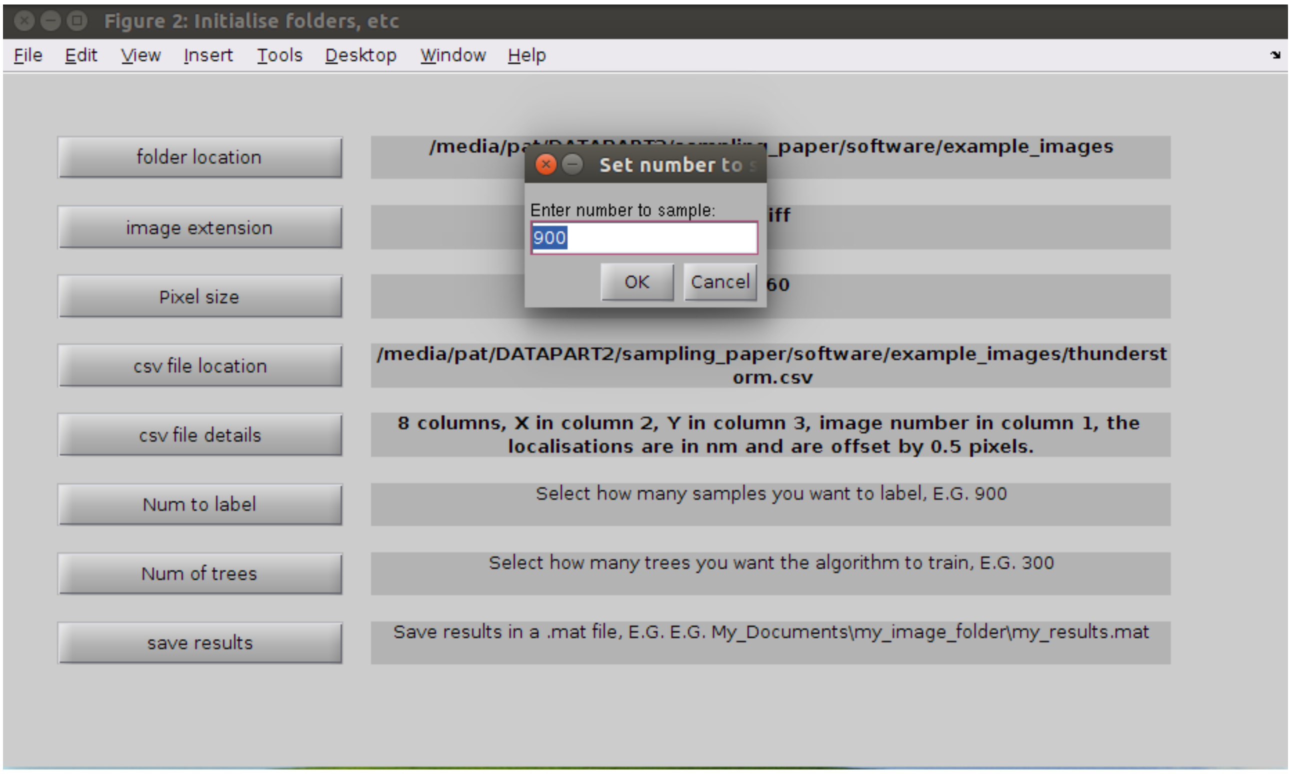

Labelling localisations is done in a separate popup window, which we'll explain in a moment. For each one, you'll be asked whether you judge the localisation to be accurate or inaccurate. The more of these you're willing to do, the better the classifier will be. I recommend 900, which I can label in about an hour, but we've built decent classifiers using just 300 in the past. It depends how much the appearance of your fluorophores varies during the image sequence, how much the illumination changes, etc, etc.

The next window asks you to set the number of trees in the random forest:

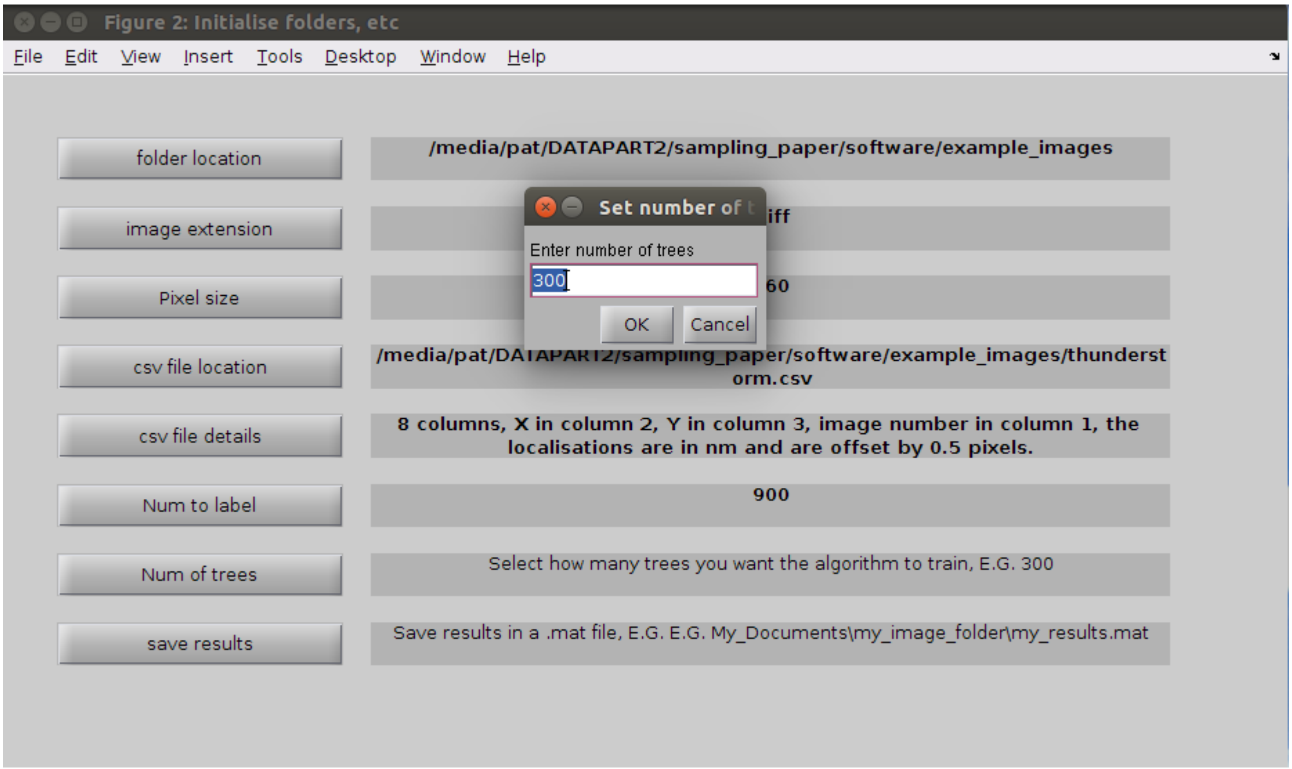

Training trees for the forest is something the algorithm will do at the very end. A single decision tree is an unbiased, high variance classifier. Random Forests train very many decision trees and average their result – this remains unbiased, but lowers the variance. There's really no downside to setting this number to be very high. Each tree will slightly increase the training time, and also the run time, but they're very quick compared to a lot of algorithms so this shouldn't be an issue. And as they make use of randomisation of the training data as part of their input, there's no real danger of them overfitting either.

The last window asks you to set the location to save the results:

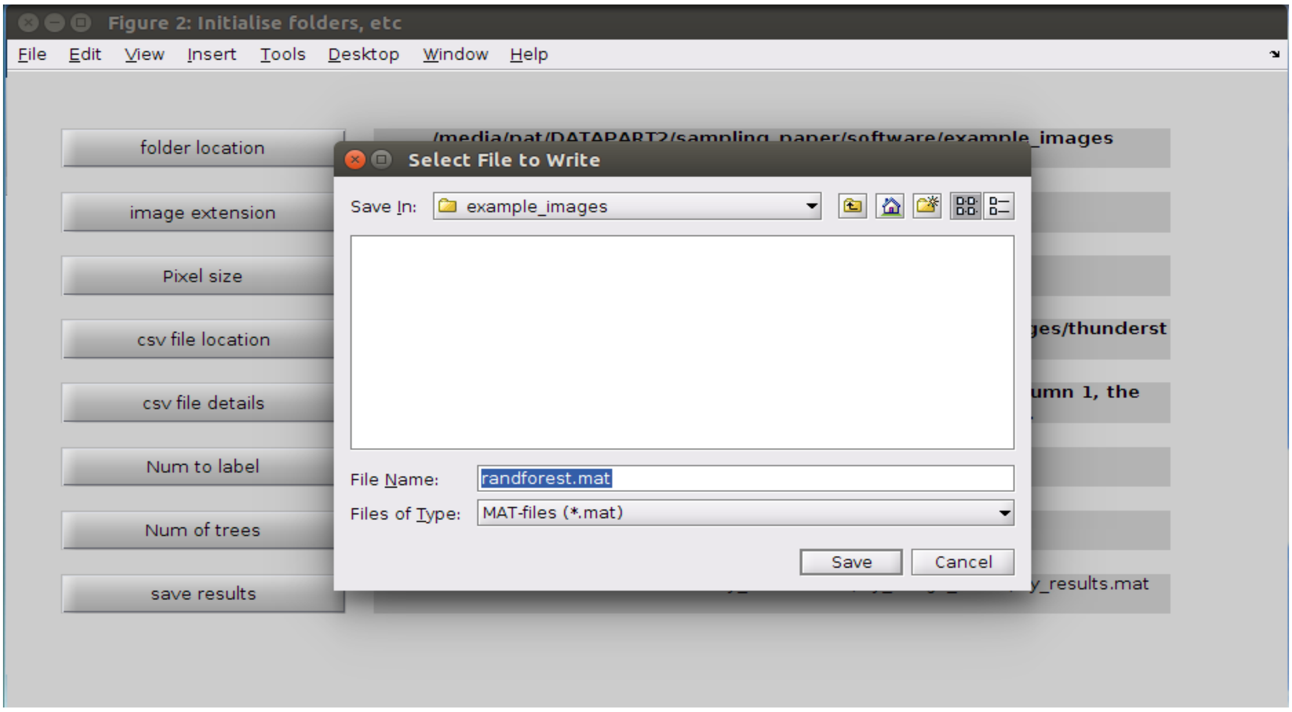

The results are saved as a .mat file, which you can read using Matlab later. The default location is the same folder your images are kept in, and the default filename is 'randforest.mat', but you can change these to anything you like. If you keep the name ‘randforest.mat’ and there is already a file of that name, you will be asked if you want to overwrite the file.

The window with the buttons in should look like this now:

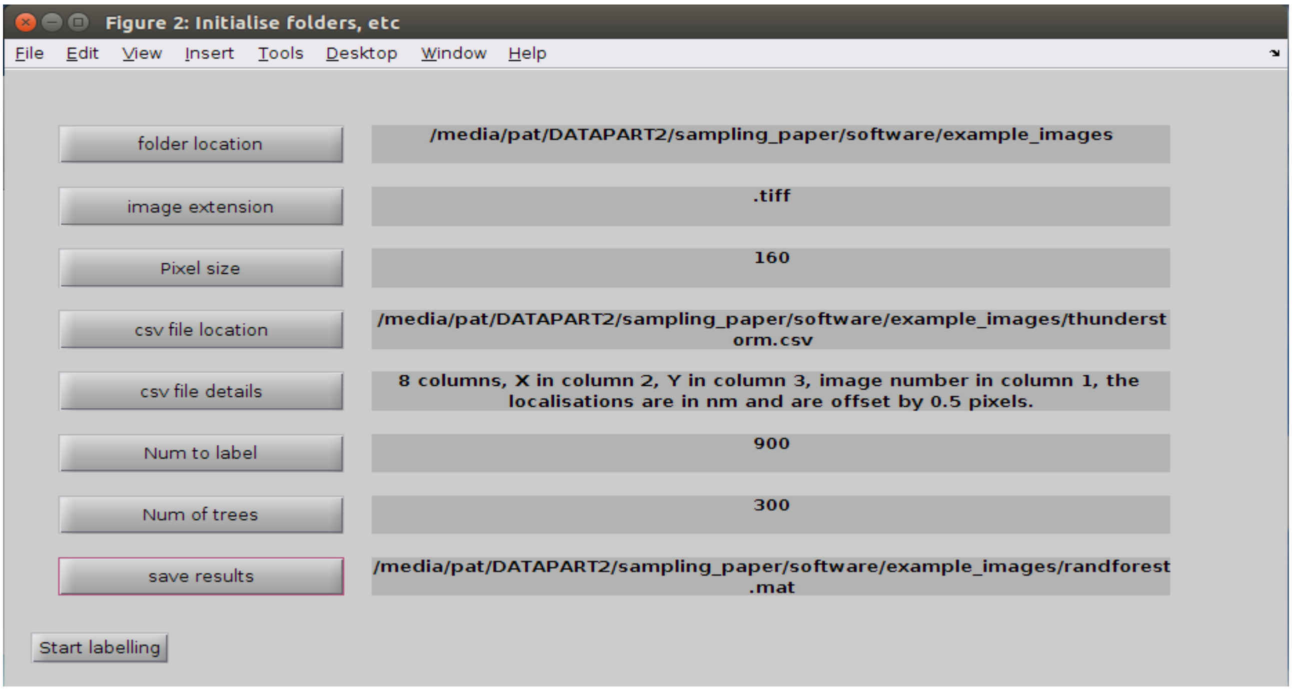

Now that we've set all the details, a new button has appeared, asking you to start the labelling process. When you're ready, click it.

The first thing you'll see is something like this:

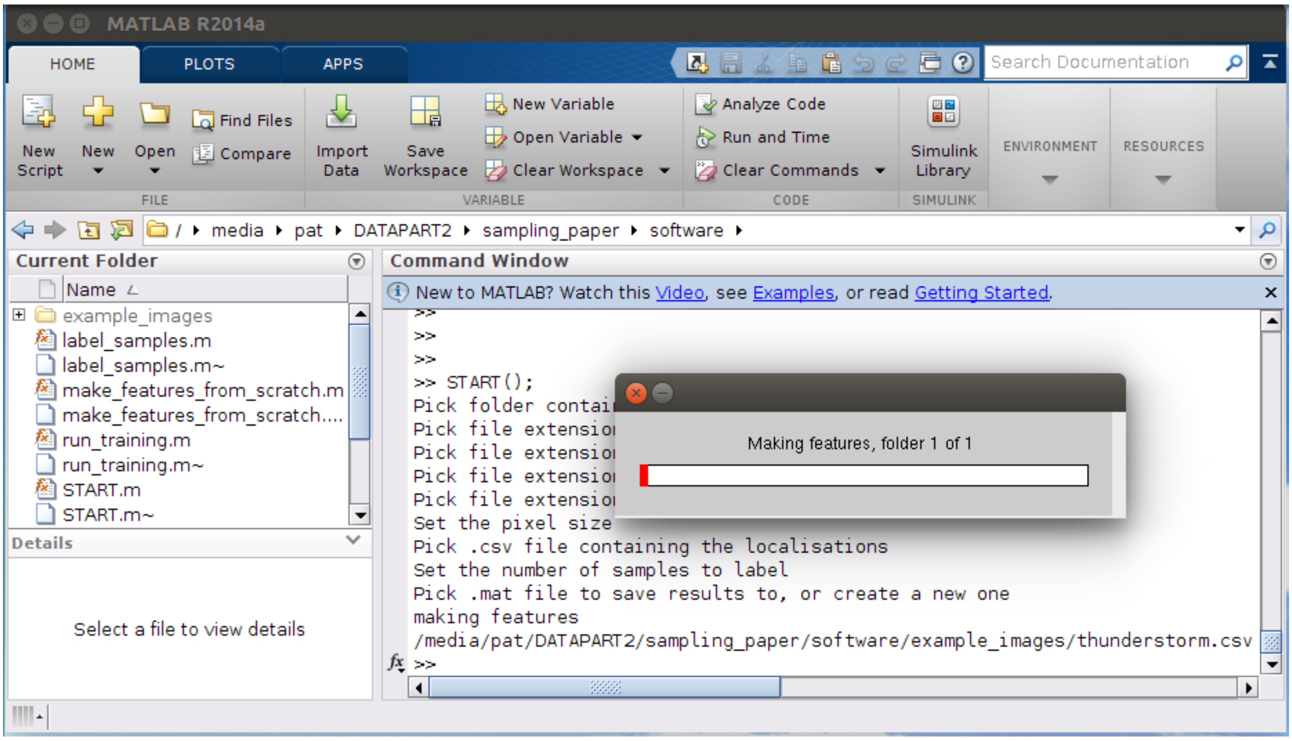

Random forests require two parts to their training data – a feature vector, and a label. The feature vector is the input to the classifier, and the label is the thing it tries to predict. In our case the label for each localisation is going to be whether or not it's accurate. The feature vector draws information from the following sources:

- The pixel values of the local area around the localisation (±10 pixels)
- The pixel values of the same region in the image which came immediately *before*
- The pixel values of the same region in the image which came immediately *afterwards*
- The numeric values saved in each column of the .csv files *other than* the x co-ordinate, y co-ordinate and image number.

These are transformed to the of of their absolute values, and Principal Component analysis applied to select the 20 components which capture most of the resulting variance. This creates a 20 dimensional feature for each localisation. Note that as the features rely on being able to look in the images before and after the image the localisation was recorded in, all localisations from the very first and last images are ignored. Similarly, as the pixel region is formed from a 21 X 21 pixel square, all localisations within 10 pixels of the edge of the image are ignored too.

You'll probably notice that this is not the fastest operation. The reason behind this is that we deliberately avoid reading too much data into memory. A typical localisation microscopy experiment may involve tens of thousands of images, and hundreds of thousands of localisations, which can easily fill Matlab's available memory – and if Matlab runs out of memory it will crash. So, we act very defensively and only ever load one image in at a time. If you know a faster, safe way to do this then make_features_from_scratch.m should be pretty straight forward to edit.

Once the features are made, they will be saved:

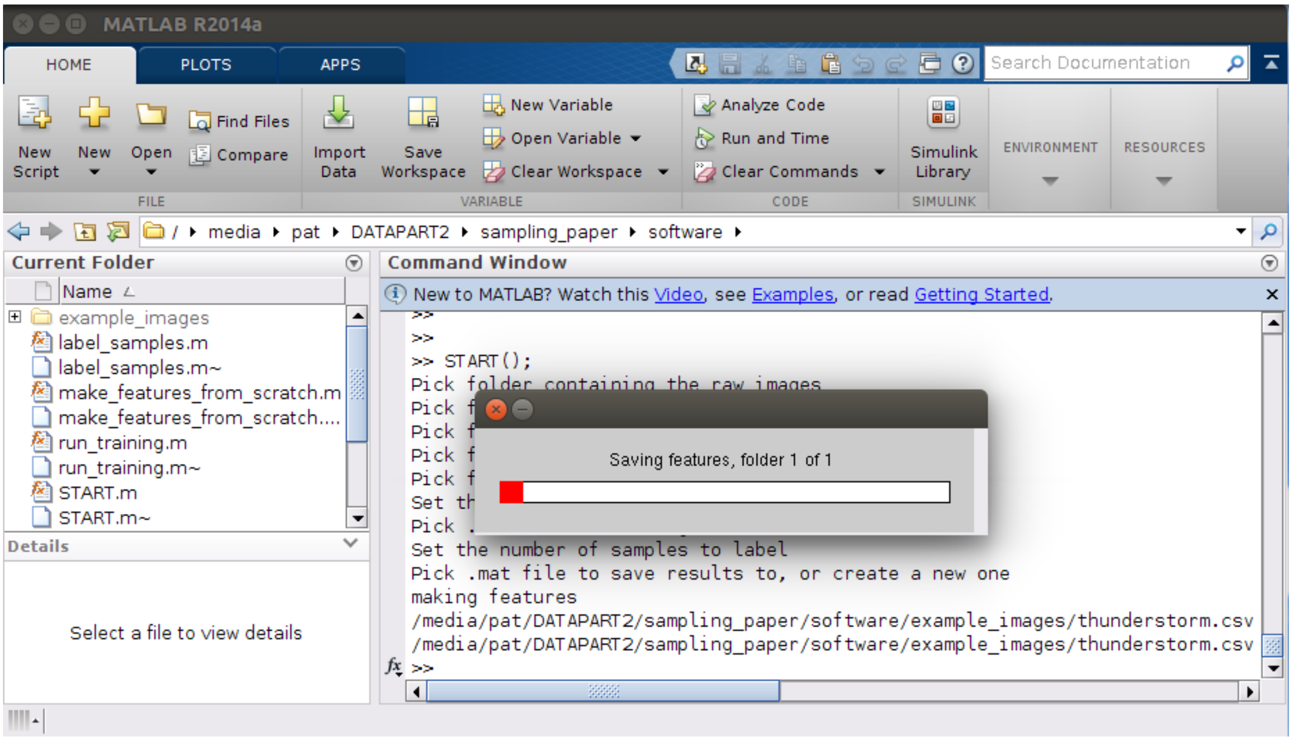

This takes about as long. When it's done you'll see something like this:

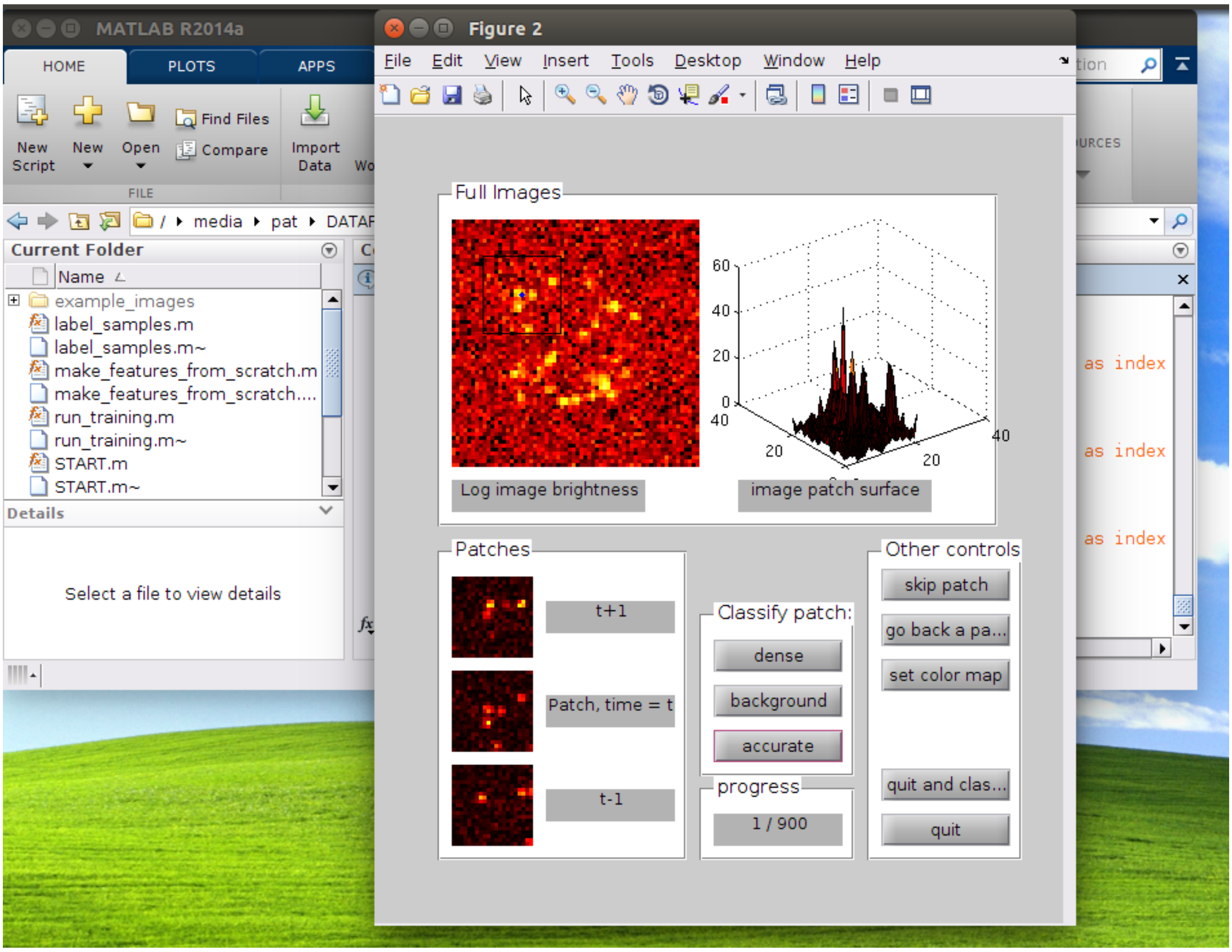

This is the labelling window, used to label each localisation. A random set of localisations will be selected from those which the algorithm identified, and you will be asked to label each one. We'll resize this and look at each part in turn, using colour coding.

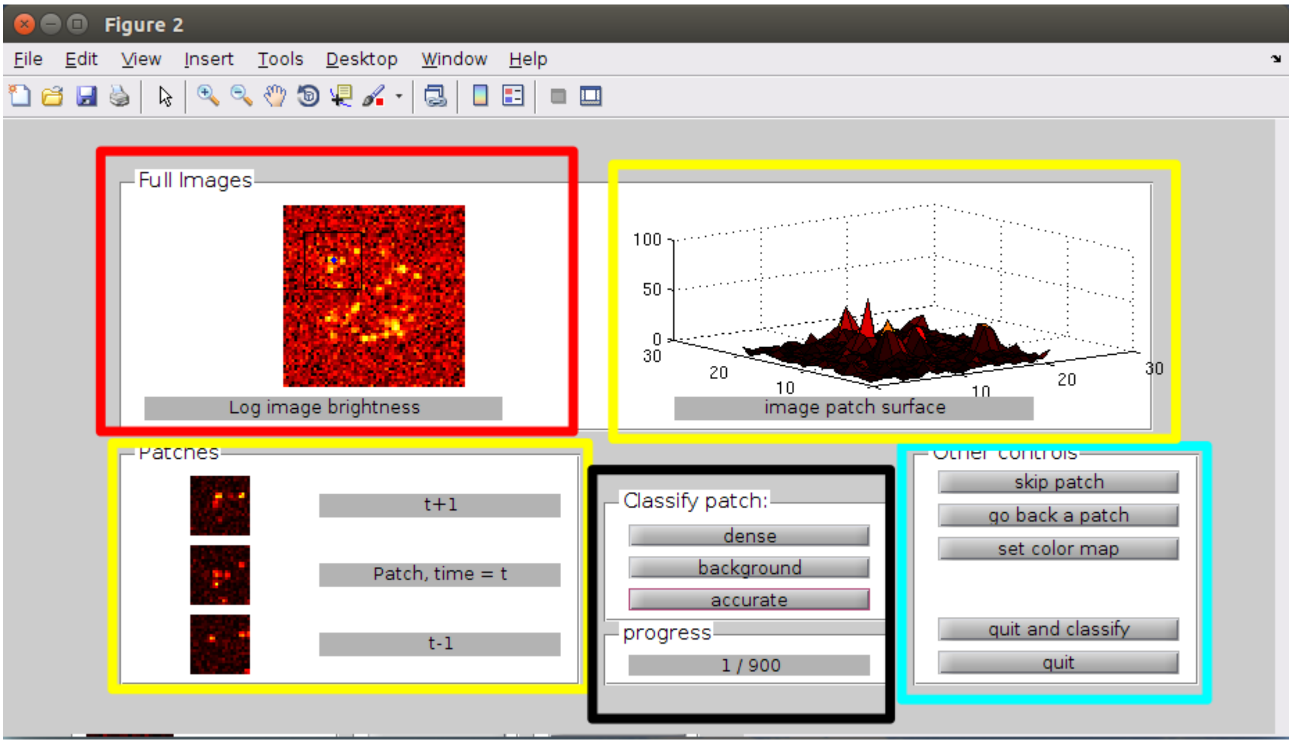

- The red box shows the entirety of the image in which the localisation was recorded. The actual position of the localisation is shown with a blue dot, and the extent of the pixel patch surrounding it is shown with a thin black line. Note that this image uses the log of the pixel intensities, rather than their raw values – this prevents bright regions of the image hiding dim fluorophores.
- The yellow boxes show the pixel patch. In the top right a surface plot of the pixel patch is shown. In the bottom left we see the patch as shown in the current image, and also the same patch areas in the images immediately before/after it.
- The black box contains the buttons you use to label each localisation, as well as the progress counter (which tells you how many localisations are still remaining to be labelled). The options are:
  - dense – there is a fluorophore present, but there is at least one other fluorophore active which is close enough that the localisation is inaccurate.
  - background – there are no clearly identifiable fluorophores present. The localisation is either due to Poisson background noise, or out of focus fluorescent material.
  - Accurate – there is exactly one fluorophore present, and you judge the localisation to be within acceptable accuracy bounds of it
- The turquoise box gives some other controls you may want to use
  - skip patch allows you to skip labelling this localisation – you will be moved onto the next one and the progress counter will not increase. Please be wary of doing this too often. If you skip all the difficult cases, then the classifier will struggle with them too.
  - Go back a patch – use this if you labelled the last patch incorrectly. You can go back and correct it
  - set color map – the default color map for all the images is Matlab's 'hot' map. If you don't like this, though, you can change it to any of the others.

    - Quit and classify – if you decide you've labelled enough localisations and want to stop early, then you can click this. A classifier will be built based solely on the fluorophores you've labelled so far.
    - Quit – quit the labelling and **don't** build a classifier. This essentially stops the whole process. Anything you've labelled so far will be lost, and to restart you'll have to re-run START()

How you choose which fluorophores are too dense, which are background and which are accurate depends on your data, your microscope setup, the behaviour of your flurorophores, and so on. The Random Forest classifier deliberately avoids building in any information about what it thinks characterises a 'background' localisation, or an 'accurate' or 'dense' one, beyond the fact it depends on the local pixel values and what the images immediately before/after the current image looked like, to avoid as much as possible any chance of introducing bias. The penalty we pay for this, however, is that it needs you to label quite a lot of samples to get enough information to learn the necessary rules.

Some tips if you'd like more guidance on how to classify:

- Try making some computer simulated fluorophores in a V-shaped structure, and the apply your localisation algorithm to it. You'll probably find that in your reconstruction the V-shape will become Y-shaped. This is due to your algorithm making errors when the fluorophores are too close together and merging them. Measure this distance on the reconstruction, and use it as a first guess as to how far apart fluorophores must be in the real dataset to be accurately localised. Depending on the fluorophore brightness, this distance can be surprisingly big – I've seen errors creeping in at 600-700nm separation, which is almost 5 pixels apart.
- Try doing the same as above, but now instead of analysing the V shape using the localisation algorithm, analyse it *by eye* yourself. See how far apart you can discern two or more fluorophores. There will be some distance below which you'll start to make errors, but so long as it's smaller than the distance your localisation algorithm makes errors, the classifier will still be useful.
- So long as you're consistent, it doesn't matter too much how you classify things. I frequently will classify localisations which appear to be a fluorophore, but which are too dim to accurately localise, as 'background'. This helps clear up noise around structures. Other people in my group don't do this – their reconstructions look different at the end, but they still make sense.
- You'll probably have to try labelling a couple of sets before you settle on a preferred way to classify things. Just save each classifier in a different .mat file as you go, so yo can refer back to them.

Personally, I'd say the below localisation should be classified as dense:

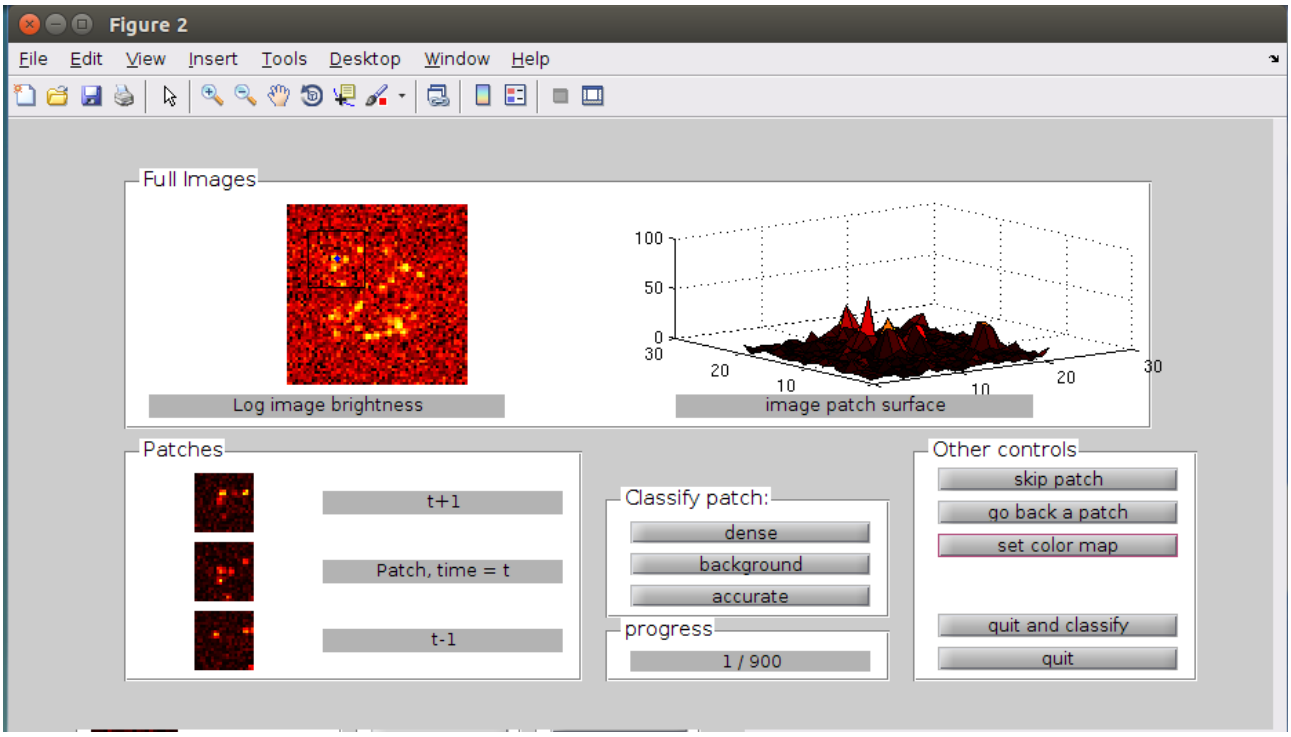

But the following might just be accurate:

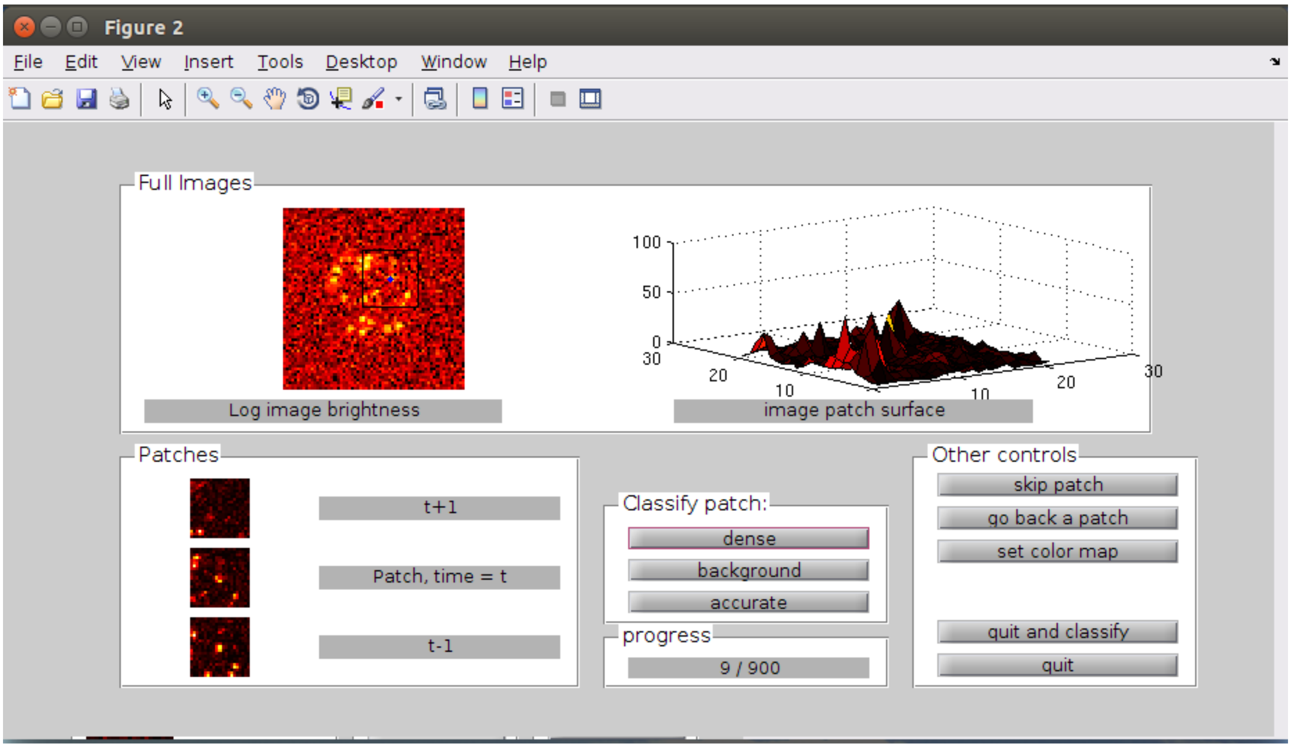

Once you've finished labelling samples, the classifier will be trained. A progress window will appear like this:

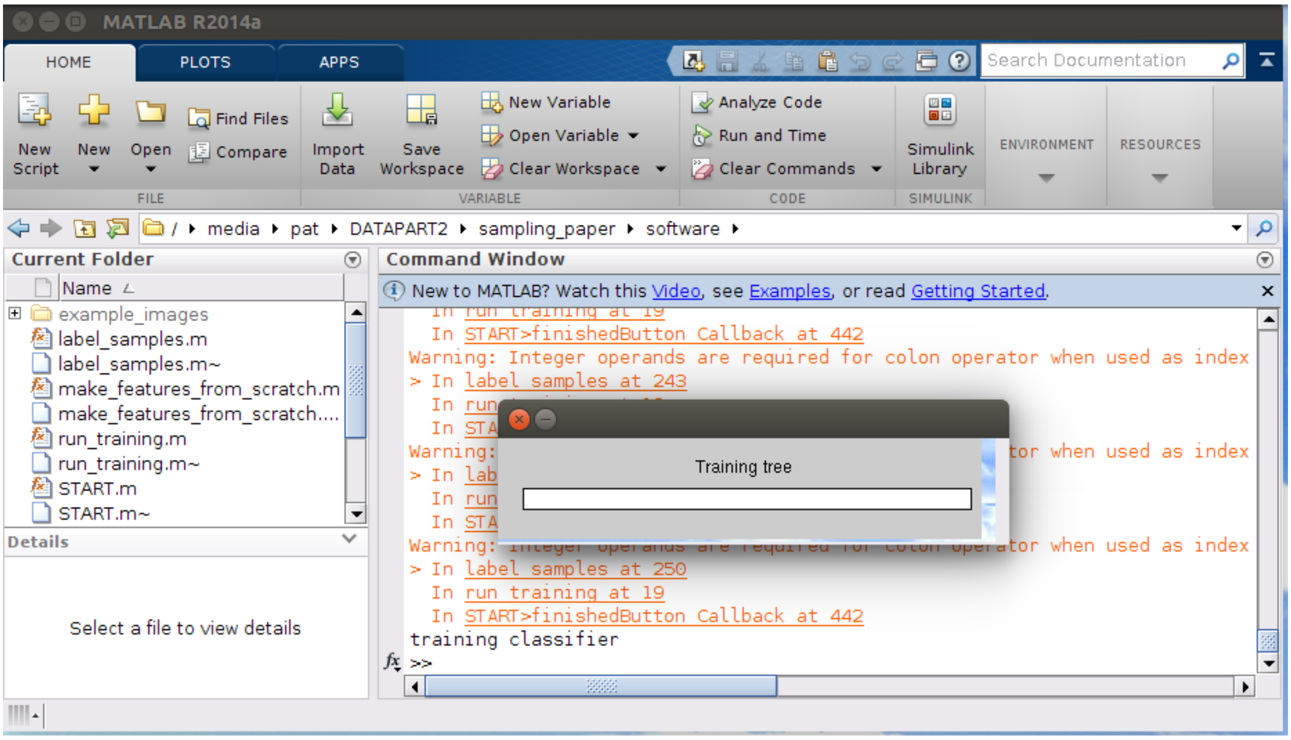

And finally, the results are saved in the .mat file whose location you specified earlier:

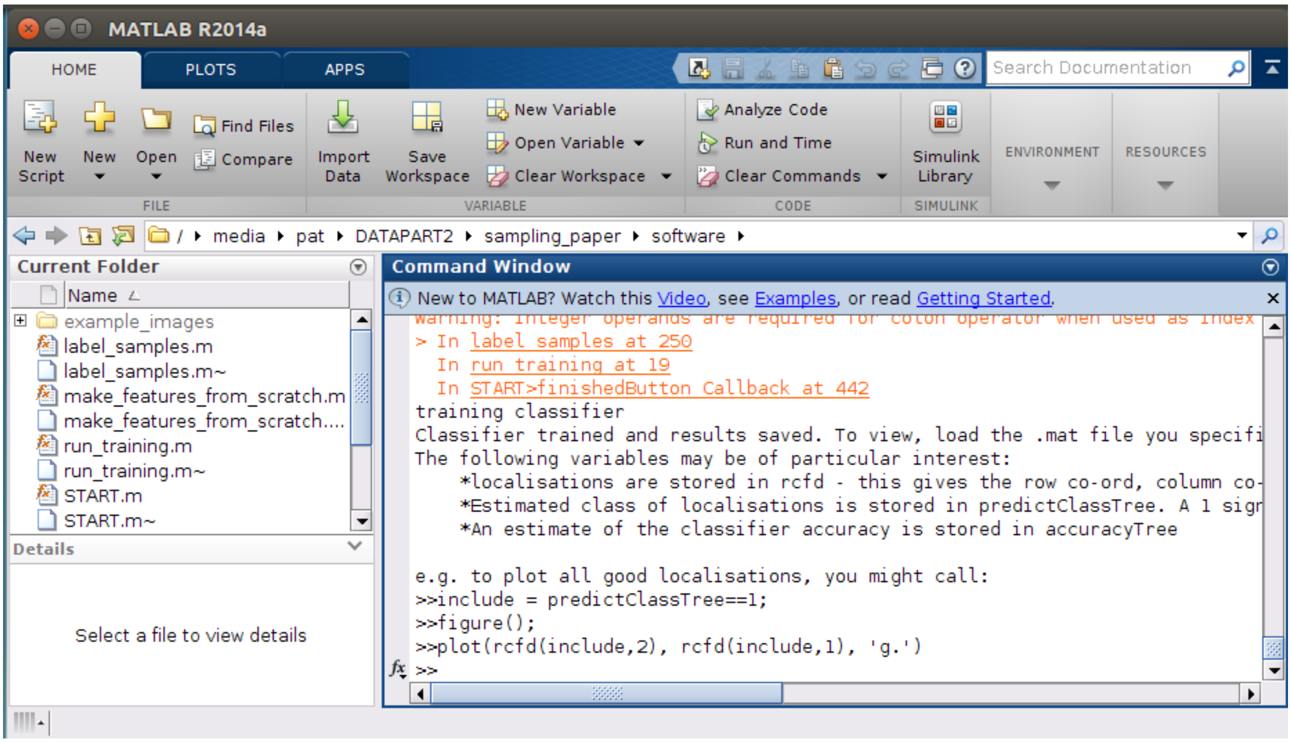

These results can then be accessed by loading the .mat file, and investigated whatever way you want. However, we also include a display function, display_results.m, which will open immediately .

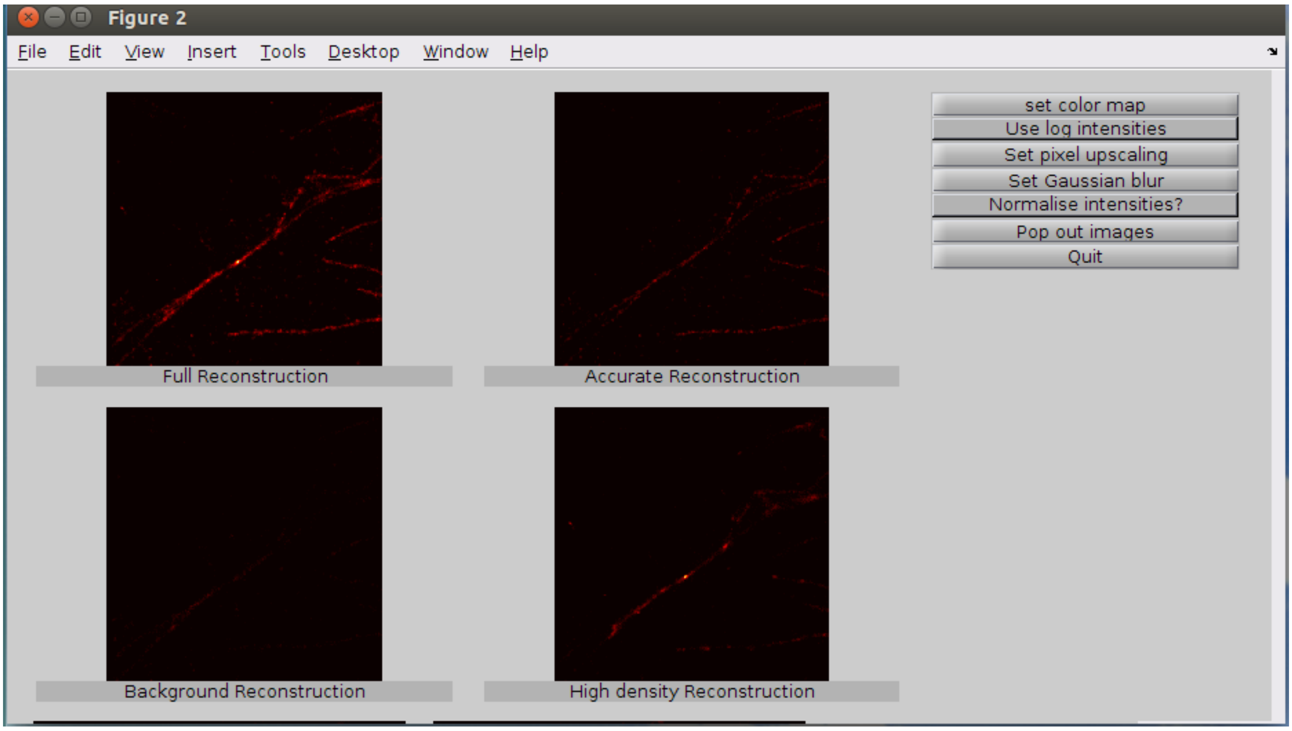

This displays 4 reconstructions of the data. Clockwise from the top left we have the reconstruction using all the localisations, the reconstruction using only those localisations classified as 'Accurate', the reconstruction using only those localisations where the density is classified as being too high, and those localisations which have been classified as 'background'.

If you have run the test dataset, this is a good time to compare your results to the ones in the paper. We have found that initially many people classify dim fluorophores as background, leading to there being almost no fluorophores classified as good localisations.

There are 7 buttons on the right hand side. The top one sets the color map, and when clicked opens a popup windows.

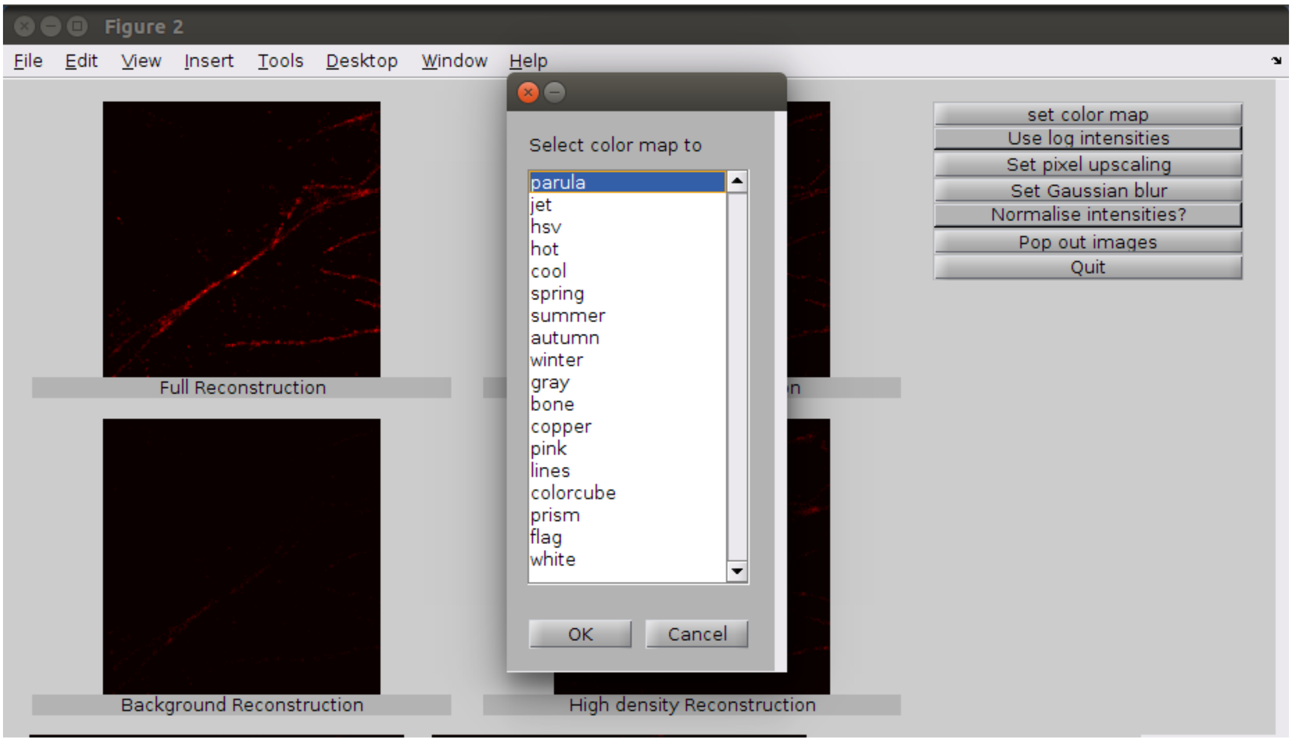

The default colour map is 'hot', but this data is a little hard to see using that. Let's change it to 'hsv'.

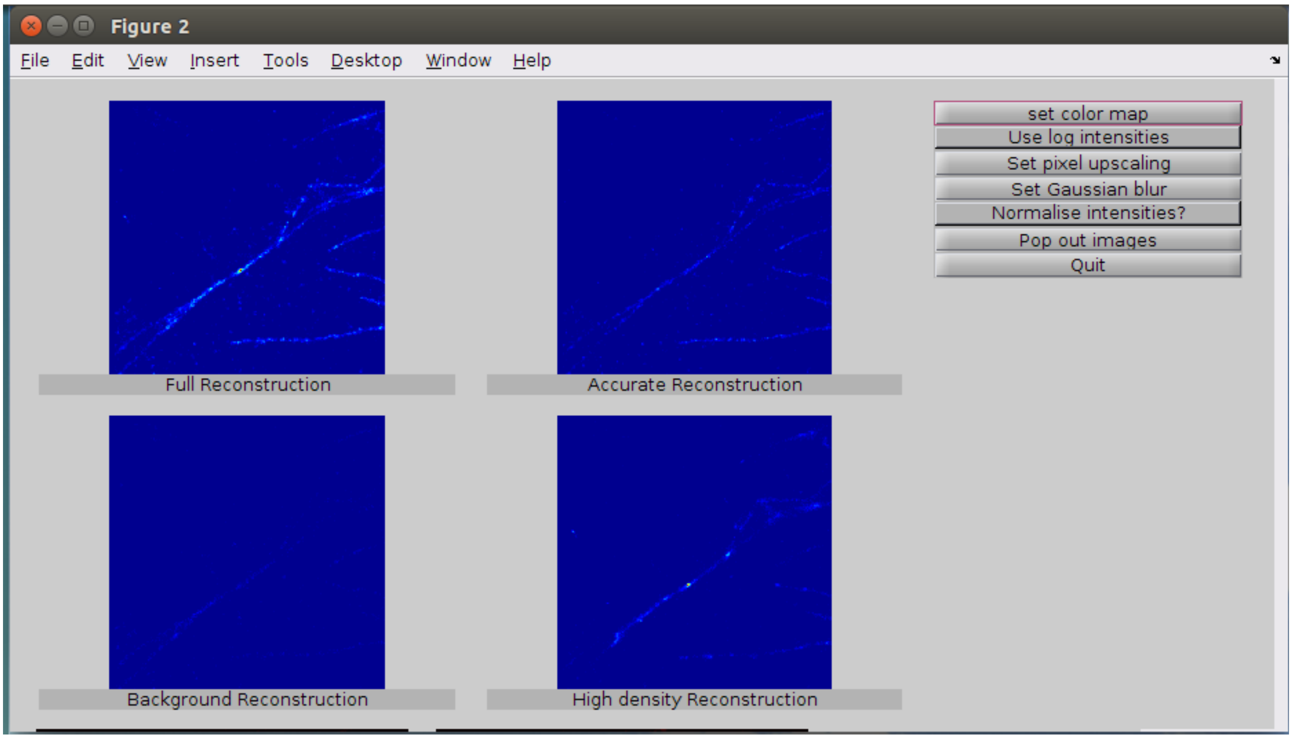

The next button allows you to toggle whether or not the reconstructions show the raw intensities, or the log of the raw intensities (with +1 added to prevent log(0) being evaluated). This reduces the relative intensity differences between bright and dim regions, and is useful for examining hard to see structure, or looking at low level background noise. When clicked it looks like this.

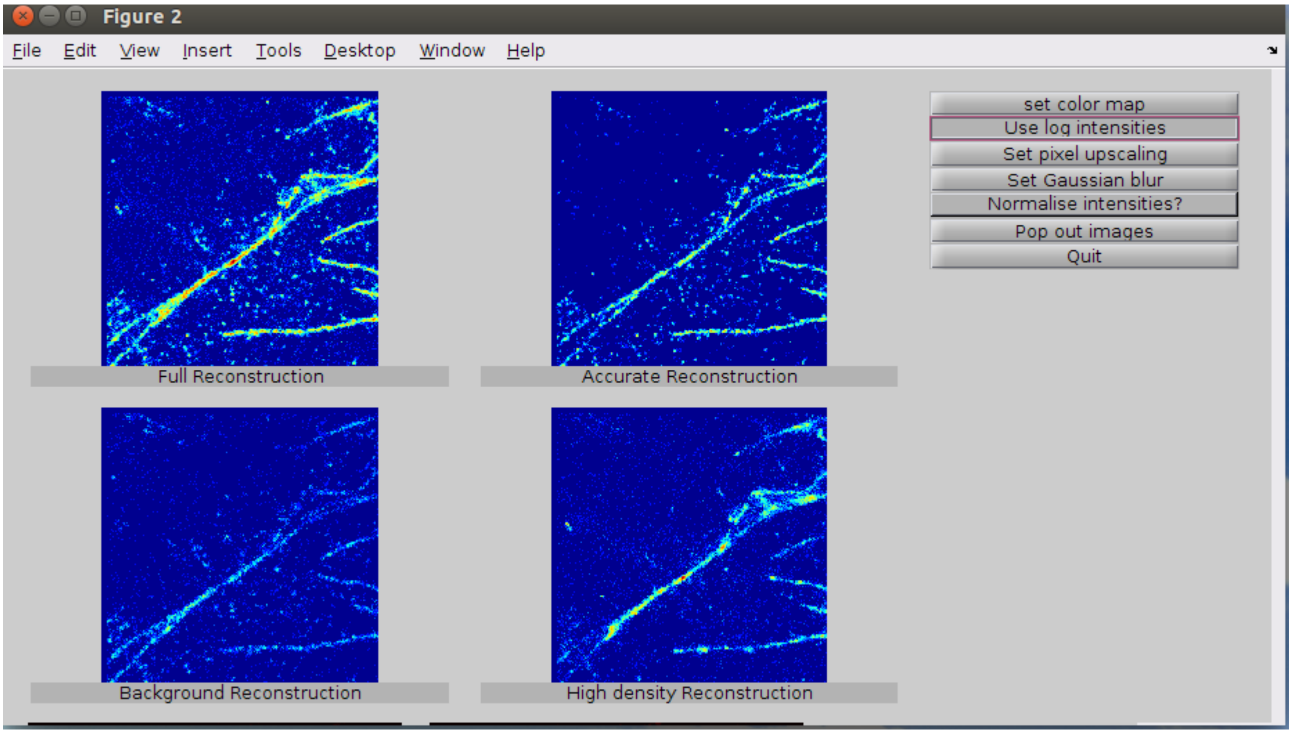

The other method for changing relative brightness is the 5th button down, 'Normalise intensities?'. The default display maintains the relative intensities between each reconstruction. As such, the full reconstruction will always appear brightest. Clicking 'Normalise intensities?' removes this constraint, and allows each image to be normalised to the same brightness range. Let's turn off the log intensity scaling, and click this instead:

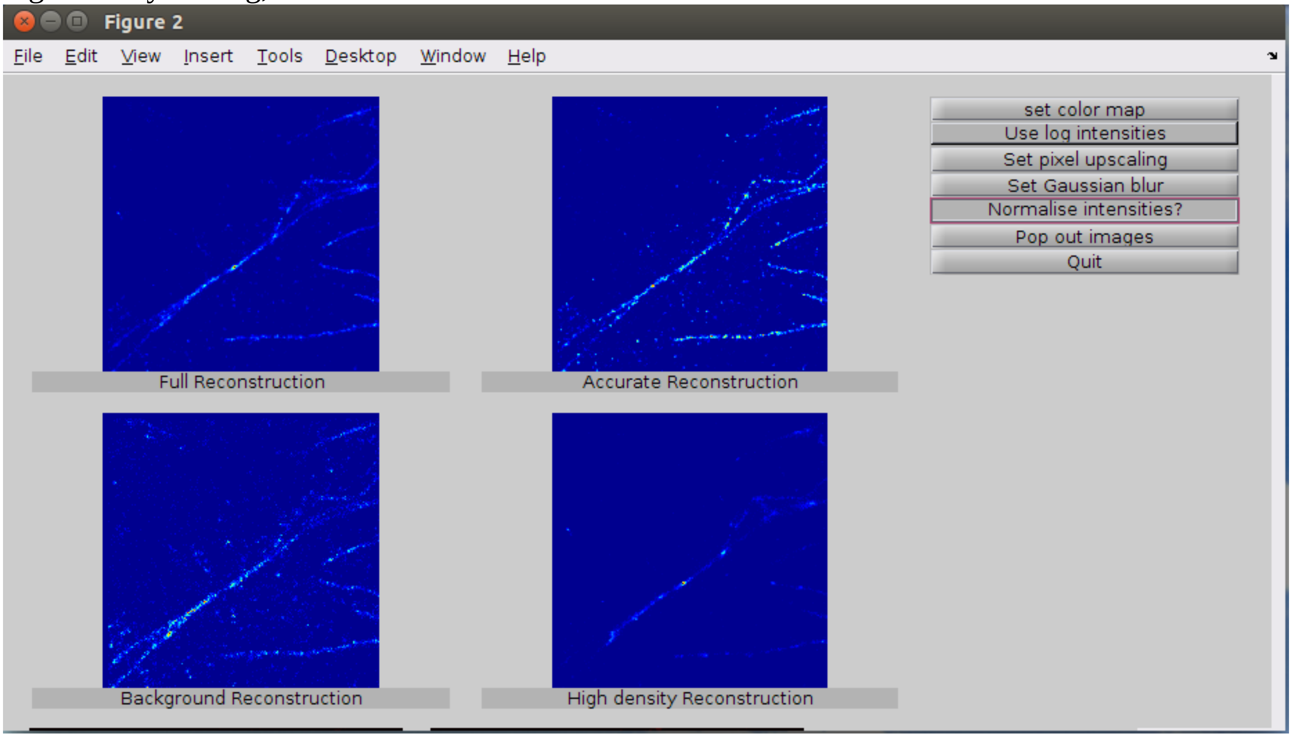

Set Pixel Upscaling controls the size of pixels used in the reconstruction. When displaying the reconstruction, to examine the super resolution detail, it is generally necessary to use a smaller pixel size than that of the camera used to collect the data. The pixel upscaling value sets the relative size of the reconstructed pixels compared to the original pixels. The default value is 5, so a single pixel in the reconstruction is 1/5th the size of the original pixels. This can be changed by clicking 'set pixel upscaling'

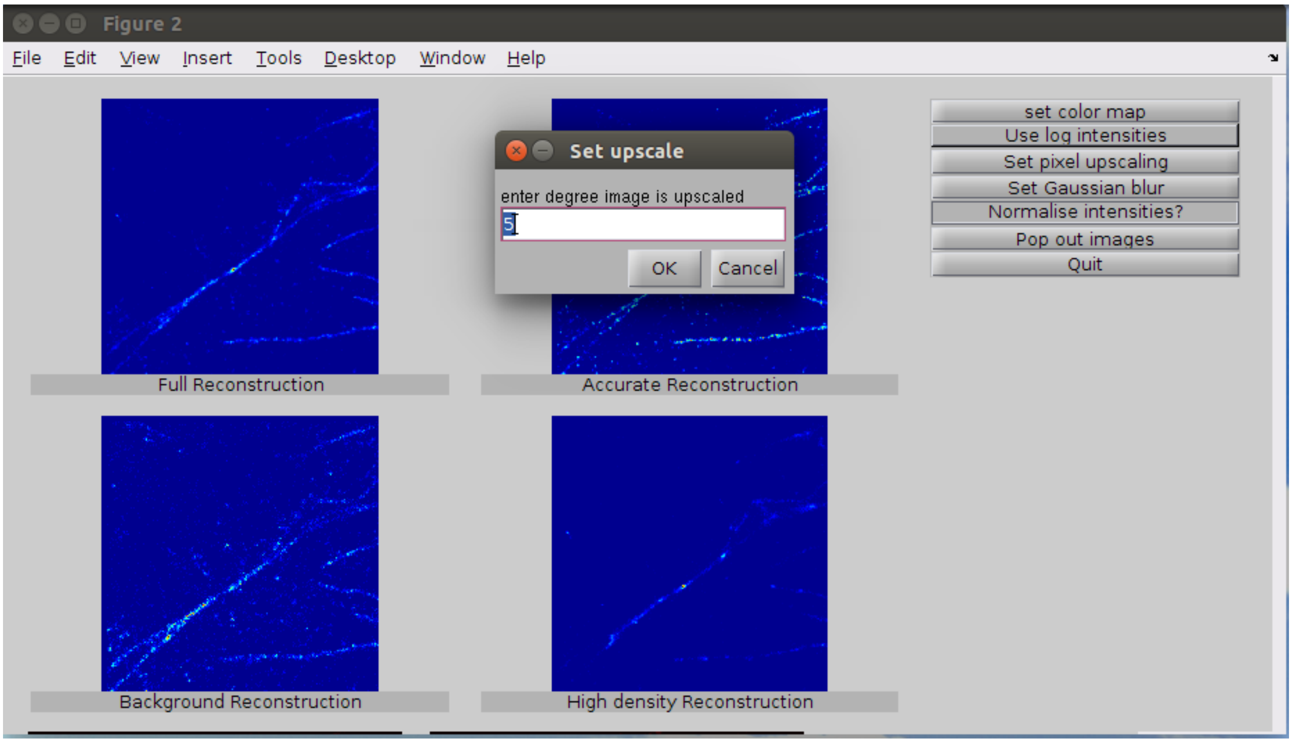

We'll set this to 10 instead, so the reconstructed pixels are smaller.

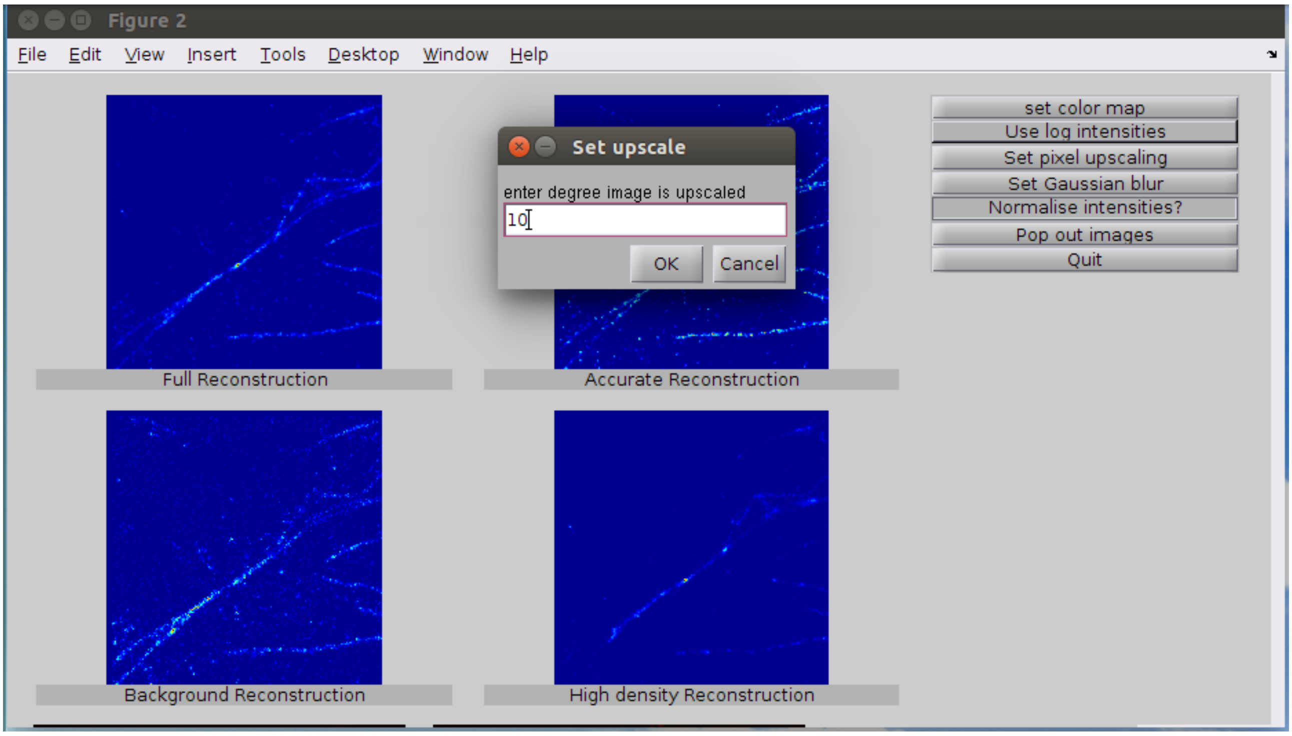

Examining the images after this, the reconstruction looks a bit pixellated. A more visually pleasing result can often by found by blurring the image slightly. This is controlled by the 'Set Gaussian Blur' button:

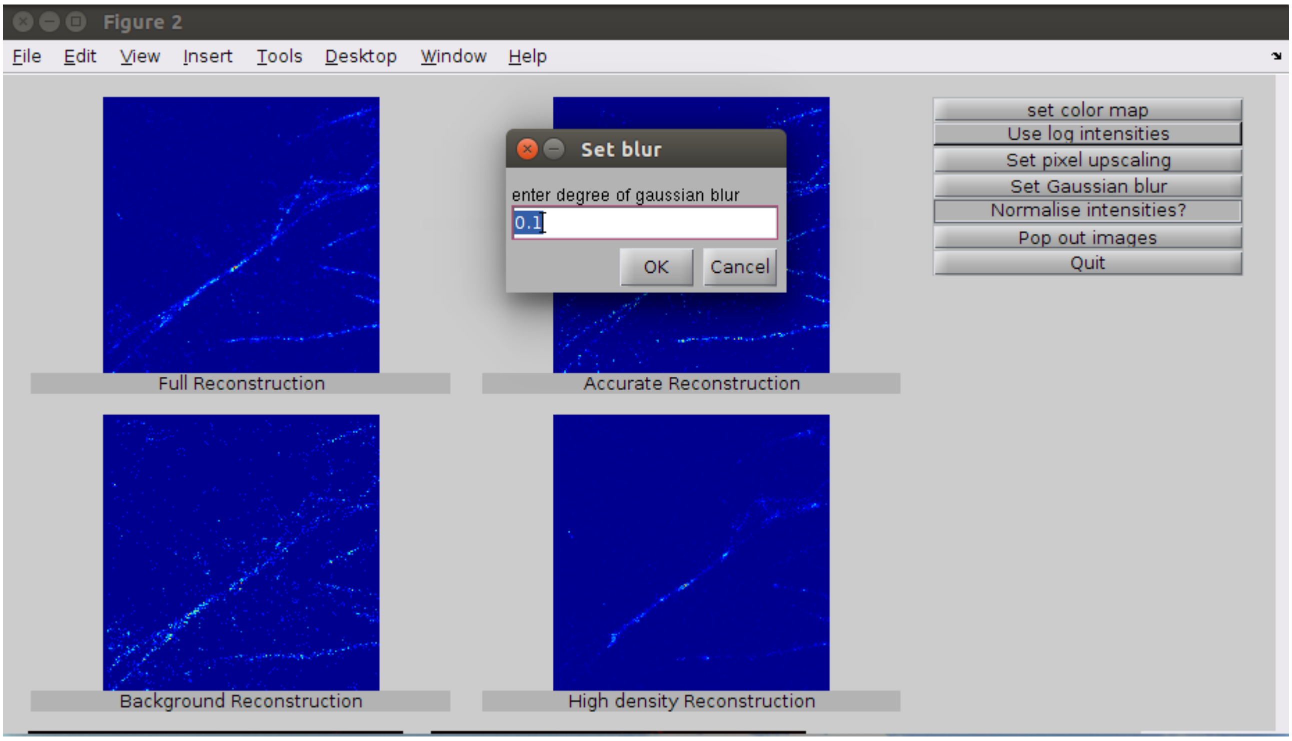

All images in this display have at least some Gaussian blur applied. The default standard deviation is set at 0.1 pixels (NB this uses the pixel size in the *reconstruction*, and not the pixel size of the *original data*). This can be changed to increase/reduce the blur. We'll try increasing it slightly, to reduce the pixellation

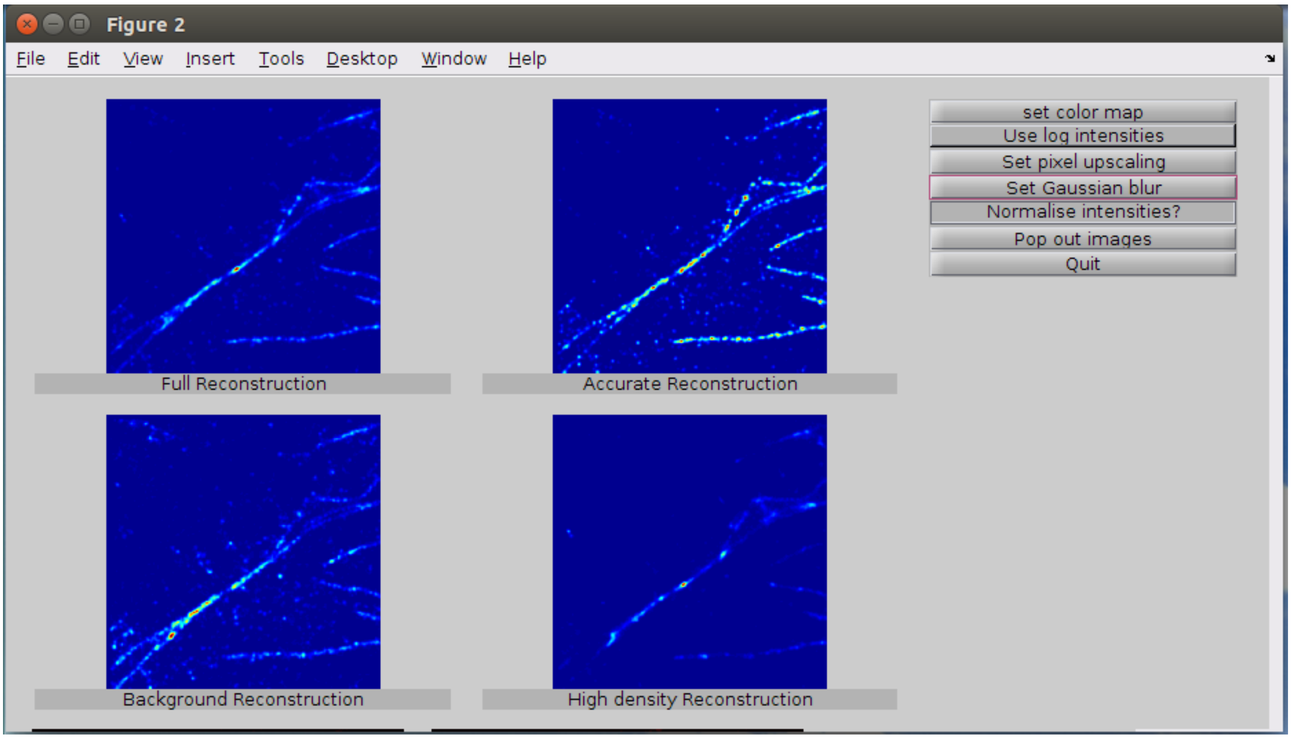

Once you're happy with how the images look, if you wish you can generate a separate figure for each. This is done by pressing the 'Pop out images' button. Four new figure windows will be created, each one showing one of the four figures in the original display figure.

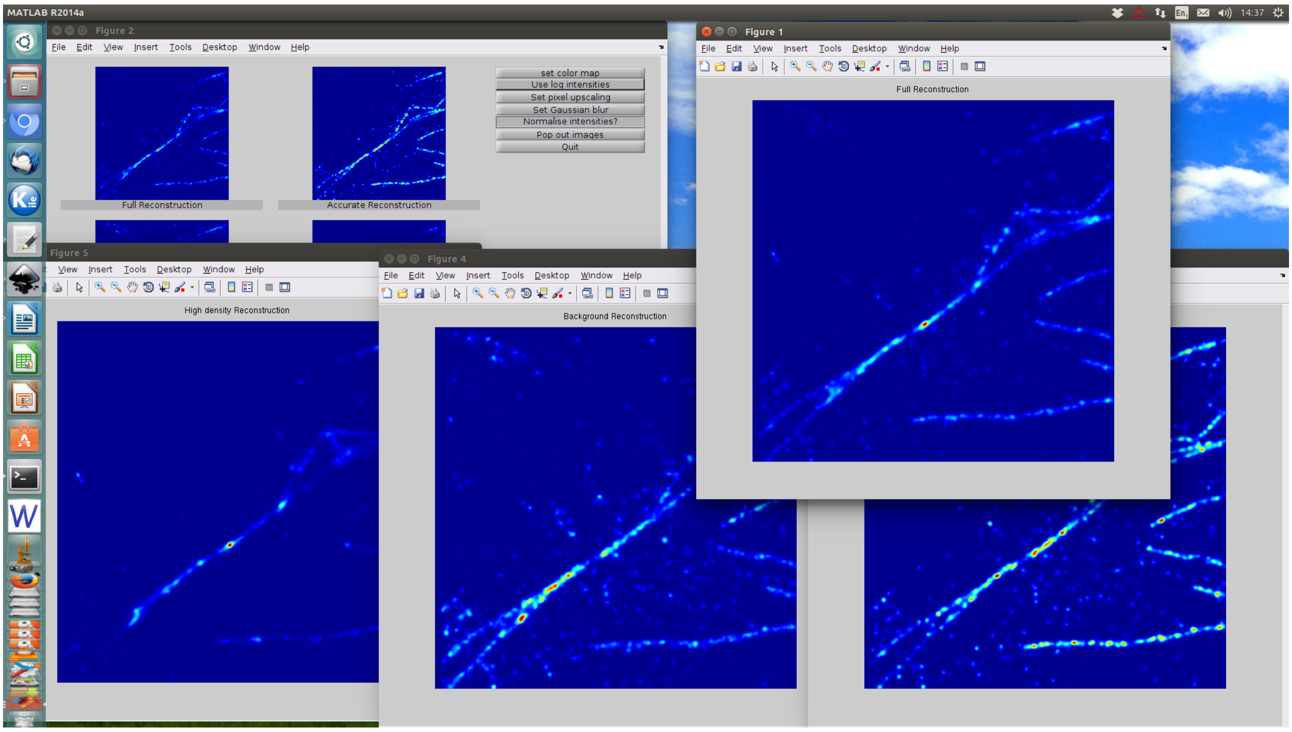

These can now be saved in the standard way for a Matlab image, e.g. by selecting file > save as and then choosing your save location.

To quit, click 'Quit'

If you quit then decide you want to open the display function again, you can simply reload the data into Matlab. This can be done using by typing:
DISPLAY();
at the Matlab prompt. This will return you to the initial display stage of the results.
